# Multiepitope Subunit Vaccine Design against COVID-19 Based on the Spike Protein of SARS-CoV-2: An *In Silico* Analysis

**DOI:** 10.1155/2020/8893483

**Published:** 2020-11-19

**Authors:** Hamza Arshad Dar, Yasir Waheed, Muzammil Hasan Najmi, Saba Ismail, Helal F. Hetta, Amjad Ali, Khalid Muhammad

**Affiliations:** ^1^Foundation University Medical College, Foundation University Islamabad, Islamabad 44000, Pakistan; ^2^Department of Internal Medicine, University of Cincinnati College of Medicine, 231 Albert Sabin Way, Cincinnati, OH 45267-0595, USA; ^3^Department of Medical Microbiology and Immunology, Faculty of Medicine, Assiut University, Assiut 71515, Egypt; ^4^Atta-ur-Rahman School of Applied Biosciences, National University of Sciences and Technology, Islamabad 44000, Pakistan; ^5^Department of Biology, College of Science, United Arab Emirates University, Al Ain 15551, UAE

## Abstract

The global health crisis caused by severe acute respiratory syndrome coronavirus 2 (SARS-CoV-2), the causal agent of COVID-19, has resulted in a negative impact on human health and on social and economic activities worldwide. Researchers around the globe need to design and develop successful therapeutics as well as vaccines against the novel COVID-19 disease. In the present study, we conducted comprehensive computer-assisted analysis on the spike glycoprotein of SARS-CoV-2 in order to design a safe and potent multiepitope vaccine. *In silico* epitope prioritization shortlisted six HLA I epitopes and six B-cell-derived HLA II epitopes. These high-ranked epitopes were all connected to each other via flexible GPGPG linkers, and at the N-terminus side, the sequence of Cholera Toxin *β* subunit was attached via an EAAAK linker. Structural modeling of the vaccine was performed, and molecular docking analysis strongly suggested a positive association of a multiepitope vaccine with Toll-like Receptor 3. The structural investigations of the vaccine-TLR3 complex revealed the formation of fifteen interchain hydrogen bonds, thus validating its integrity and stability. Moreover, it was found that this interaction was thermodynamically feasible. In conclusion, our data supports the proposition that a multiepitope vaccine will provide protective immunity against COVID-19. However, further in vivo and in vitro experiments are needed to validate the immunogenicity and safety of the candidate vaccine.

## 1. Introduction

The unprecedented global health crisis due to the COVID-19 pandemic, with associated economic and social problems, poses a great threat to the world order today [1, 2]. Wuhan city in China reported the first case of the novel coronavirus disease; however, the disease progressed quickly from there and has now spread to a total of 213 countries [3]. Currently, more than 16 million people worldwide have been reportedly infected, while more than 0.6 million infected patients have died unfortunately.

COVID-19 patients typically show a variety of symptoms—from mild symptoms to severe illness. Common symptoms include dry cough, fever, shortness of breath, fatigue, and upper airway congestion [4]. After virus exposure, it may take 2-14 days for the symptoms to appear [5]; however, some infected people remain asymptomatic [6, 7]. The virus is spread mainly from person-to-person due to close contact (within ~6 feet distance), through small air droplets [8]. Some studies suggest that the virus could be transmitted from asymptomatic persons to others [9, 10].

SARS-CoV-2 is implicated in COVID-19 pathology. Its genome organization is thought to be similar to that of other human coronaviruses [11]. The genome of SARS-CoV-2 is composed of RNA which serves as mRNA and contains open reading frames (ORFs) ORF1ab, ORF3a, ORF6, ORF7a, ORF8, and ORF10; spike glycoproteins; nucleoprotein; membrane protein; and envelope protein E [12]. The spike proteins protrude from the surface of SARS-CoV-2. These spikes come into contact with human cells and are subjected to conformational changes that in turn facilitate the fusion of the membrane of the virus with the human cell membrane [13]. This leads to viral entry inside human cells. SARS-CoV-2 spikes bind to human cell surface angiotensin-converting enzyme 2 (ACE2) receptors to enter human cells [14]. Moreover, the spike protein induces humoral and cellular immune responses against SARS-CoV-2 [15]. Therefore, the spike protein is a suitable antigenic target for designing efficient therapeutics against COVID-19.

Some studies explored the recombinant spike protein-based vaccine design against COVID-19. Notably, Keech et al. recently performed Phase 1–2 trials of a SARS-CoV-2 recombinant spike protein-based nanoparticle vaccine and obtained good results [16]. Ren et al. assessed the immunogenic properties of CHO-expressed recombinant SARS-CoV-2 S1-Fc fusion protein in rabbits, mice, monkeys, and rabbits as a potential anti-COVID-19 vaccine candidate [17]. Their findings indicated that fusion protein is highly immunogenic.

The spike protein has also been subjected to epitope prediction by numerous researchers. Chen et al. focused on the prediction of B-cell and T-cell epitopes in the SARS-CoV-2 spike and nucleocapsid proteins [18]. Further, evaluation of these epitopes was done to prioritize some epitopes. Li et al. conducted an immunoinformatics study on the SARS-CoV-2 surface glycoprotein, membrane glycoprotein, and nucleocapsid proteins to forecast putative B-cell and T-cell epitopes [19]. These were then filtered as per features like toxicity, antigenicity, physiochemical characterization, and stability. Ahmed et al. screened the experimentally reported SARS-CoV B-cell and T-cell epitopes of SARS-CoV to identify those epitopes present in the spike and nucleocapsid proteins that displayed 100% identity to SARS-CoV-2 proteins [20].

Currently, there are no effective drugs or vaccines available against COVID-19. Considering the gravity of the situation globally, an effective vaccine or antiviral drug is the need of the hour. Reverse vaccinology is the genome-based mining of pathogenic microorganisms to identify suitable targets to guide the development of vaccines [21, 22]. In this study, we used a computer-assisted vaccine design approach against COVID-19 by combining the approaches used in reverse vaccinology, immunoinformatics, and structural bioinformatics. *In silico* epitope mapping and prioritization were done on the spike protein of novel coronavirus species to select promising T-cell (CD4+ and CD8+) and B-cell epitopes. All the prioritized epitopes were later incorporated in a single multiepitope vaccine.

We conducted three-dimensional structural modeling of a candidate vaccine using bioinformatic tools and evaluated its effectiveness. The selected model was then used in molecular docking studies with Toll-like Receptor 3 (TLR3) to characterize its ability to trigger specific immunological responses against the novel coronavirus disease. TLR3 acts as a sensor of viral infection and activates downstream innate immunity-related signaling pathways [23]. Studies on SARS-CoV and MERS indicate the role of TLR3 in the development of a protective response against coronaviruses [24, 25]. The vaccine-TLR3 complex was also analyzed for bonding interactions. Moreover, the energetic feasibility of this molecular interaction and binding affinity were checked to assess the sustainable binding potential of the candidate vaccine with the human TLR3 receptor.

## 2. Materials and Methods

**Figure 1:**
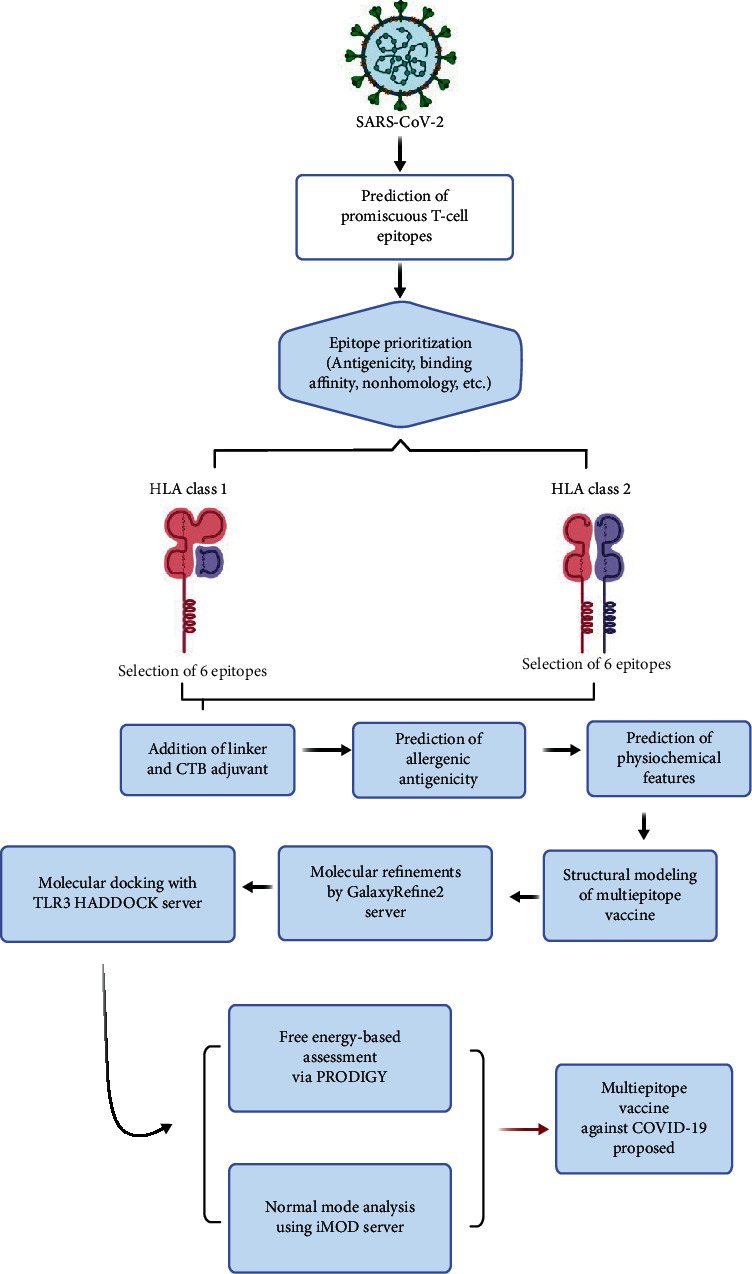
Here, the overall design of our study is shown. The sequence of SARS-CoV-2 spike protein was subjected to *in silico* epitope prediction and prioritization steps to select promiscuous T-cell epitopes. These epitopes were linked together to construct a multiepitope vaccine which was subjected to molecular docking analysis with TLR3. Based on these results, a multiepitope vaccine was proposed against COVID-19.

The overall methodology is illustrated in Figure 1.

### 2.1. Retrieval of SARS-CoV-2 Spike Surface Glycoprotein Sequence

The full-length amino acid sequence (FASTA format) of the surface glycoprotein of the SARS-CoV-2 reference genome (NC_045512.2) with accession number YP_009724390.1 was downloaded from the NCBI GenBank database.

### 2.2. Prediction of HLA Class I Interacting Epitopes

The sequence of the full-length surface glycoprotein YP_009724390.1 was fed into the NetCTL 1.2 server to check the binding affinity with twelve specified HLA supertypes: A1, A2, A3, A24, A26, B7, B8, B27, B39, B44, B58, and B62 [26]. Default settings were applied; however, the peptides were scored as per the combined score. Only those epitopes that showed interaction with at least four HLA I supertypes were shortlisted at this stage. These were subjected to immunogenicity checks by the IEDB server in order to identify potentially immunogenic epitopes [27]. The tool is validated for 9mer peptides, although peptides with any length can be submitted to the server. Further, the epitopes were analyzed for antigenicity properties using VaxiJen version 2.0 to identify antigenic epitopes with scores greater than 0.4 [28]. BLASTp of resulting epitopes was done against the human genome to discard those with autoimmunity potential where applicable. The prioritized epitopes were submitted to the IEDB server to check whether they had been experimentally tested by other studies or not. To cross-check the finalized epitopes, the ProPred 1 server (default) was also used selecting all alleles to confirm the prediction performance by NetCTL 1.2 [29].

### 2.3. Prediction of HLA Class II Interacting Epitopes

The sequence of full-length surface glycoprotein (YP_009724390.1) was subjected to HLA II epitope prediction by NetMHCIIpan. Prediction was performed specifically to hunt epitopes binding to the following HLA II supertype alleles: DRB1∗0101, DRB1∗0301, DRB1∗0401, DRB1∗0701, DRB1∗0801, DRB1∗1101, DRB1∗1301, and DRB1∗1501 [30]. These HLA II supertype alleles encompass variants of HLA of ~95% of the global human population [31]. NetMHCIIpan is currently the most accurate tool for HLA II epitope prediction, which is why it was used in this study [32]. Another tool, PREDIVAC, was used with the same eight alleles using default settings [33].

All the epitopes found to interact with at least three supertype alleles (four supertype alleles in the case of PREDIVAC) were checked for their antigenic properties using VaxiJen version 2.0, and only those with antigenicity scores greater than 0.4 were retained [28]. Next, their sequences were submitted to the MHCPred 2.0 server to evaluate their binding affinity with most common allele, DRB1∗0101 [34]. In the case of 15-mer peptides obtained from NetMHCIIpan, the epitopes derived were subjected to analysis to confirm the interaction potential of the parent 15-mer peptides. Those peptides showing IC50 values below 500 nm were positively selected as this value is associated with a high level of immunogenicity [35, 36]. Next, overlapping peptides were identified and, based on parameters like antigenicity, IC50 values, and conservation level, only the best were retained for further study.

All the peptides obtained were subjected to BLASTp against the human genome to identify those with homology to any region in the human genome so they could be discarded to avoid the risk of autoimmunity. Meanwhile, the glycoprotein sequence was also submitted to the iBCE-EL web server to predict linear B-cell epitopes [37]. All the prioritized HLA II peptides were checked to see whether they shared an overlap with putative B-cell epitopes. Finally, the shortlisted epitopes were submitted to the IEDB server to find out whether they had been studied before in relation to COVID-19 disease.

### 2.4. Multiepitope Vaccine Design

Both the HLA I epitopes and B-cell-derived HLA II epitopes were checked to ensure that there was no overlapping of any kind. Amongst the overlapping peptides, only the most appropriate epitopes were selected to proceed towards multiepitope vaccine design. These finalized epitopes were joined together via flexible GPGPG linkers. This step was essential for effective separation of the epitope domains and for limiting the chances of forming junctional epitopes [38]. Moreover, to improve the overall immunogenicity of multiepitopes, the Cholera Toxin *β* subunit (CTB) sequence was incorporated at the N-terminal side of the vaccine construct. A linker sequence of EAAAK joined the adjuvant to the first epitope in the designed construct. CTB, a nontoxic component of cholera toxin, exhibits good affinity towards monosialotetrahexosylganglioside on the surface of the gut epithelium as well as standard antigen-presenting cells such as dendritic cells, B-cells, and macrophages [39]. This is a highly desired feature of our vaccine which will permit the maximum accessibility and contact with the immune machinery inside the human body.

### 2.5. Sequence Analyses for Physicochemical, Antigenicity, Solubility, and Safety Checks

The multiepitope protein sequence was then submitted to the Protparam tool at ExPASy to calculate physicochemical features such as molecular weight, isoelectric point (pI), half-life, grand average of hydropathicity (GRAVY), aliphatic index, and instability index [40, 41]. For the estimation of antigenic properties, highly accurate tools, such as ANTIGENPro and VaxiJen version 2.0, were used [28, 42]. Cross-validation experiments portray the high accuracy of ANTIGENPro (76%) on the combined dataset; meanwhile, the performance accuracy of VaxiJen is known to lie between 70 and 89%, as per the target organism selected. To predict the solubility of our designed vaccine, we used two tools: SOLpro and ccSol [43–45]. SOLpro is known to have an estimated accuracy of ~74%, calculated using more than one run of 10-fold cross-validation. ccSol, another prediction tool, has 76% accuracy.

The allergenicity of the multiepitope vaccine was assessed through two servers: AllergenFP 1.0 plus AllerTOP 2.0 [46, 47]. AllergenFP version 1.0 can be used to identify both allergens and nonallergens with an accuracy of 87.9% [47]. The performance review of allergenicity prediction tools suggests that AllerTOP version 2.0 is the top-performing method with an accuracy of 88.7%, followed by AllergenFP 1.0 (87.9% accuracy). The multiepitope sequence was also subjected to BLASTp against the human genome to ascertain potential homology to humans.

### 2.6. Structural Modeling and Molecular Refinements of the Multiepitope Vaccine

The three-dimensional structural model of the polyepitope vaccine was modeled by a 3Dpro tool [43]. It was then submitted to the GalaxyRefine2 webserver to refine local inaccurate regions and to improve the structure as a whole [48]. Each of the ten resulting models was assessed on the basis of Ramachandran plot analysis, GALAXY energy score, and RMSD values. To corroborate the structural properties of the selected model, the ERRAT and ProSA web servers were used [49, 50].

### 2.7. Prediction of Discontinuous B-Cell Epitopes in Multiepitope Vaccine

The three-dimensional structure of the refined model of our polyepitope subunit vaccine was subjected to discontinuous B-cell epitope prediction. For this purpose, two servers, Ellipro and DiscoTope version 2.0, were used with default parameters [51, 52]. The resulting discontinuous B-cell epitopes were identified and located (mapped) on the three-dimensional structure of the vaccine.

### 2.8. Binding Affinity Analysis of Vaccine with Toll-like Receptor 3

The binding affinity of the polyepitope vaccine with TLR3 was analyzed using the PRODIGY web server [53]. PRODIGY provides the output in terms of Gibbs free energy (*∆G*), which is an important thermodynamic parameter to assess whether the binding affinity in a biological complex is energetically feasible or not [54]. Usually, negative values indicate the greater likelihood of biomolecules to form a biologically plausible complex.

### 2.9. Normal Mode Analysis of Vaccine-TLR3 Complex

Normal mode analysis was conducted in order to confirm the stability of protein-protein complexes after using HADDOCK. The stability of the protein(s) can be gauged by comparing their dynamic behavior with normal modes [55, 56]. It is an important method that could serve as an alternative to computationally intensive all-atom molecular simulations [57, 58]. By carefully looking at the normal modes associated with internal coordinates, the overall motion of proteins was studied [59]. The approach used is known to yield effective outcomes in less time compared to molecular dynamic (MD) simulations that are in much use [60, 61]. The iMOD server was thus used to study the protein complex-related movements considering important parameters like eigenvalues, *B*-factors, covariance, and deformability. The deformation of protein chain/s depends on the deformability at the residue level. The eigenvalue associated with each normal mode tells us information about the motion stiffness. This also provides crucial insights into the energy needed to deform the protein(s) structure(s). The easier deformation is clearly indicated by low eigenvalues [62].

### 2.10. Reverse Translation and Codon Optimization of the Multiepitope Vaccine

In order to obtain the best codon-optimized sequence for expression in the *E. coli* K12 strain, the protein sequence of the candidate vaccine was subjected to reverse translation followed by codon optimization. For this purpose, the JCat online server was used [63]. Care was taken to steer clear of rho-independent transcription terminators, as well as sites related to prokaryote binding and restriction enzyme recognition. The GC content and Codon Adaptation Index of the optimized sequence was analyzed accordingly.

## 3. Results

### 3.1. Identification of Potential HLA I Interacting Epitopes in SARS-CoV-2 Spike Glycoprotein

A total of eighteen epitopes belonging to SARS-CoV-2 spike glycoprotein were projected to have strong binding interactions with at least four HLA I subtypes. These epitopes, as well as the names of their associated HLA supertypes and total number of supertypes, are given in Table S1 sheet 1. After immunogenicity screening of these peptide sequences, we found eleven epitopes with positive immunogenicity scores (Table S1 sheet 2). Out of these, seven epitopes were predicted to be antigenic, so these were retained accordingly (Table S1 sheet 3).

BLASTp analysis of the shortlisted peptides against the human genome did not reveal homology of these sequences to any human genomic region. Further, it was confirmed that these epitopes had not been experimentally tested previously and thus represent novel predictions. It was found that ProPred I also classified these peptides as potential HLA I epitopes [29].

### 3.2. Identification of Potential HLA II Interacting Epitopes in SARS-CoV-2 Spike Glycoprotein

A total of fifteen HLA II epitopes corresponding to SARS-CoV-2 spike glycoprotein were predicted to show positive associations with at least four specified HLA DRB alleles using the PREDIVAC tool (Table S2 sheet 1) [33]. However, only five were antigenic according to VaxiJen criteria (Table S2 sheet 2) [28]. These epitopes showed IC50values < 500nm with the DRB1∗0101 allele and showed at least 95% conservation amongst SARS-CoV-2 sequences.

Meanwhile, a total of fourteen 15-mer putative HLA II epitopes in the SARS-CoV-2 spike glycoprotein were predicted to associate with at least three specified HLA DRB alleles using NetMHCIIpan version 3.2 (Table S3 sheet 1) [30]. Of these, seven epitopes had an antigenicityscore > 0.4 using VaxiJen and were retained (Table S3 sheet 2) [28]. However, four of them showed an overlap with each other while the rest overlap with each other. So, based on the analysis of IC50 value with the DRB1∗0101 allele, antigenicity, and conservation level, two nonoverlapping 15-mer peptides were shortlisted (Table S3 sheet 3).

In total, seven HLA II epitopes were shortlisted using the abovementioned tools (five obtained using PREDIVAC and two using NetMHCIIpan version 3.2). After checking the epitopes in the IEDB database, it was confirmed that none of them had been experimentally tested previously concerning the COVID-19 disease, thus indicating novelty. It was confirmed that all of them shared an overlap with B-cell epitopes predicted by the iBCE-EL tool (Table S4). So, they could be classified as B-cell-derived HLA II T-cell epitopes that can potentially activate CD4+ T-cells and B-cells.

### 3.3. Computer-Assisted Design of Polyepitope Vaccine Candidate against COVID-19

As explained in Materials and Methods, a multiepitope vaccine was designed containing the prioritized epitopes. However, due to overlap between the epitopes, it was necessary to select the best candidates amongst the overlapping peptides. A total of twelve epitopes (Table 1) were obtained after this filtration step, and they were ultimately used to design the multiepitope vaccine. Flexible linker sequences of GPGPG were used to join the epitopes with each other. Moreover, the CTB adjuvant sequence was added at the N-terminus of the epitopes and linked to them via a single EAAAK linker. Altogether, the multiepitope construct contained 284 amino acids, and the sequence is provided in plain format:

MTPQNITDLCAEYHNTQIHTLNDKIFSYTESLAGKREMAIITFKNGATFQVEVPGSQHIDSQKKAIERMKDTLRIAYLTEAKVEKLCVWNNKTPHAIAAISMANEAAAKFVFLVLLPLGPGPGSTQDLFLPFGPGPGWTAGAAAYYGPGPGYLQPRTFLLGPGPGYQPYRVVVLGPGPGQIITTDNTFGPGPGYFKIYSKHTGPGPGGINITRFQTLLALHRGPGPGFELLHAPATGPGPGYECDIPIGAGPGPGSIIAYTMSLGAENSVGPGPGFGAISSVLN.

### 3.4. Physiochemical, Antigenicity, Solubility, and Allergenicity Checks of Multiepitope

A physicochemical feature check of the multiepitope revealed that it was composed of 284 amino acids and had an 29832.13 amu molecular weight. Low molecular weight proteins (less than 110 kDa) are thought to be good candidates for developing vaccines [64]. The theoretical isoelectric point (pI) was predicted as 7.73, which lies in the normal range of pH conditions. The half-life of the polyepitope sequence was found to be 30 hours in mammalian reticulocytes (in vitro), more than 20 hours in yeast (in vivo), and more than 10 hours in *Escherichia coli* (in vivo). An instability index of 28.27 was computed. As the value is less than 40, this projects stability of our vaccine protein. The aliphatic index of 79.08 was calculated for our vaccine, which is a strong indication of thermostability at varying temperatures. Analysis revealed that the grand average of hydropathicity (GRAVY) is -0.081. Since it is a negative value, this suggests that the vaccine is hydrophilic in nature and has the ability to interact favourably with water molecules which is a desired property. Both ANTIGENPro and VaxiJen projected the antigenic nature of the vaccine construct, with scores of 0.764456 and 0.5098, respectively [28, 43]. The ccSol solubility predictor indicated that there was a 94% chance of the construct being soluble; another tool, SOLpro, corroborated this by confirming that the vaccine was soluble with a probability of 0.824941 [44, 45]. The allergenicity checks revealed that the protein sequence is not an allergen, so expectation is high that the vaccine will not cause side effects/toxicity inside the human body when administered. BLASTp against the human genome was conducted, and it was found that the sequence does not have any human homologue. Proteins that are homologues of humans may inadvertently cause autoimmunity due to cross-reactivity between the target and host proteins [65]. So, altogether physicochemical analysis of the polyepitope-based construct suggests its suitability for immunological applications.

### 3.5. Structural Modeling and Refinements of the Multiepitope Vaccine

**Figure 2:**
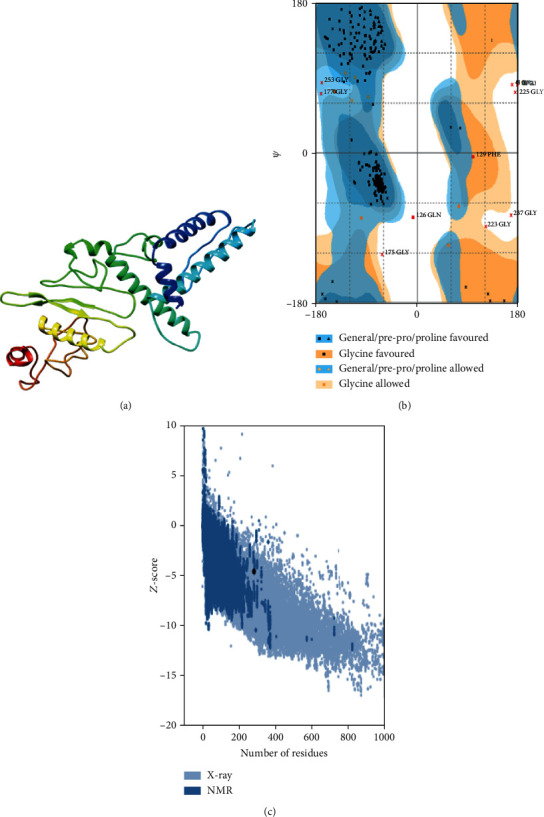
Structure-based assessments of the vaccine construct obtained after molecular modelling and refinements. (a) The three-dimensional refined model of the vaccine is visualized. (b) Ramachandran plot analysis of the structural model suggests high quality. (c) The ProSA *Z*-score of the best model is found to be good, i.e., -4.48, thus indicating near-native configuration.

The initial 3D structure of the multiepitope vaccine was obtained by the 3Dpro tool [43]. As this tool adopts a de novo method (structural templates are not used), it was considered appropriate especially due to the lack of good PDB templates to guide the structure prediction process. The model was then submitted to the GalaxyRefine2 server to undergo both refinements of local regions and overall improvement of the global structure [48]. Out of ten resulting models, one was chosen eventually based on the analysis of ProSA *Z*-scores (Figure 2(a)), Ramachandran plot, and ERRAT overall quality score. The selected model (model 1) had collectively more than 95% residues in the favorable and allowed regions and only 3.5% residues in the disallowed regions according to the RAMPAGE server (Figure 2(b)) [66]. The ProSA *Z*-score of this model was -4.48 which places it close to experimentally resolved structures of similar sizes, thus confirming near-native quality (Figure 2(c)). Analysis by the ERRAT server projected a quality score of 81.7204, suggesting that very few residues of the designed vaccine lie in erroneous regions [49]. Altogether, our strategy led to the best quality-refined 3D model of the vaccine construct.

### 3.6. Prediction of Discontinuous B-Cell Epitopes in the Structure of Polyepitope Vaccine

A total of 77 putative B-cell epitopes were identified using the DiscoTope server version 2.0 (Table S5), which indicates that more than 27% of the residues of the multiepitope formed discontinuous B-cell epitopes [51]. Results obtained after using Ellipro were even more promising [52]. It predicted a total of 148 residues (~52%) on the multiepitope structure that formed discontinuous B-cell epitopes (Table 2). The results obtained strongly support the notion that our vaccine targets will significantly activate specific humoral responses and thus lead to the induction of antibodies with the potential to limit viral infection.

### 3.7. Molecular Docking Analysis of Vaccine with TLR3

The active and passive interface residues of the multiepitope vaccine and the TLR3 structure were input into the HADDOCK server to conduct molecular docking [67]. This process led to the clustering of a total of 128 structures in 11 clusters, which in turn represented 64% of the water-refined models. Different graphs were obtained at this stage and are provided in Figure S1. The cluster-associated complexes showing the lowest HADDOCK scores in the range of −158.1 ± 13.2 were found to be the most reliable amongst all clusters. The statistical parameters and associated values (with standard deviation where applicable) are shown in Table 3. From this cluster, molecular refinements were applied to a single representative docked complex. The resulting 20 complexes fitted into one cluster, which represented all the models generated after water refinements at this step. The complexes within this cluster showed HADDOCK scores in the range of −274.4 ± 3.4. The statistical parameters and their associated values (with standard deviation where applicable) of this single cluster are shown (Table 4). Different graphs were also obtained at this stage (Figure S2).

**Figure 3:**
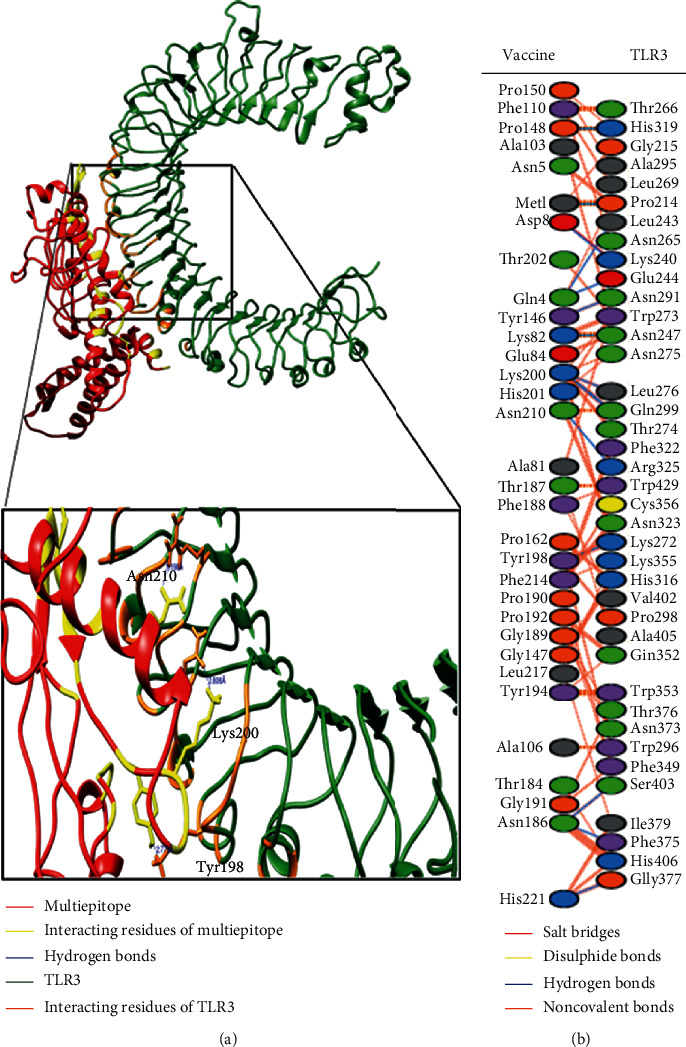
(a) Here, we show the interaction pose of a representative complex of the vaccine-TLR3 refined cluster. The designed vaccine is colored red while the TLR3 structure is colored dark green. The interacting residues are focused, and some interchain hydrogen bonds are highlighted. Residues Tyr198, Lys200, and Asn210 of the vaccine construct participated in hydrogen bond formation with some residues in the TLR3 structure. (b) Interacting residues as well as bonding interactions between the vaccine and TLR3 protein chains.

Molecular docking resulted in a good interaction pose between our vaccine and TLR3 chains (Figure 3(a)). A total of 32 residues of the vaccine were found to associate with a total of 39 TLR3 residues using PDBsum (Figure 3(b)) [68]. It was revealed that the interface area of the vaccine was 2012 (Å^2^) while that of TLR3 was 1905 (Å^2^).

Hydrogen bonds are enticing powers of the dipole-dipole and are formed when another electronegative atom absorbs a hydrogen atom bound to a strongly electronegative atom such as F, N, and O. The frequency of the hydrogen bond ranges from 4 kJ per mole to 50 kJ. In molecular recognition, hydrogen bonds are considered essential and have rigidity in achieving stable conformation. The vaccine was developed with 15 hydrogen bonds (chain A (vaccine)-chain B (TLR3); 1-214, 4-243, 4-244, 8-240, 82-247, 146-291, 148-319, 186-375, 186-403, 198-272, 200-276, 200-299 (2 H-bonds), 210-325, and 221-377). Upon detailed analysis of protein chains, it was found that Met1 forms a hydrogen bond with Pro214 at 2.85 Å. Similarly, Gln4-Leu243 forms a hydrogen bond at 3.21 Å distance, Gln4-Glu244 at 3.22 Å, Asp8-Lys240 at 2.62 Å, Lys82-Asn247 at 2.77 Å, Tyr146-Asn291 at 2.82 Å, Pro148-His319 at 2.77 Å, Asn186-Phe375 at 3.06 Å, Asn186-Ser403 at 3.16 Å, Tyr198-Lys272 at 2.78 Å, and Lys200-Leu276 at 2.72 Å. Lys200 formed two hydrogen bonds with Gln299 at distances of 2.99 Å and 2.81 Å, respectively. Finally, Asn210 formed one hydrogen bond with Gln325 at a distance of 3.20 Å, while His221 developed a hydrogen bond with Gly377 at a distance of 2.89 Å.

After careful observation, we found that most of the hydrogen bond distances between the interacting residues of the vaccine and TLR3 lie within 2–3 Å, thus indicating significantly high interactions [69]. So, these results support our argument that the multiepitope vaccine designed by us will associate with the TLR3 structure, and the appropriate downstream immune pathways will get activated thereafter.

### 3.8. Binding Affinity Assessment of Vaccine-TLR3 Complex

Gibbs free energy-based assessment of the refined vaccine-TLR3 complex was performed to assess the binding affinity. Analysis revealed that the *∆G* value of the complex was -19.5 kcal/mol. The negative *∆G* value indicates that the molecular association between our designed vaccine and TLR3 structure is thermodynamically possible and could definitely occur in nature. Also, the dissociation constant *K*_d_ value was calculated at 25°C and found to be 5.30*E*^−15^.

### 3.9. Normal Mode Analysis of Vaccine-TLR3 Complex

**Figure 4:**
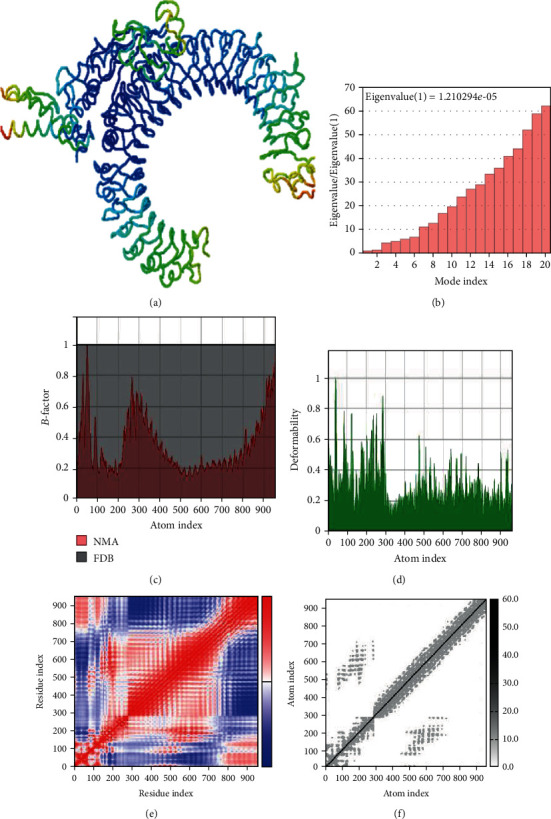
Normal mode analysis of the vaccine-TLR3 complex. The structural strength of refined protein-protein complex was analyzed as per generated graphs: mobility (a), eigenvalue vis-à-vis modes (b), *B*-factor vis-à-vis atoms (c), deformability vis-à-vis atoms (d), covariance vis-à-vis residue (e), and elastic network vis-à-vis atoms (f).

Normal mode analysis was carried out on the vaccine-TLR3 complex in order to study the overall maneuverability and compactness of proteins. In this procedure, the PDB complex internal coordinates were considered (Figure 4(a)). The residue direction is shown by arrows, while the line length is a visual representation of the extent of mobility in the three-dimensional model. It was revealed that the mobility of both proteins enabled their chains to face each other directly. The *B*-factor values were considered to be comparable to RMS (Figure 4(c)). The deformability potential of the complex was associated with the distortion of the protein residues, which is in turn indicated by chain hinges (Figure 4(d)). The eigenvalue computed for the vaccine-TLR3 complex was 1.210294*E*^−05^ (Figure 4(b)). The normal mode variance is known to exhibit an inverse relation with the eigenvalue [61, 70]. Also, the covariance matrix is a visual indication of coupling between residual pairs. This coupling could be associated with correlated, uncorrelated, or anticorrelated movements (Figure 4(e)). Finally, NMA generated an elastic network model (Figure 4(f)). This model identified the atom pairs joined together by springs. Each dot in the figure represented a spring between the corresponding atomic pairs, and coloring was done taking into account the stiffness level. The darker the grays are, the stiffer the springs are.

### 3.10. Reverse Translation and Codon Optimization of Vaccine Candidate

The optimized cDNA sequence of the candidate vaccine for the *E. coli* K12 strain was obtained after undergoing reverse translation followed by codon optimization. It was revealed that the Codon Adaptation Index (CAI) of cDNA was 0.876584. As this value is greater than 0.8, it was found to be convenient [71]. The GC contents of this sequence were computed and found to be 56.8075. As we know that GC contents should be within 30-70%, optimal results were achieved [72].

## 4. Discussion

The development of effective therapeutics and vaccines against COVID-19 is much needed to control this pandemic. Vaccination provides the most medically robust prevention against infectious disease eradication. Pursuing this route, the world has witnessed the end of the deadly smallpox disease, a medical feat that has indeed few parallels in science. Today, the most urgent global problem faced by humans is posed by the highly contagious SARS-CoV-2, the viral pathogen responsible for COVID-19. However, due to a lack of up-to-date immunological knowledge of the novel coronavirus species and the protective immune responses needed to clear the pathogen, vaccine development poses great challenges [73].

Recently, many studies have been performed to envision a COVID-19 vaccine. More than 135 vaccine candidates are undergoing preclinical testing, 15 vaccines have entered Phase I trials, and 11 vaccines are being subjected to Phase II trials [74]. A total of four vaccine candidates have even passed the Phase I and Phase II trials; thus, researchers will be studying large-scale efficacy in Phase III trials. Simian adenovirus-vectored vaccine ChAdOx1 nCoV-19 was developed by AstraZeneca and the University of Oxford [75]. The Phase III trials of this candidate vaccine are being conducted in Brazil and South Africa. Meanwhile, the Chinese company Sinopharm launched Phase III trials of an inactivated virus vaccine in July in the United Arab Emirates [76]. Another Chinese company, Sinovac Biotech, is conducting a Phase III trial in Brazil of an inactivated vaccine CoronaVac [77]. Interestingly, the protective TB vaccine BCG is also being subjected to a Phase III trial in Australia to assess its protective responses against COVID-19 [78]. Despite the current progress in vaccine development, it will take time to come up with an immunoprotective yet safe COVID-19 vaccine.

The spike protein is thought to be a crucial antigenic target for both SARS-CoV-2 and genomically similar SARS-CoV due to its high antigenicity and its functional role in terms of viral attachment and entry into human cells [79, 80]. It was recently reported that the SARS-CoV-2 spike protein contains two IgG immunodominant regions that were recognized by sera obtained from COVID-19 convalescent patients [81]. It was also found that specific targeting of these regions by antibodies led to considerable virus neutralization. Another study reported that recovered patients mounted a considerable CD4+ T cell response against the virus, with nearly 50% of it against the spike protein [15]. Considering the available evidence, it could be argued that the spike protein is a good target for designing and developing a vaccine. Using a variety of computational tools, we selected high-ranked promiscuous epitopes.

The main aim of the present study was to propose a multiepitope vaccine against COVID-19 using computer-aided analysis. The rationale of including a molecular adjuvant in the multiepitope construct was that epitope-based peptide vaccines, although safe and highly specific, are normally poor immunogens [82]. So, we also incorporated the N-terminus molecular adjuvant Cholera Toxin *β* subunit (CTB) which is the nontoxic part of the cholera toxin [39]. CTB is safe for use by humans, tolerated well by immunocompromised persons, and could also activate specific CD4+ T cell responses [83, 84]. Further, the prioritized epitopes were joined together using flexible GPGPG linkers for effective separation while the EAAAK linker joined the CTB adjuvant with the first epitope.

A high-quality near-native vaccine structure was obtained after structural modeling and molecular refinements. This was confirmed using the ProSA web server and its *Z*-score [50]. Quality assessment by this server confirmed that the vaccine's 3D structure lies in the vicinity of the experimentally resolved PDB structures of similar sizes. Ramachandran plot analysis revealed that more than 95% of the residues lie in the favoured and allowed regions [66]. Only a minimum number of residues lie in outlier regions. Furthermore, structural evaluations using the ERRAT server clearly indicated that erroneous regions were very few in the refined structural model of the multiepitope vaccine [49].

It is known that the majority of B-cell epitopes (~90%) are not linear, i.e., they are not present in the primary structure [85]. After protein folding, some residues end up close to each other spatially in the three-dimensional antigen structure and are recognized by B-cells [86]. So, we predicted the discontinuous B-cell epitopes in the refined structural model of the multiepitope vaccine. What we found of immense importance was that our vaccine contained a lot of discontinuous B-cell epitopes. More than 27% of the multiepitope residues were projected to be part of conformational B-cell epitopes using the DiscoTope server 2.0 [51], whereas Ellipro predicted that almost 52% of the residues of the vaccine formed conformational B-cell epitopes [52]. Altogether, our analyses support the proposition that our designed vaccine construct will activate humoral immune responses and lead to the successful induction of pathogen-specific antibodies.

To check the effectiveness of our multiepitope vaccine, we conducted molecular docking of the vaccine structure with the TLR3 structure. This was necessary as TLR3 acts as a sensor of viral infection and activates downstream innate immunity-related signaling pathways [23]. Studies on SARS-CoV and MERS indicate the role of TLR3 in the development of a protective response against coronaviruses [24, 25]. Moreover, TLR3-mediated induction of type I interferon conferred protection against murine coronavirus infection of macrophages [87].

The guru level interface in HADDOCK provided good results in terms of stability as well as flexibility of protein-protein docked clusters [88]. The HADDOCK server employs an integrative approach of the biophysical interaction and/or biochemical data. Moreover, ab initio PPIs obtain result-oriented and noteworthy docking results [89]. The HADDOCK protocol is highly adaptive in terms of conducting solvated docking, specifying flexible protein regions, and providing the option of considering modified amino acids. After molecular docking of the designed vaccine with TLR3, refinements were applied on top of the docked complex to further portray the PPIs. As expected, it was observed that the HADDOCK score improved from −158.1 ± 13.2 to −274.4 ± 3.4 after applying molecular refinements. Lower HADDOCK scores, especially negative values, underscore greater interaction potential between the proteins. Analyses of RMSD indicated that high-quality structures did not show a lot of structural deviations from near-native states which is a desired outcome. A Gibbs free energy-based assessment of the vaccine-TLR3 complex predicted energetically favorable interaction. The stability of the complex was also confirmed through NMA analysis; hence, a large amount of force is needed to separate the protein chains from each other. Moreover, the vaccine could be easily overexpressed in the *E. coli* K12 strain as we showed after codon optimization of the candidate vaccine.

One limitation of the designed study is the lack of follow-up wet laboratory studies to validate whether the multiepitope vaccine will provide strong protective immune responses in humans or not. The epitopes incorporated in this vaccine have been strictly identified as per computer-aided analysis. Currently, we cannot find any indication of their experimental testing concerning COVID-19 through the IEDB resource that has the repository of experimentally characterized epitopes associated with both T-cells and B-cells. After scanning the literature, we found some published studies concerning epitope prediction in the spike protein [18–20]. After manual comparison of their predictions with our study, we found that none of the epitopes proposed in our study have been projected earlier, which indicates novelty. It is essential to conduct further in vitro and in vivo studies to test the safety and immunogenicity of the proposed epitopes and the polyepitope vaccine.

## 5. Conclusions

Effective vaccines are urgently required worldwide to contain the spread of the COVID-19 pandemic and save human lives. In this study, we carried out a computer-assisted analysis on the spike protein of SARS-CoV-2 to identify highly promising epitopes to guide the development of a safe universal vaccine. These prioritized epitopes were then fused with each other and an adjuvant to form a polyepitope subunit vaccine using computational biology approaches. Three-dimensional structural modeling of the vaccine was carried out and subjected to docking studies with Toll-like Receptor 3 to assess its immunological effectiveness. Our results indicated a strong interaction between our vaccine and the innate receptor, strengthening the proposition that it will lead to the induction of TLR-mediated downstream immune pathways. The present study is entirely *in silico* work and should be validated by future studies before conducting trials in humans.

## Figures and Tables

**Table 1:** List of prioritized epitopes and their putative properties as well as their conservation in 296 spike protein sequences.

Epitopes	No. of HLA supertypes/alleles	HLA supertypes/alleles	Antigenicity score	Conservation level
FVFLVLLPL	4	A2, A26, B8, B62	0.8601	284/296
STQDLFLPF	4	A1, A24, A26, B62	0.6619	290/296
WTAGAAAYY	4	A1, A26, B58, B62	0.6306	292/296
YLQPRTFLL	4	A2, B8, B39, B62	0.4532	294/296
YQPYRVVVL	5	A2, A24, B8, B39, B62	0.5964	296/296
QIITTDNTF	4	A24, A26, B58, B62	0.4253	292/296
YFKIYSKHT	6	DRB1∗0101, DRB1∗0401, DRB1∗0701, DRB1∗0801, DRB1∗1101, DRB1∗1501	0.9056	296/296
GINITRFQTLLALHR	5	DRB1∗0101, DRB1∗0401, DRB1∗0801, DRB1∗1101, DRB1∗1501	0.5582	292/296
FELLHAPAT	4	DRB1∗0101, DRB1∗0401, DRB1∗1101, DRB1∗1501	0.5409	290/296
YECDIPIGA	4	DRB1∗0101, DRB1∗0301, DRB1∗0401, DRB1∗0801	0.6385	294/296
SIIAYTMSLGAENSV	3	DRB1∗0101, DRB1∗0401, DRB1∗0701	0.5691	291/296
FGAISSVLN	5	DRB1∗0101, DRB1∗0401, DRB1∗0701, DRB1∗0801, DRB1∗1501	0.5435	296/296

**Table 2:** Ellipro results showing discontinuous B-cell epitopes.

Sr. no.	Residues	Number of residues	Score
1	T183-G191	9	0.76
2	P226, P234-N284	52	0.69
3	M1-I6, D8, L9, A11-N15, Q17, I18, T20, L21, K24, T125-D127	21	0.635
4	L86-H95	10	0.845
5	I25-E37, N45, G46-K64	33	0.797
6	G135, G137, W138, A140-G147, Q154-R156, F158-Y166	23	0.587

**Table 3:** Statistics of the highest-scored vaccine-TLR3 docked cluster. A negative value of the HADDOCK score projects good protein-protein interaction potential.

Parameters	Value
HADDOCK score	−158.1 ± 13.2
Size of cluster	27
RMSD from structure with the lowest energy	4.2 ± 0.1
Van der Waals energy	−137.0 ± 5.5
Electrostatic energy	−282.2 ± 17.9
Desolvation energy	−58.9 ± 3.5
Restraint violation energy	942.5 ± 150.18
Buried surface area	4113.6 ± 125.2
*Z*-score	0

**Table 4:** Statistics of a single cluster obtained after applying refinements on top-ranked docked vaccine-TLR3 structure.

Parameters	Value
HADDOCK score	−274.4 ± 3.4
Size of cluster	20
RMSD from structure with the lowest energy	0.3 ± 0.2
Van der Waals energy	−145.3 ± 3.0
Electrostatic energy	−291.2 ± 8.6
Desolvation energy	−70.8 ± 7.3
Restraint violation energy	0.0 ± 0.00
Buried surface area	4040.4 ± 39.8
*Z*-score	0

## Data Availability

The relevant data used to support the findings of this study are included within the article.

## Supplementary Materials

Supplementary Materials Table S1: epitope filtration steps to finalize HLA I epitopes in the SARS-CoV-2 spike glycoprotein sequence. Table S2: epitope filtration steps to finalize HLA II epitopes in the SARS-CoV-2 spike glycoprotein sequence after PREDIVAC and other tools. Table S3: epitope filtration steps to finalize HLA II epitopes in the SARS-CoV-2 spike glycoprotein sequence after NetMHCIIpan and other tools. Table S4: predicted B-cell linear epitopes in the SARS-CoV-2 glycoprotein with probability values. Table S5: predicted discontinuous B-cell epitopes in the multiepitope vaccine according to the DiscoTope server. Figure S1: graphs obtained after molecular docking between vaccine and TLR3 structures. Figure S2: graphs obtained after applying refinements on the top vaccine-TLR3 docked structure.
