# Supplementary material for: Multiepitope Subunit Vaccine Design against COVID-19 Based on the Spike Protein of SARS-CoV-2: An In Silico Analysis
Source: J Immunol Res. 2020 Nov 19;2020:8893483. doi: 10.1155/2020/8893483 (PMC7678744; doi:10.1155/2020/8893483)
Supplement: Supplementary Materials — Table S1: epitope filtration steps to finalize HLA I epitopes in the SARS-CoV-2 spike glycoprotein sequence. Table S2: epitope filtration steps to finalize HLA II epitopes in the SARS-CoV-2 spike glycoprotein sequence after PREDIVAC and other tools. Table S3: epitope filtration steps to finalize HLA II epitopes in the SARS-CoV-2 spike glycoprotein sequence after NetMHCIIpan and other tools. Table S4: predicted B-cell linear epitopes in the SARS-CoV-2 glycoprotein with probability values. Table S5: predicted discontinuous B-cell epitopes in the multiepitope vaccine according to the DiscoTope server. Figure S1: graphs obtained after molecular docking between vaccine and TLR3 structures. Figure S2: graphs obtained after applying refinements on the top vaccine-TLR3 docked structure. [file 8893483.f1.zip › Supplementary figure 1.pdf]

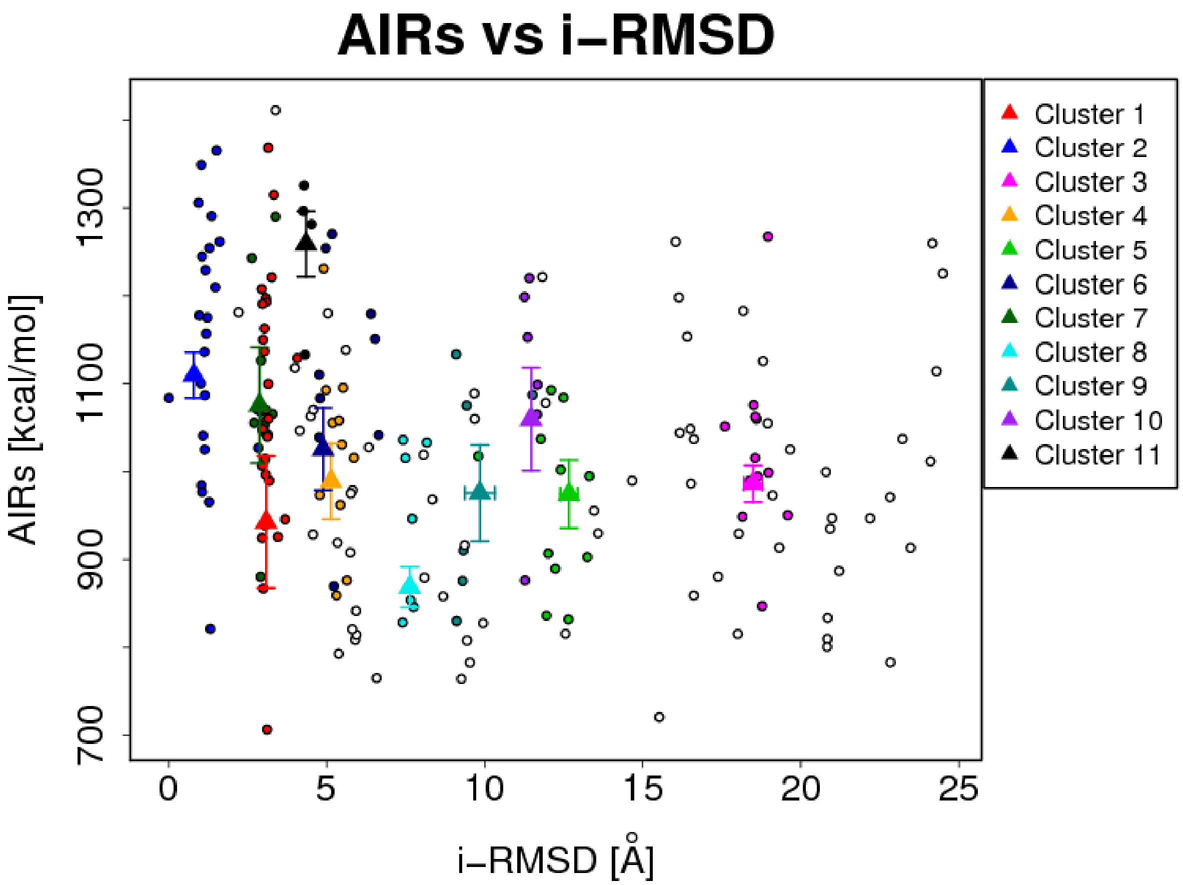
AIRs vs i−RMSD
AIRs [kcal/mol]
i−RMSD [Å]
700
900
1100
1300
0
5
10
15
20
25
Cluster 1
Cluster 2
Cluster 3
Cluster 4
Cluster 5
Cluster 6
Cluster 7
Cluster 8
Cluster 9
Cluster 10
Cluster 11

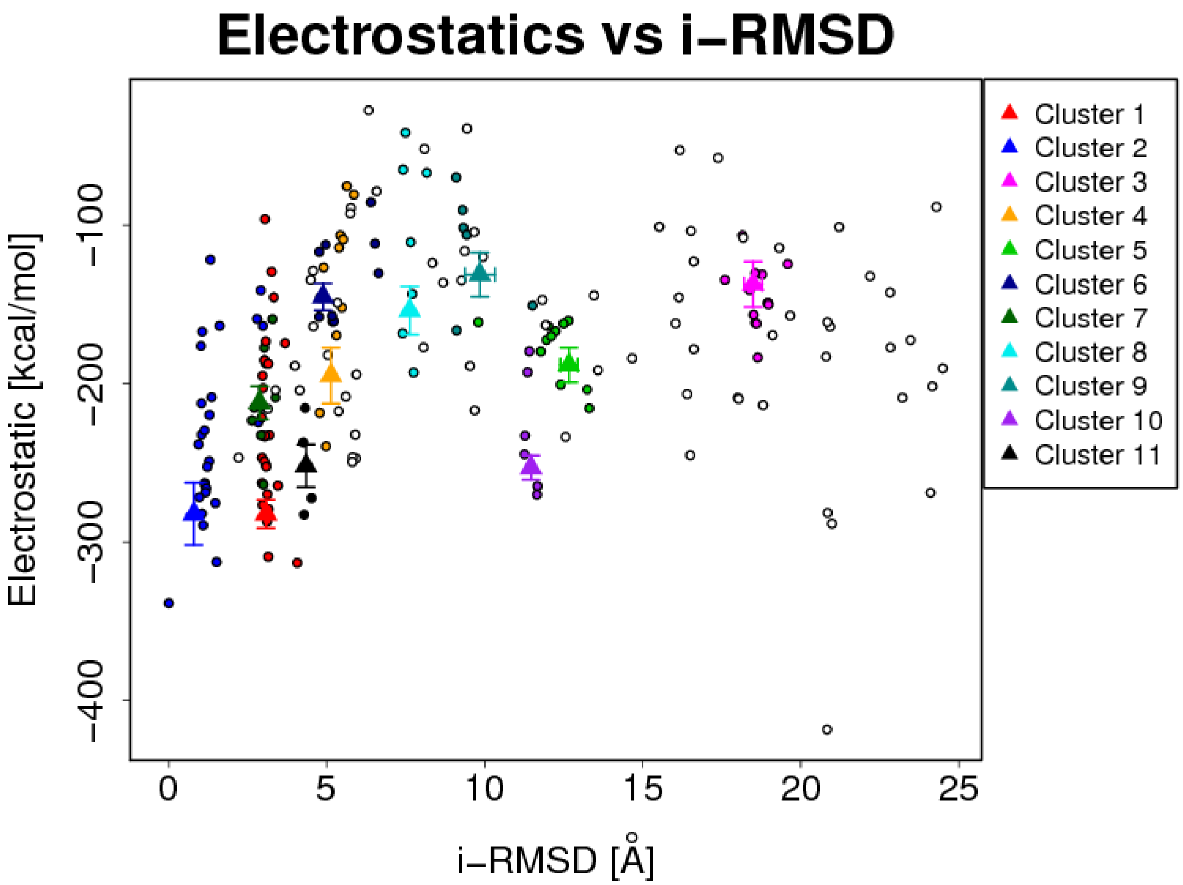
Electrostatics vs i−RMSD
Electrostatic [kcal/mol]
−100
−200
−300
−400
0
5
10
15
20
25
i−RMSD [Å]
Cluster 1
Cluster 2
Cluster 3
Cluster 4
Cluster 5
Cluster 6
Cluster 7
Cluster 8
Cluster 9
Cluster 10
Cluster 11

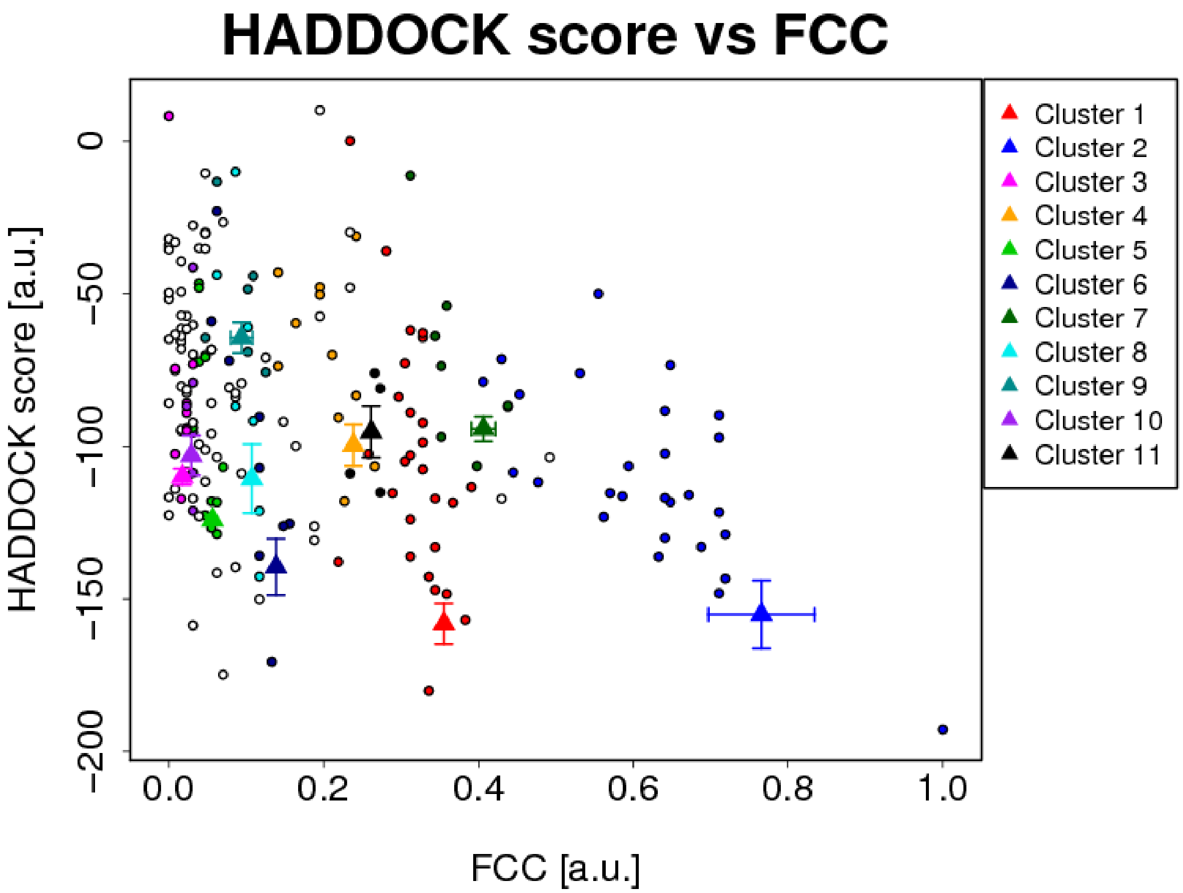
HADDOCK score vs FCC
HADDOCK score [a.u.]
FCC [a.u.]
0
−50
−100
−150
−200
0.0
0.2
0.4
0.6
0.8
1.0
Cluster 1
Cluster 2
Cluster 3
Cluster 4
Cluster 5
Cluster 6
Cluster 7
Cluster 8
Cluster 9
Cluster 10
Cluster 11

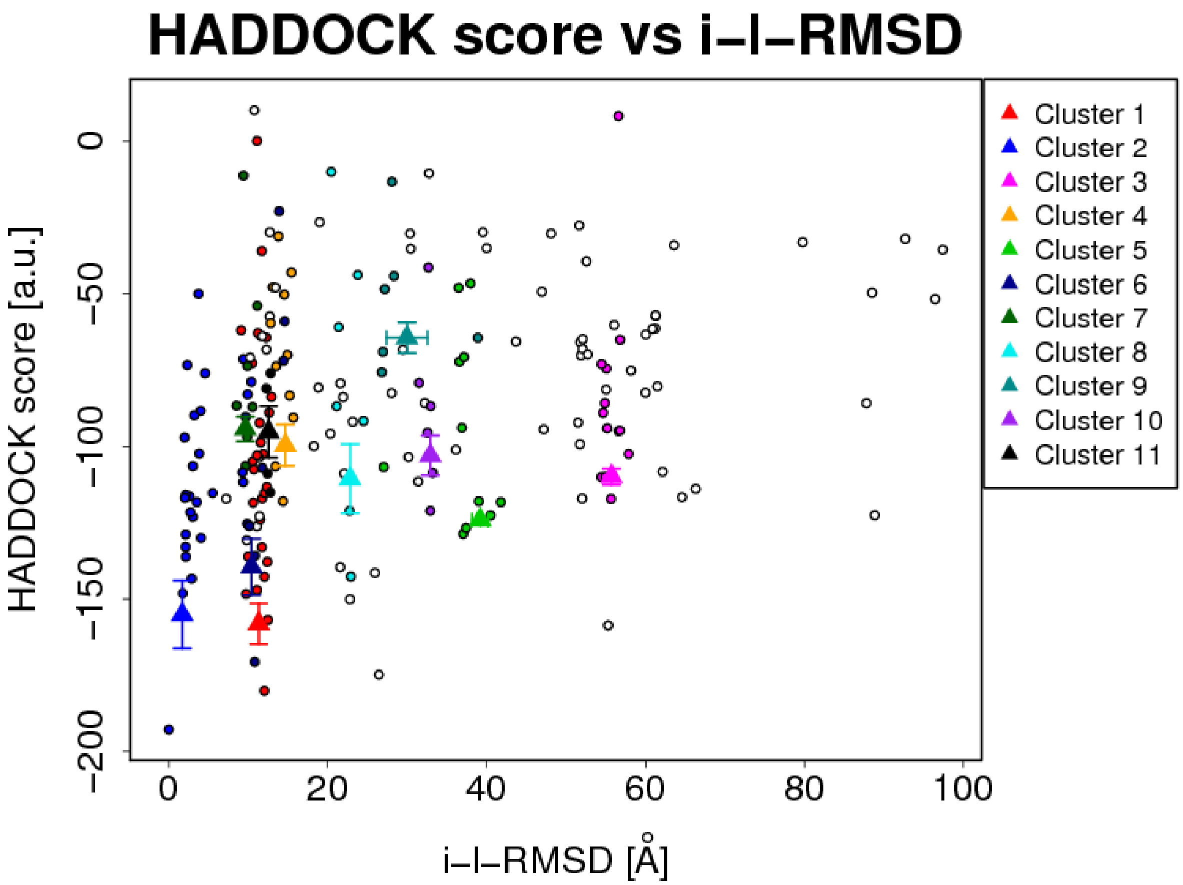

HADDOCK score vs i–l–RMSD
HADDOCK score [a.u.]
0
−50
−100
−150
−200
0
20
40
60
80
100
i–l–RMSD [Å]
Cluster 1
Cluster 2
Cluster 3
Cluster 4
Cluster 5
Cluster 6
Cluster 7
Cluster 8
Cluster 9
Cluster 10
Cluster 11

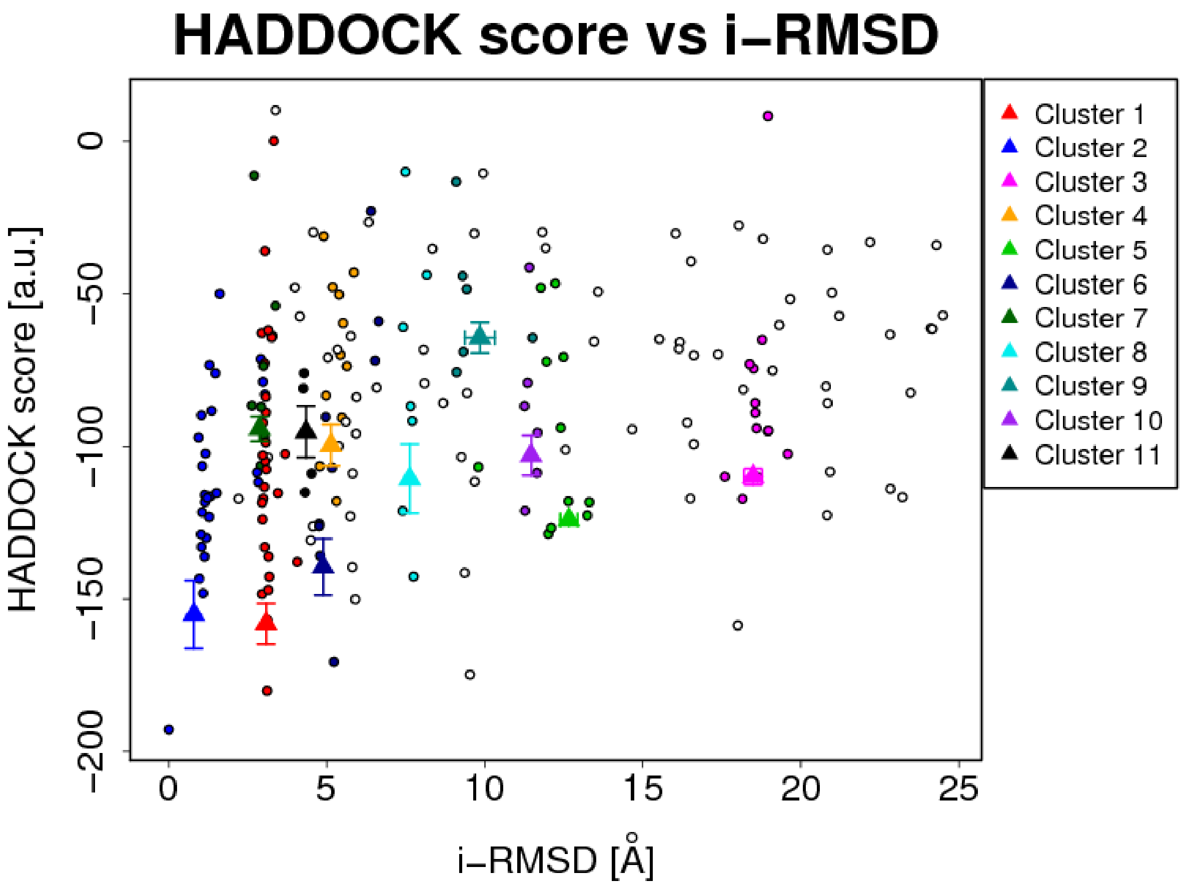

HADDOCK score vs i−RMSD
HADDOCK score [a.u.]
i−RMSD [Å]
0
−50
−100
−150
−200
0
5
10
15
20
25
Cluster 1
Cluster 2
Cluster 3
Cluster 4
Cluster 5
Cluster 6
Cluster 7
Cluster 8
Cluster 9
Cluster 10
Cluster 11

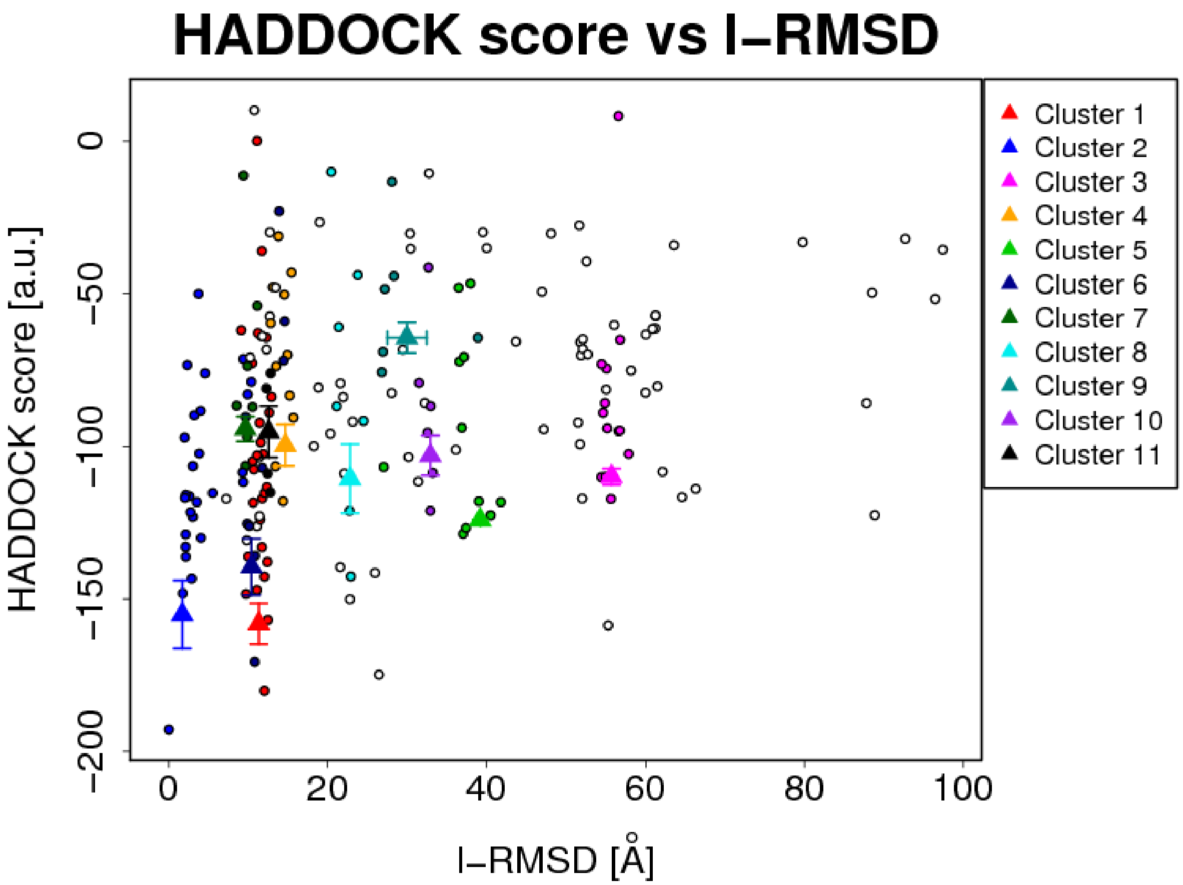
HADDOCK score vs I−RMSD
HADDOCK score [a.u.]
0
−50
−100
−150
−200
0
20
40
60
80
100
I−RMSD [Å]
Cluster 1
Cluster 2
Cluster 3
Cluster 4
Cluster 5
Cluster 6
Cluster 7
Cluster 8
Cluster 9
Cluster 10
Cluster 11

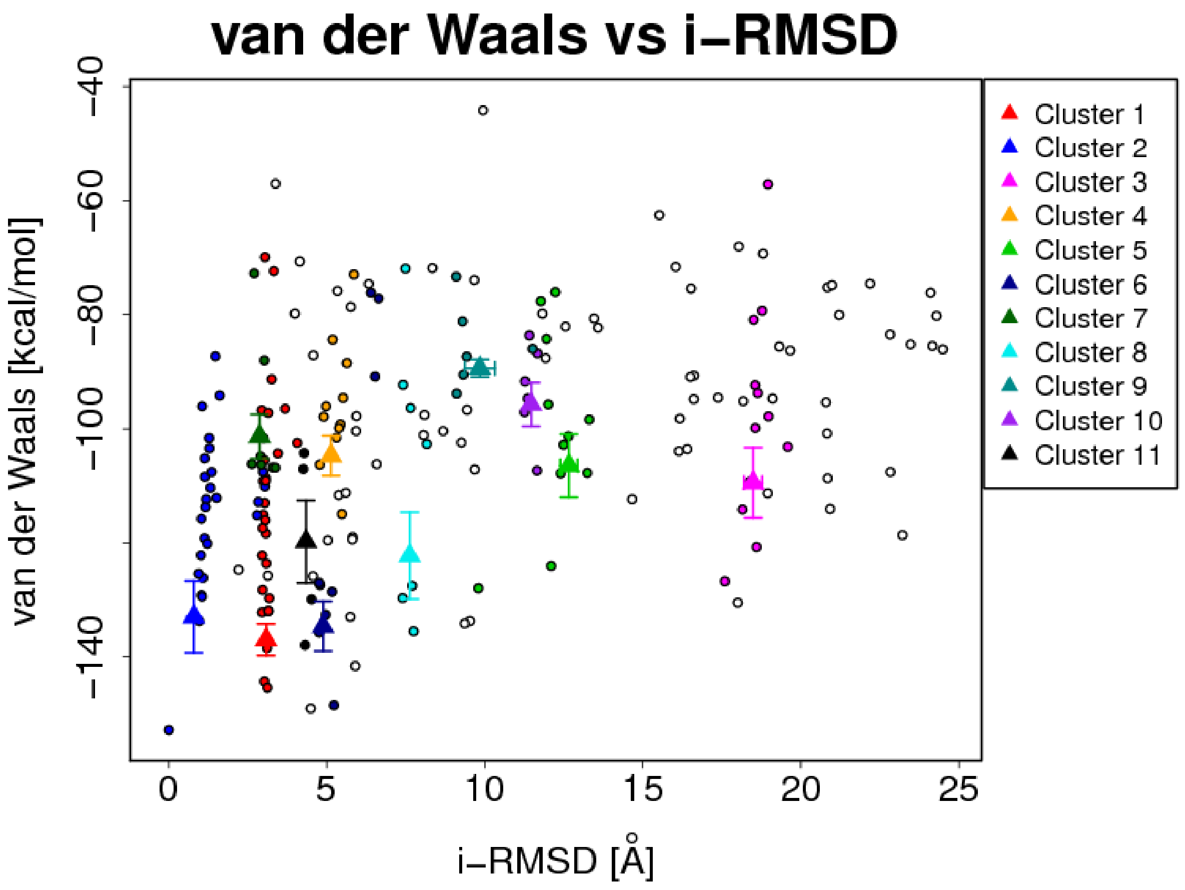

van der Waals vs i−RMSD
van der Waals [kcal/mol]
−40
−60
−80
−100
−140
i−RMSD [Å]
0
5
10
15
20
25
Cluster 1
Cluster 2
Cluster 3
Cluster 4
Cluster 5
Cluster 6
Cluster 7
Cluster 8
Cluster 9
Cluster 10
Cluster 11
