# Supplementary material for: Multiepitope Subunit Vaccine Design against COVID-19 Based on the Spike Protein of SARS-CoV-2: An In Silico Analysis
Source: J Immunol Res. 2020 Nov 19;2020:8893483. doi: 10.1155/2020/8893483 (PMC7678744; doi:10.1155/2020/8893483)
Supplement: Supplementary Materials — Table S1: epitope filtration steps to finalize HLA I epitopes in the SARS-CoV-2 spike glycoprotein sequence. Table S2: epitope filtration steps to finalize HLA II epitopes in the SARS-CoV-2 spike glycoprotein sequence after PREDIVAC and other tools. Table S3: epitope filtration steps to finalize HLA II epitopes in the SARS-CoV-2 spike glycoprotein sequence after NetMHCIIpan and other tools. Table S4: predicted B-cell linear epitopes in the SARS-CoV-2 glycoprotein with probability values. Table S5: predicted discontinuous B-cell epitopes in the multiepitope vaccine according to the DiscoTope server. Figure S1: graphs obtained after molecular docking between vaccine and TLR3 structures. Figure S2: graphs obtained after applying refinements on the top vaccine-TLR3 docked structure. [file 8893483.f1.zip › Supplementary figure 2.pdf]

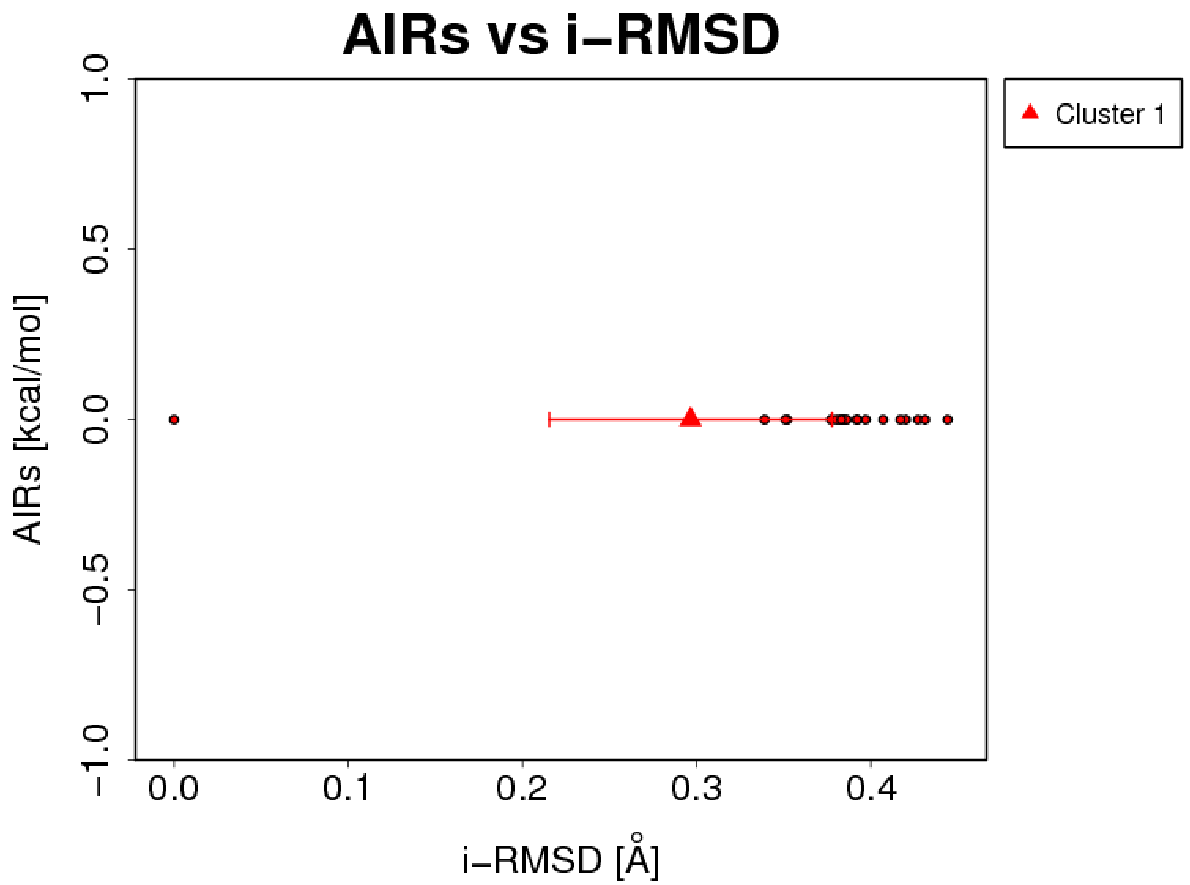
AIRs vs i−RMSD
AIRs [kcal/mol]
1.0
0.5
0.0
−0.5
−1.0
0.0
0.1
0.2
0.3
0.4
i−RMSD [Å]
Cluster 1

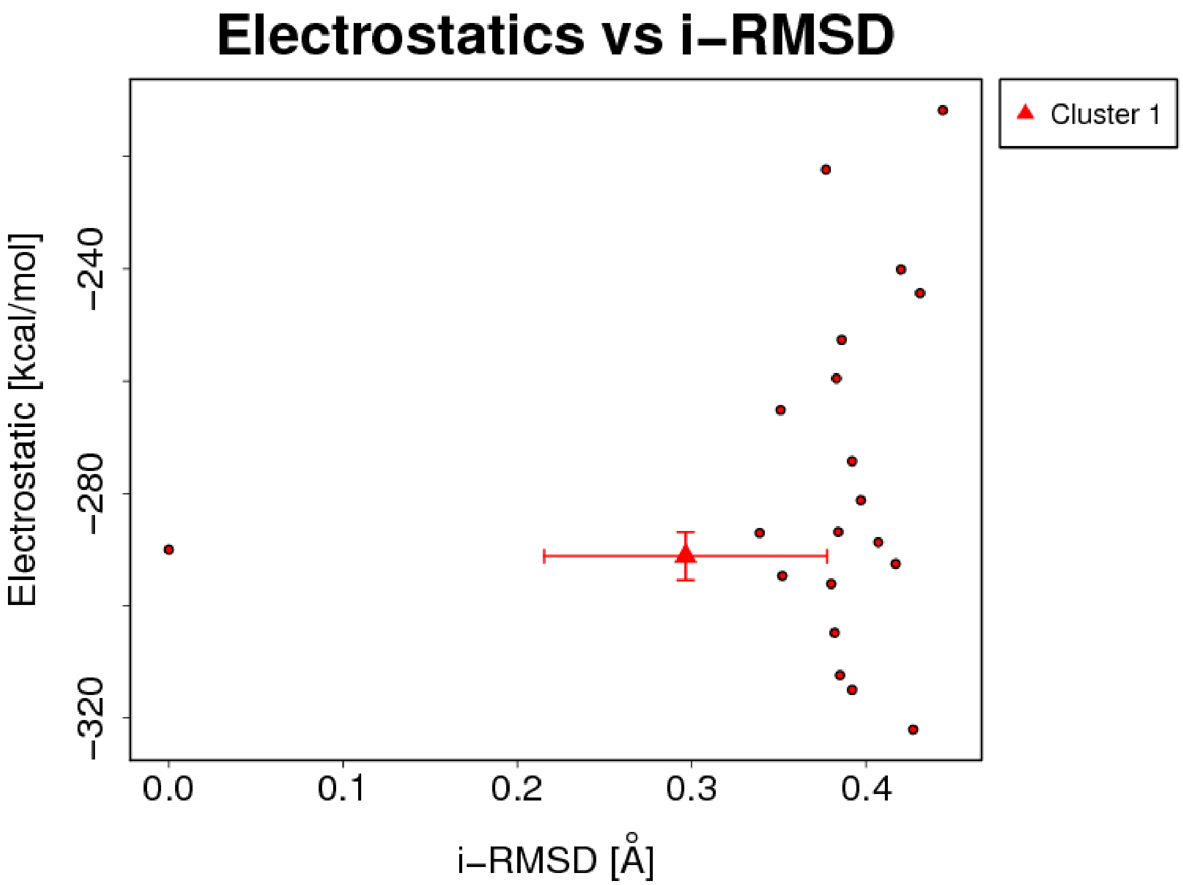

Electrostatics vs i−RMSD
Cluster 1
Electrostatic [kcal/mol]
−240
−280
−320
0.0
0.1
0.2
0.3
0.4
i−RMSD [Å]

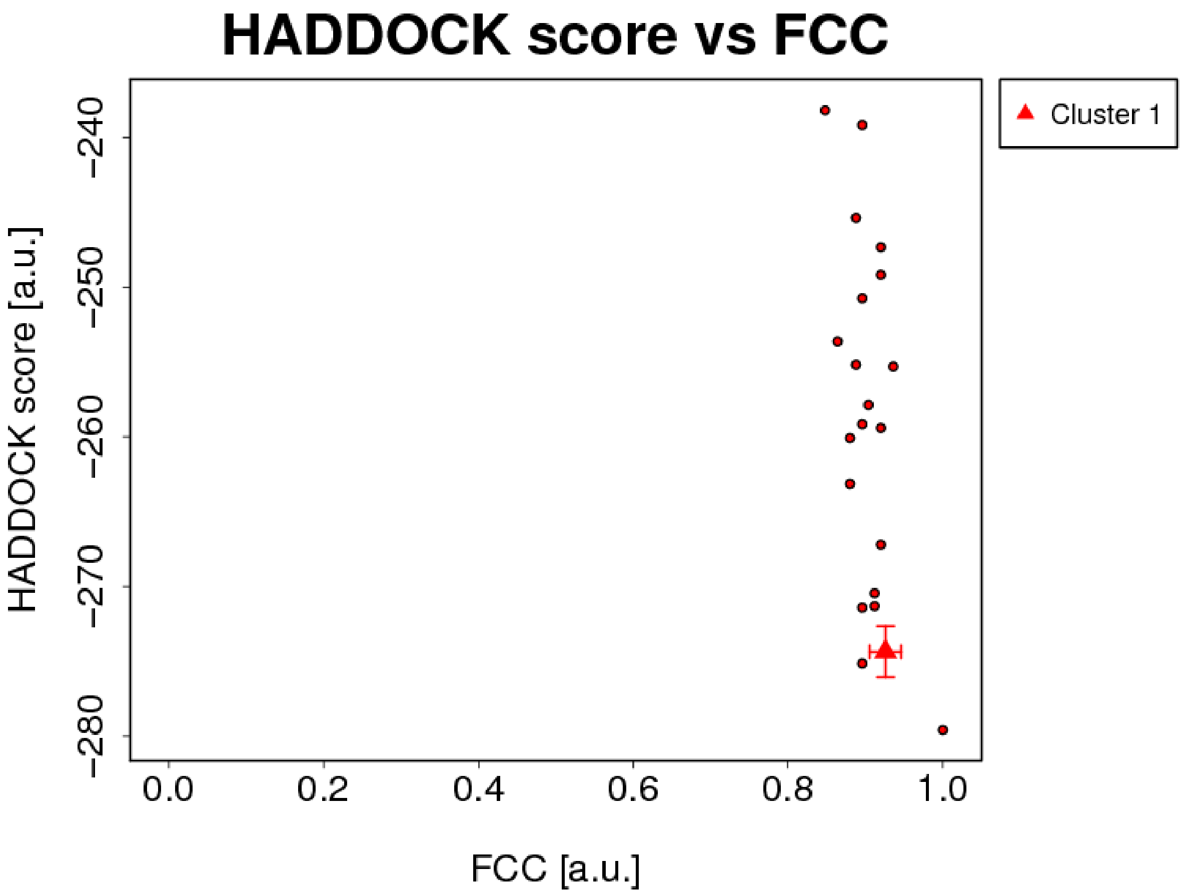
HADDOCK score vs FCC
Cluster 1
HADDOCK score [a.u.]
−240
−250
−260
−270
−280
0.0
0.2
0.4
0.6
0.8
1.0
FCC [a.u.]

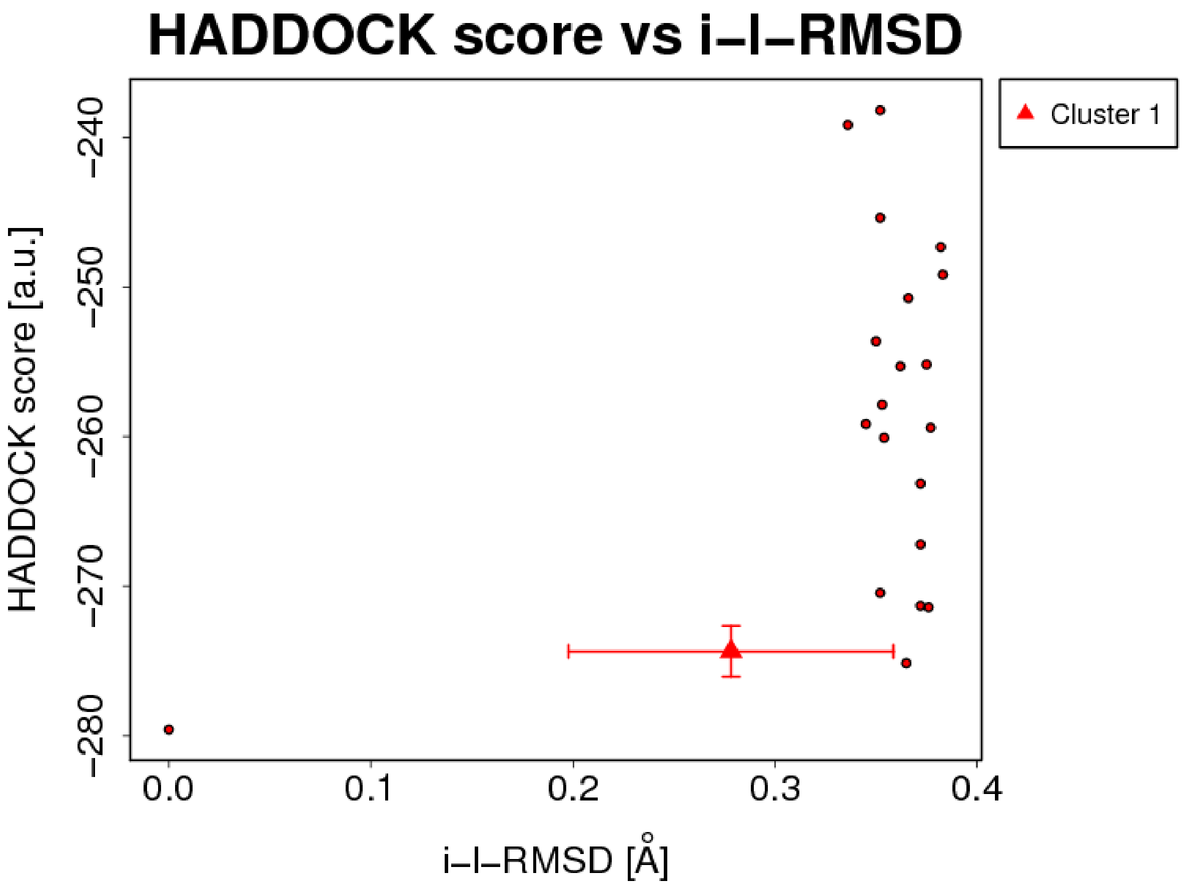
HADDOCK score vs i–l–RMSD
Cluster 1
HADDOCK score [a.u.]
−240
−250
−260
−270
−280
0.0
0.1
0.2
0.3
0.4
i–l–RMSD [Å]

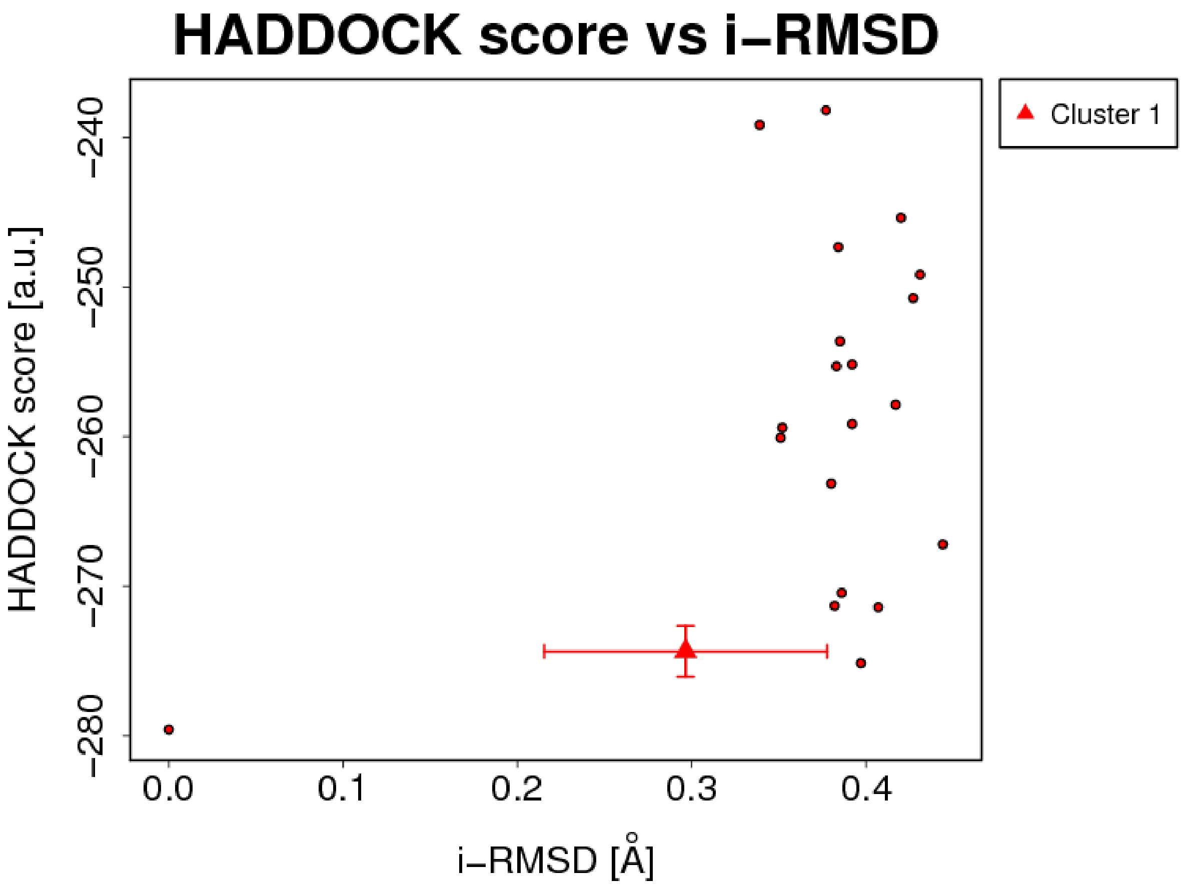

HADDOCK score vs i−RMSD
HADDOCK score [a.u.]
−240
−250
−260
−270
−280
0.0
0.1
0.2
0.3
0.4
i−RMSD [Å]
Cluster 1

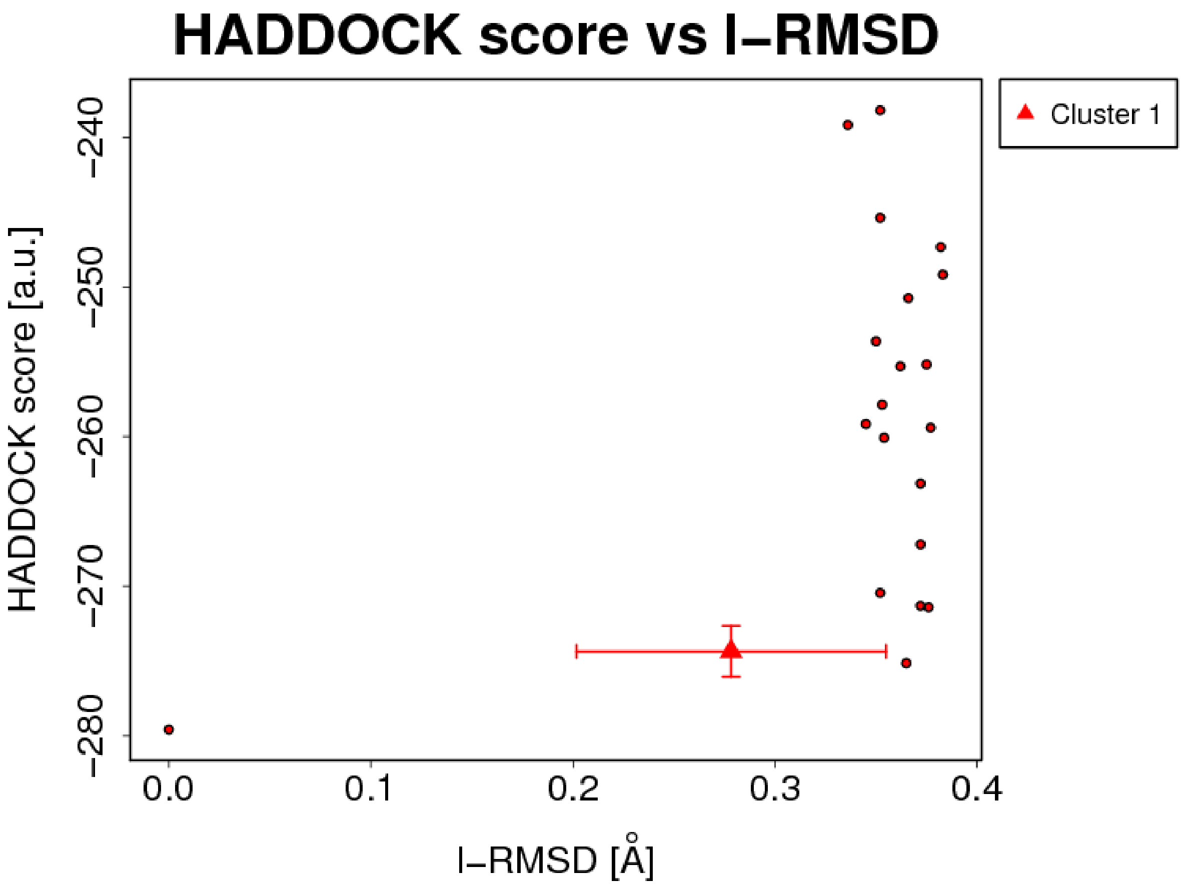
HADDOCK score vs I−RMSD
Cluster 1
HADDOCK score [a.u.]
−240
−250
−260
−270
−280
0.0
0.1
0.2
0.3
0.4
I−RMSD [Å]

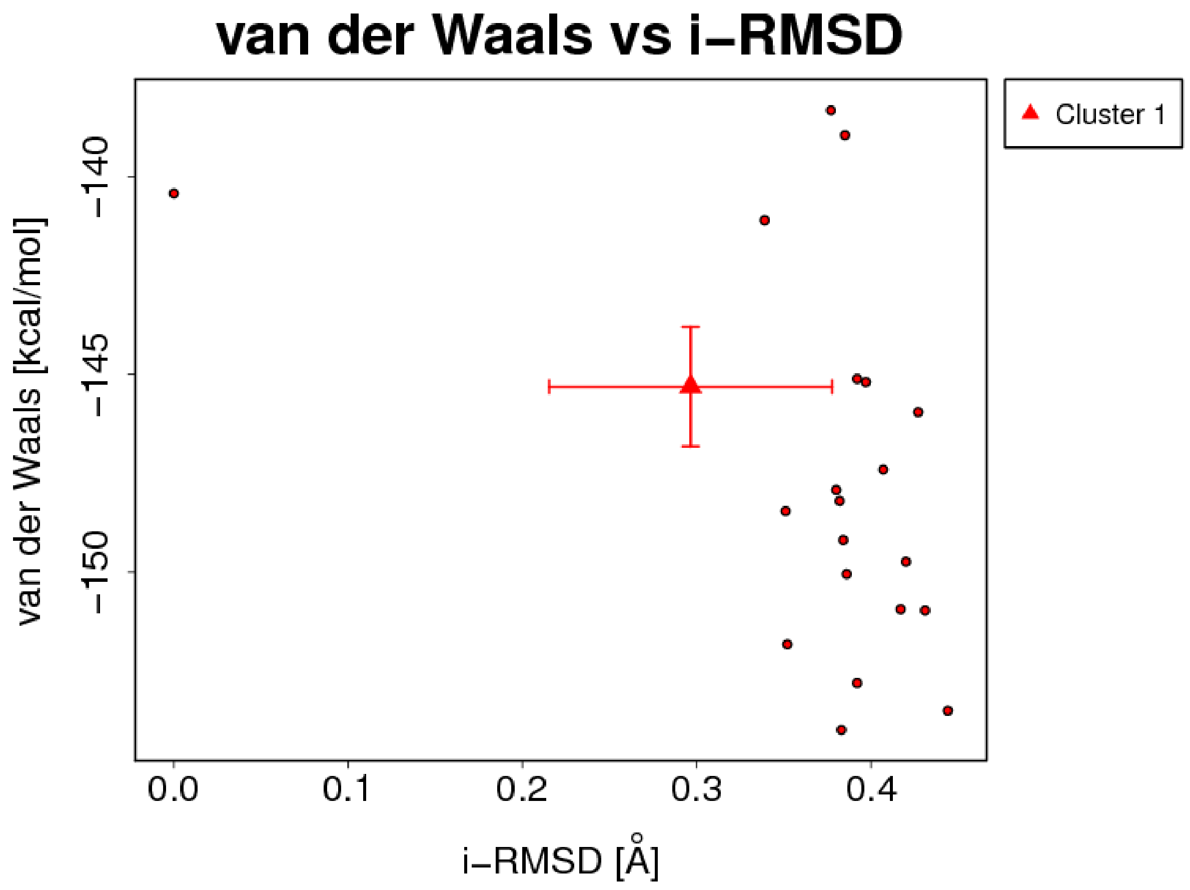
van der Waals vs i−RMSD
Cluster 1
van der Waals [kcal/mol]
−140
−145
−150
0.0
0.1
0.2
0.3
0.4
i−RMSD [Å]
