# Supplementary material for: Multiepitope Subunit Vaccine Design against COVID-19 Based on the Spike Protein of SARS-CoV-2: An In Silico Analysis
Source: J Immunol Res. 2020 Nov 19;2020:8893483. doi: 10.1155/2020/8893483 (PMC7678744; doi:10.1155/2020/8893483)
Supplement: Supplementary Materials — Table S1: epitope filtration steps to finalize HLA I epitopes in the SARS-CoV-2 spike glycoprotein sequence. Table S2: epitope filtration steps to finalize HLA II epitopes in the SARS-CoV-2 spike glycoprotein sequence after PREDIVAC and other tools. Table S3: epitope filtration steps to finalize HLA II epitopes in the SARS-CoV-2 spike glycoprotein sequence after NetMHCIIpan and other tools. Table S4: predicted B-cell linear epitopes in the SARS-CoV-2 glycoprotein with probability values. Table S5: predicted discontinuous B-cell epitopes in the multiepitope vaccine according to the DiscoTope server. Figure S1: graphs obtained after molecular docking between vaccine and TLR3 structures. Figure S2: graphs obtained after applying refinements on the top vaccine-TLR3 docked structure. [file 8893483.f1.zip › Supplementary Table 1.docx]

Supplementary Table 1

| **Epitopes** | **Antigenicity score** |
| --- | --- |
| QIITTDNTF | 0.4253 |
| WTAGAAAYY | 0.6306 |
| YQPYRVVVL | 0.5964 |
| YLQPRTFLL | 0.4532 |
| STQDLFLPF | 0.6619 |
| RVVVLSFEL | 1.1918 |
| FVFLVLLPL | 0.6731 |
