# Supplementary material for: Multiepitope Subunit Vaccine Design against COVID-19 Based on the Spike Protein of SARS-CoV-2: An In Silico Analysis
Source: J Immunol Res. 2020 Nov 19;2020:8893483. doi: 10.1155/2020/8893483 (PMC7678744; doi:10.1155/2020/8893483)
Supplement: Supplementary Materials — Table S1: epitope filtration steps to finalize HLA I epitopes in the SARS-CoV-2 spike glycoprotein sequence. Table S2: epitope filtration steps to finalize HLA II epitopes in the SARS-CoV-2 spike glycoprotein sequence after PREDIVAC and other tools. Table S3: epitope filtration steps to finalize HLA II epitopes in the SARS-CoV-2 spike glycoprotein sequence after NetMHCIIpan and other tools. Table S4: predicted B-cell linear epitopes in the SARS-CoV-2 glycoprotein with probability values. Table S5: predicted discontinuous B-cell epitopes in the multiepitope vaccine according to the DiscoTope server. Figure S1: graphs obtained after molecular docking between vaccine and TLR3 structures. Figure S2: graphs obtained after applying refinements on the top vaccine-TLR3 docked structure. [file 8893483.f1.zip › Supplementary Table 2.docx]

Supplementary Table 2

| **Epitope** | **Antigenicity score (VaxiJen)** | **IC50 value (DRB1*0101)** |
| --- | --- | --- |
| FELLHAPAT | 0.5409 | 18.11 |
| FLVLLPLVS | 0.4266 | 0.48 |
| YFKIYSKHT | 0.9056 | 39.36 |
| FGAISSVLN | 0.5435 | 368.13 |
| YECDIPIGA | 0.6385 | 14 |
