# Supplementary material for: Multiepitope Subunit Vaccine Design against COVID-19 Based on the Spike Protein of SARS-CoV-2: An In Silico Analysis
Source: J Immunol Res. 2020 Nov 19;2020:8893483. doi: 10.1155/2020/8893483 (PMC7678744; doi:10.1155/2020/8893483)
Supplement: Supplementary Materials — Table S1: epitope filtration steps to finalize HLA I epitopes in the SARS-CoV-2 spike glycoprotein sequence. Table S2: epitope filtration steps to finalize HLA II epitopes in the SARS-CoV-2 spike glycoprotein sequence after PREDIVAC and other tools. Table S3: epitope filtration steps to finalize HLA II epitopes in the SARS-CoV-2 spike glycoprotein sequence after NetMHCIIpan and other tools. Table S4: predicted B-cell linear epitopes in the SARS-CoV-2 glycoprotein with probability values. Table S5: predicted discontinuous B-cell epitopes in the multiepitope vaccine according to the DiscoTope server. Figure S1: graphs obtained after molecular docking between vaccine and TLR3 structures. Figure S2: graphs obtained after applying refinements on the top vaccine-TLR3 docked structure. [file 8893483.f1.zip › Supplementary Table 3.docx]

Supplementary Table 3

| **Epitopes** | **Number of HLA II alleles** | **HLA II alleles** | | | | | |
| --- | --- | --- | --- | --- | --- | --- | --- |
| TRFQTLLALHRSYLT | 6 | DRB1*0101 | DRB1*0401 | DRB1*0801 | DRB1*1101 | DRB1*1301 | DRB1*1501 |
| ITRFQTLLALHRSYL | 6 | DRB1*0101 | DRB1*0401 | DRB1*0801 | DRB1*1101 | DRB1*1301 | DRB1*1501 |
| NITRFQTLLALHRSY | 5 | DRB1*0101 | DRB1*0401 | DRB1*0801 | DRB1*1101 | DRB1*1501 |  |
| RFQTLLALHRSYLTP | 5 | DRB1*0101 | DRB1*0801 | DRB1*1101 | DRB1*1301 | DRB1*1501 |  |
| INITRFQTLLALHRS | 5 | DRB1*0101 | DRB1*0401 | DRB1*0801 | DRB1*1101 | DRB1*1501 |  |
| GINITRFQTLLALHR | 5 | DRB1*0101 | DRB1*0401 | DRB1*0801 | DRB1*1101 | DRB1*1501 |  |
| FQTLLALHRSYLTPG | 4 | DRB1*0801 | DRB1*1101 | DRB1*1301 | DRB1*1501 |  |  |
| IIAYTMSLGAENSVA | 3 | DRB1*0101 | DRB1*0401 | DRB1*0701 |  |  |  |
| SIIAYTMSLGAENSV | 3 | DRB1*0101 | DRB1*0401 | DRB1*0701 |  |  |  |
| QSIIAYTMSLGAENS | 3 | DRB1*0101 | DRB1*0401 | DRB1*0701 |  |  |  |
| NYNYLYRLFRKSNLK | 3 | DRB1*0801 | DRB1*1101 | DRB1*1501 |  |  |  |
| YNYLYRLFRKSNLKP | 3 | DRB1*0801 | DRB1*1101 | DRB1*1501 |  |  |  |
| NYLYRLFRKSNLKPF | 3 | DRB1*0801 | DRB1*1101 | DRB1*1501 |  |  |  |
| GNYNYLYRLFRKSNL | 3 | DRB1*0801 | DRB1*1101 | DRB1*1501 |  |  |  |
