# Supplementary material for: Multiepitope Subunit Vaccine Design against COVID-19 Based on the Spike Protein of SARS-CoV-2: An In Silico Analysis
Source: J Immunol Res. 2020 Nov 19;2020:8893483. doi: 10.1155/2020/8893483 (PMC7678744; doi:10.1155/2020/8893483)
Supplement: Supplementary Materials — Table S1: epitope filtration steps to finalize HLA I epitopes in the SARS-CoV-2 spike glycoprotein sequence. Table S2: epitope filtration steps to finalize HLA II epitopes in the SARS-CoV-2 spike glycoprotein sequence after PREDIVAC and other tools. Table S3: epitope filtration steps to finalize HLA II epitopes in the SARS-CoV-2 spike glycoprotein sequence after NetMHCIIpan and other tools. Table S4: predicted B-cell linear epitopes in the SARS-CoV-2 glycoprotein with probability values. Table S5: predicted discontinuous B-cell epitopes in the multiepitope vaccine according to the DiscoTope server. Figure S1: graphs obtained after molecular docking between vaccine and TLR3 structures. Figure S2: graphs obtained after applying refinements on the top vaccine-TLR3 docked structure. [file 8893483.f1.zip › Supplementary Table 4.docx]

Supplementary Table 4

| **S.NO** | **FASTA Sequence** | **PIP** | **Prob** |
| --- | --- | --- | --- |
| 1 | MFVFLVLLPLVSSQCVNLTT | BCE | 0.5991 |
| 2 | FVFLVLLPLVSSQCVNLTTR | BCE | 0.5226 |
| 3 | LVLLPLVSSQCVNLTTRTQL | BCE | 0.5707 |
| 4 | VLLPLVSSQCVNLTTRTQLP | BCE | 0.7975 |
| 5 | LLPLVSSQCVNLTTRTQLPP | BCE | 0.7717 |
| 6 | LPLVSSQCVNLTTRTQLPPA | BCE | 0.7675 |
| 7 | PLVSSQCVNLTTRTQLPPAY | BCE | 0.7242 |
| 8 | LVSSQCVNLTTRTQLPPAYT | BCE | 0.7261 |
| 9 | VSSQCVNLTTRTQLPPAYTN | BCE | 0.7216 |
| 10 | SSQCVNLTTRTQLPPAYTNS | BCE | 0.712 |
| 11 | SQCVNLTTRTQLPPAYTNSF | BCE | 0.677 |
| 12 | QCVNLTTRTQLPPAYTNSFT | BCE | 0.6033 |
| 13 | CVNLTTRTQLPPAYTNSFTR | BCE | 0.5245 |
| 14 | VNLTTRTQLPPAYTNSFTRG | BCE | 0.5413 |
| 15 | NLTTRTQLPPAYTNSFTRGV | BCE | 0.6377 |
| 16 | LTTRTQLPPAYTNSFTRGVY | BCE | 0.7235 |
| 17 | TTRTQLPPAYTNSFTRGVYY | BCE | 0.6995 |
| 18 | TRTQLPPAYTNSFTRGVYYP | BCE | 0.7389 |
| 19 | RTQLPPAYTNSFTRGVYYPD | BCE | 0.7803 |
| 20 | TQLPPAYTNSFTRGVYYPDK | BCE | 0.7841 |
| 21 | QLPPAYTNSFTRGVYYPDKV | BCE | 0.8136 |
| 22 | LPPAYTNSFTRGVYYPDKVF | BCE | 0.7988 |
| 23 | PPAYTNSFTRGVYYPDKVFR | BCE | 0.7943 |
| 24 | PAYTNSFTRGVYYPDKVFRS | BCE | 0.8012 |
| 25 | AYTNSFTRGVYYPDKVFRSS | BCE | 0.8258 |
| 26 | YTNSFTRGVYYPDKVFRSSV | BCE | 0.8032 |
| 27 | TNSFTRGVYYPDKVFRSSVL | BCE | 0.794 |
| 28 | NSFTRGVYYPDKVFRSSVLH | BCE | 0.7468 |
| 29 | SFTRGVYYPDKVFRSSVLHS | BCE | 0.6851 |
| 30 | FTRGVYYPDKVFRSSVLHST | BCE | 0.7137 |
| 31 | TRGVYYPDKVFRSSVLHSTQ | BCE | 0.7158 |
| 32 | RGVYYPDKVFRSSVLHSTQD | BCE | 0.7235 |
| 33 | GVYYPDKVFRSSVLHSTQDL | BCE | 0.6822 |
| 34 | VYYPDKVFRSSVLHSTQDLF | BCE | 0.7414 |
| 35 | YYPDKVFRSSVLHSTQDLFL | BCE | 0.739 |
| 36 | YPDKVFRSSVLHSTQDLFLP | BCE | 0.6661 |
| 37 | PDKVFRSSVLHSTQDLFLPF | BCE | 0.714 |
| 38 | DKVFRSSVLHSTQDLFLPFF | BCE | 0.6743 |
| 39 | KVFRSSVLHSTQDLFLPFFS | BCE | 0.6111 |
| 40 | VFRSSVLHSTQDLFLPFFSN | BCE | 0.5011 |
| 41 | FRSSVLHSTQDLFLPFFSNV | BCE | 0.5575 |
| 42 | RSSVLHSTQDLFLPFFSNVT | BCE | 0.6194 |
| 43 | SSVLHSTQDLFLPFFSNVTW | BCE | 0.6613 |
| 44 | SVLHSTQDLFLPFFSNVTWF | BCE | 0.5788 |
| 45 | VLHSTQDLFLPFFSNVTWFH | BCE | 0.6495 |
| 46 | LHSTQDLFLPFFSNVTWFHA | BCE | 0.7634 |
| 47 | HSTQDLFLPFFSNVTWFHAI | BCE | 0.7633 |
| 48 | STQDLFLPFFSNVTWFHAIH | BCE | 0.7891 |
| 49 | TQDLFLPFFSNVTWFHAIHV | BCE | 0.7569 |
| 50 | QDLFLPFFSNVTWFHAIHVS | BCE | 0.7588 |
| 51 | DLFLPFFSNVTWFHAIHVSG | BCE | 0.7741 |
| 52 | LFLPFFSNVTWFHAIHVSGT | BCE | 0.7591 |
| 53 | FLPFFSNVTWFHAIHVSGTN | BCE | 0.7211 |
| 54 | LPFFSNVTWFHAIHVSGTNG | BCE | 0.6914 |
| 55 | PFFSNVTWFHAIHVSGTNGT | BCE | 0.6146 |
| 56 | FFSNVTWFHAIHVSGTNGTK | BCE | 0.5085 |
| 57 | NVTWFHAIHVSGTNGTKRFD | BCE | 0.7365 |
| 58 | VTWFHAIHVSGTNGTKRFDN | BCE | 0.7574 |
| 59 | TWFHAIHVSGTNGTKRFDNP | BCE | 0.7611 |
| 60 | WFHAIHVSGTNGTKRFDNPV | BCE | 0.7242 |
| 61 | FHAIHVSGTNGTKRFDNPVL | BCE | 0.7153 |
| 62 | HAIHVSGTNGTKRFDNPVLP | BCE | 0.5901 |
| 63 | AIHVSGTNGTKRFDNPVLPF | BCE | 0.6183 |
| 64 | IHVSGTNGTKRFDNPVLPFN | BCE | 0.5432 |
| 65 | HVSGTNGTKRFDNPVLPFND | BCE | 0.7265 |
| 66 | VSGTNGTKRFDNPVLPFNDG | BCE | 0.7439 |
| 67 | SGTNGTKRFDNPVLPFNDGV | BCE | 0.7022 |
| 68 | GTNGTKRFDNPVLPFNDGVY | BCE | 0.6872 |
| 69 | TNGTKRFDNPVLPFNDGVYF | BCE | 0.7154 |
| 70 | NGTKRFDNPVLPFNDGVYFA | BCE | 0.5876 |
| 71 | GTKRFDNPVLPFNDGVYFAS | BCE | 0.5549 |
| 72 | TKRFDNPVLPFNDGVYFAST | BCE | 0.6139 |
| 73 | KRFDNPVLPFNDGVYFASTE | BCE | 0.725 |
| 74 | RFDNPVLPFNDGVYFASTEK | BCE | 0.7617 |
| 75 | FDNPVLPFNDGVYFASTEKS | BCE | 0.7751 |
| 76 | DNPVLPFNDGVYFASTEKSN | BCE | 0.8091 |
| 77 | NPVLPFNDGVYFASTEKSNI | BCE | 0.8247 |
| 78 | PVLPFNDGVYFASTEKSNII | BCE | 0.791 |
| 79 | VLPFNDGVYFASTEKSNIIR | BCE | 0.784 |
| 80 | LPFNDGVYFASTEKSNIIRG | BCE | 0.725 |
| 81 | PFNDGVYFASTEKSNIIRGW | BCE | 0.7261 |
| 82 | FNDGVYFASTEKSNIIRGWI | BCE | 0.7119 |
| 83 | NDGVYFASTEKSNIIRGWIF | BCE | 0.7106 |
| 84 | DGVYFASTEKSNIIRGWIFG | BCE | 0.6939 |
| 85 | GVYFASTEKSNIIRGWIFGT | BCE | 0.7122 |
| 86 | VYFASTEKSNIIRGWIFGTT | BCE | 0.7197 |
| 87 | YFASTEKSNIIRGWIFGTTL | BCE | 0.6938 |
| 88 | FASTEKSNIIRGWIFGTTLD | BCE | 0.727 |
| 89 | ASTEKSNIIRGWIFGTTLDS | BCE | 0.734 |
| 90 | STEKSNIIRGWIFGTTLDSK | BCE | 0.7489 |
| 91 | TEKSNIIRGWIFGTTLDSKT | BCE | 0.739 |
| 92 | EKSNIIRGWIFGTTLDSKTQ | BCE | 0.716 |
| 93 | KSNIIRGWIFGTTLDSKTQS | BCE | 0.7384 |
| 94 | SNIIRGWIFGTTLDSKTQSL | BCE | 0.7498 |
| 95 | NIIRGWIFGTTLDSKTQSLL | BCE | 0.7113 |
| 96 | IIRGWIFGTTLDSKTQSLLI | BCE | 0.74 |
| 97 | IRGWIFGTTLDSKTQSLLIV | BCE | 0.7276 |
| 98 | RGWIFGTTLDSKTQSLLIVN | BCE | 0.7241 |
| 99 | GWIFGTTLDSKTQSLLIVNN | BCE | 0.6813 |
| 100 | WIFGTTLDSKTQSLLIVNNA | BCE | 0.6012 |
| 101 | IFGTTLDSKTQSLLIVNNAT | BCE | 0.6858 |
| 102 | FGTTLDSKTQSLLIVNNATN | BCE | 0.7154 |
| 103 | GTTLDSKTQSLLIVNNATNV | BCE | 0.6701 |
| 104 | TTLDSKTQSLLIVNNATNVV | BCE | 0.7523 |
| 105 | TLDSKTQSLLIVNNATNVVI | BCE | 0.7773 |
| 106 | LDSKTQSLLIVNNATNVVIK | BCE | 0.7763 |
| 107 | DSKTQSLLIVNNATNVVIKV | BCE | 0.8072 |
| 108 | SKTQSLLIVNNATNVVIKVC | BCE | 0.7853 |
| 109 | KTQSLLIVNNATNVVIKVCE | BCE | 0.8221 |
| 110 | TQSLLIVNNATNVVIKVCEF | BCE | 0.8093 |
| 111 | QSLLIVNNATNVVIKVCEFQ | BCE | 0.8196 |
| 112 | SLLIVNNATNVVIKVCEFQF | BCE | 0.7841 |
| 113 | LLIVNNATNVVIKVCEFQFC | BCE | 0.8005 |
| 114 | LIVNNATNVVIKVCEFQFCN | BCE | 0.794 |
| 115 | IVNNATNVVIKVCEFQFCND | BCE | 0.8012 |
| 116 | VNNATNVVIKVCEFQFCNDP | BCE | 0.8168 |
| 117 | NNATNVVIKVCEFQFCNDPF | BCE | 0.8136 |
| 118 | NATNVVIKVCEFQFCNDPFL | BCE | 0.7872 |
| 119 | ATNVVIKVCEFQFCNDPFLG | BCE | 0.7834 |
| 120 | TNVVIKVCEFQFCNDPFLGV | BCE | 0.7802 |
| 121 | NVVIKVCEFQFCNDPFLGVY | BCE | 0.7688 |
| 122 | VVIKVCEFQFCNDPFLGVYY | BCE | 0.7428 |
| 123 | VIKVCEFQFCNDPFLGVYYH | BCE | 0.7123 |
| 124 | IKVCEFQFCNDPFLGVYYHK | BCE | 0.7528 |
| 125 | KVCEFQFCNDPFLGVYYHKN | BCE | 0.7516 |
| 126 | VCEFQFCNDPFLGVYYHKNN | BCE | 0.7878 |
| 127 | CEFQFCNDPFLGVYYHKNNK | BCE | 0.8404 |
| 128 | EFQFCNDPFLGVYYHKNNKS | BCE | 0.8126 |
| 129 | FQFCNDPFLGVYYHKNNKSW | BCE | 0.7807 |
| 130 | QFCNDPFLGVYYHKNNKSWM | BCE | 0.7608 |
| 131 | FCNDPFLGVYYHKNNKSWME | BCE | 0.7979 |
| 132 | CNDPFLGVYYHKNNKSWMES | BCE | 0.8058 |
| 133 | NDPFLGVYYHKNNKSWMESE | BCE | 0.8234 |
| 134 | DPFLGVYYHKNNKSWMESEF | BCE | 0.7909 |
| 135 | PFLGVYYHKNNKSWMESEFR | BCE | 0.7532 |
| 136 | FLGVYYHKNNKSWMESEFRV | BCE | 0.7424 |
| 137 | LGVYYHKNNKSWMESEFRVY | BCE | 0.7336 |
| 138 | GVYYHKNNKSWMESEFRVYS | BCE | 0.7749 |
| 139 | VYYHKNNKSWMESEFRVYSS | BCE | 0.7909 |
| 140 | YYHKNNKSWMESEFRVYSSA | BCE | 0.8054 |
| 141 | YHKNNKSWMESEFRVYSSAN | BCE | 0.8349 |
| 142 | HKNNKSWMESEFRVYSSANN | BCE | 0.8319 |
| 143 | KNNKSWMESEFRVYSSANNC | BCE | 0.8214 |
| 144 | NNKSWMESEFRVYSSANNCT | BCE | 0.8295 |
| 145 | NKSWMESEFRVYSSANNCTF | BCE | 0.8297 |
| 146 | KSWMESEFRVYSSANNCTFE | BCE | 0.8245 |
| 147 | SWMESEFRVYSSANNCTFEY | BCE | 0.8135 |
| 148 | WMESEFRVYSSANNCTFEYV | BCE | 0.7776 |
| 149 | MESEFRVYSSANNCTFEYVS | BCE | 0.8015 |
| 150 | ESEFRVYSSANNCTFEYVSQ | BCE | 0.7916 |
| 151 | SEFRVYSSANNCTFEYVSQP | BCE | 0.8083 |
| 152 | EFRVYSSANNCTFEYVSQPF | BCE | 0.7791 |
| 153 | FRVYSSANNCTFEYVSQPFL | BCE | 0.7062 |
| 154 | RVYSSANNCTFEYVSQPFLM | BCE | 0.7027 |
| 155 | VYSSANNCTFEYVSQPFLMD | BCE | 0.771 |
| 156 | YSSANNCTFEYVSQPFLMDL | BCE | 0.7783 |
| 157 | SSANNCTFEYVSQPFLMDLE | BCE | 0.7508 |
| 158 | SANNCTFEYVSQPFLMDLEG | BCE | 0.7504 |
| 159 | ANNCTFEYVSQPFLMDLEGK | BCE | 0.7563 |
| 160 | NNCTFEYVSQPFLMDLEGKQ | BCE | 0.7469 |
| 161 | NCTFEYVSQPFLMDLEGKQG | BCE | 0.7006 |
| 162 | CTFEYVSQPFLMDLEGKQGN | BCE | 0.7018 |
| 163 | TFEYVSQPFLMDLEGKQGNF | BCE | 0.7188 |
| 164 | FEYVSQPFLMDLEGKQGNFK | BCE | 0.7631 |
| 165 | EYVSQPFLMDLEGKQGNFKN | BCE | 0.7627 |
| 166 | YVSQPFLMDLEGKQGNFKNL | BCE | 0.7507 |
| 167 | VSQPFLMDLEGKQGNFKNLR | BCE | 0.7041 |
| 168 | SQPFLMDLEGKQGNFKNLRE | BCE | 0.7633 |
| 169 | QPFLMDLEGKQGNFKNLREF | BCE | 0.7942 |
| 170 | PFLMDLEGKQGNFKNLREFV | BCE | 0.7936 |
| 171 | FLMDLEGKQGNFKNLREFVF | BCE | 0.6982 |
| 172 | LMDLEGKQGNFKNLREFVFK | BCE | 0.7691 |
| 173 | MDLEGKQGNFKNLREFVFKN | BCE | 0.7487 |
| 174 | DLEGKQGNFKNLREFVFKNI | BCE | 0.7353 |
| 175 | LEGKQGNFKNLREFVFKNID | BCE | 0.6981 |
| 176 | GKQGNFKNLREFVFKNIDGY | BCE | 0.6711 |
| 177 | KQGNFKNLREFVFKNIDGYF | BCE | 0.6973 |
| 178 | QGNFKNLREFVFKNIDGYFK | BCE | 0.664 |
| 179 | GNFKNLREFVFKNIDGYFKI | BCE | 0.561 |
| 180 | NFKNLREFVFKNIDGYFKIY | BCE | 0.6799 |
| 181 | KNLREFVFKNIDGYFKIYSK | BCE | 0.7142 |
| 182 | NLREFVFKNIDGYFKIYSKH | BCE | 0.6704 |
| 183 | LREFVFKNIDGYFKIYSKHT | BCE | 0.6206 |
| 184 | REFVFKNIDGYFKIYSKHTP | BCE | 0.6342 |
| 185 | EFVFKNIDGYFKIYSKHTPI | BCE | 0.5172 |
| 186 | FVFKNIDGYFKIYSKHTPIN | BCE | 0.5538 |
| 187 | VFKNIDGYFKIYSKHTPINL | BCE | 0.5753 |
| 188 | FKNIDGYFKIYSKHTPINLV | BCE | 0.7281 |
| 189 | KNIDGYFKIYSKHTPINLVR | BCE | 0.8192 |
| 190 | NIDGYFKIYSKHTPINLVRD | BCE | 0.7802 |
| 191 | IDGYFKIYSKHTPINLVRDL | BCE | 0.7488 |
| 192 | DGYFKIYSKHTPINLVRDLP | BCE | 0.7407 |
| 193 | GYFKIYSKHTPINLVRDLPQ | BCE | 0.7565 |
| 194 | YFKIYSKHTPINLVRDLPQG | BCE | 0.7626 |
| 195 | FKIYSKHTPINLVRDLPQGF | BCE | 0.7211 |
| 196 | KIYSKHTPINLVRDLPQGFS | BCE | 0.641 |
| 197 | IYSKHTPINLVRDLPQGFSA | BCE | 0.6489 |
| 198 | YSKHTPINLVRDLPQGFSAL | BCE | 0.6567 |
| 199 | SKHTPINLVRDLPQGFSALE | BCE | 0.6998 |
| 200 | KHTPINLVRDLPQGFSALEP | BCE | 0.6955 |
| 201 | HTPINLVRDLPQGFSALEPL | BCE | 0.6874 |
| 202 | TPINLVRDLPQGFSALEPLV | BCE | 0.6283 |
| 203 | PINLVRDLPQGFSALEPLVD | BCE | 0.782 |
| 204 | INLVRDLPQGFSALEPLVDL | BCE | 0.6094 |
| 205 | NLVRDLPQGFSALEPLVDLP | BCE | 0.7751 |
| 206 | LVRDLPQGFSALEPLVDLPI | BCE | 0.7266 |
| 207 | VRDLPQGFSALEPLVDLPIG | BCE | 0.7853 |
| 208 | RDLPQGFSALEPLVDLPIGI | BCE | 0.7788 |
| 209 | DLPQGFSALEPLVDLPIGIN | BCE | 0.7997 |
| 210 | LPQGFSALEPLVDLPIGINI | BCE | 0.7069 |
| 211 | PQGFSALEPLVDLPIGINIT | BCE | 0.5856 |
| 212 | QGFSALEPLVDLPIGINITR | BCE | 0.5543 |
| 213 | FSALEPLVDLPIGINITRFQ | BCE | 0.5189 |
| 214 | SALEPLVDLPIGINITRFQT | BCE | 0.6827 |
| 215 | ALEPLVDLPIGINITRFQTL | BCE | 0.5831 |
| 216 | LEPLVDLPIGINITRFQTLL | BCE | 0.611 |
| 217 | EPLVDLPIGINITRFQTLLA | BCE | 0.5469 |
| 218 | PLVDLPIGINITRFQTLLAL | BCE | 0.6409 |
| 219 | LVDLPIGINITRFQTLLALH | BCE | 0.6709 |
| 220 | VDLPIGINITRFQTLLALHR | BCE | 0.7094 |
| 221 | DLPIGINITRFQTLLALHRS | BCE | 0.7724 |
| 222 | LPIGINITRFQTLLALHRSY | BCE | 0.7738 |
| 223 | PIGINITRFQTLLALHRSYL | BCE | 0.7749 |
| 224 | IGINITRFQTLLALHRSYLT | BCE | 0.7507 |
| 225 | GINITRFQTLLALHRSYLTP | BCE | 0.7636 |
| 226 | INITRFQTLLALHRSYLTPG | BCE | 0.7678 |
| 227 | NITRFQTLLALHRSYLTPGD | BCE | 0.7536 |
| 228 | ITRFQTLLALHRSYLTPGDS | BCE | 0.7277 |
| 229 | TRFQTLLALHRSYLTPGDSS | BCE | 0.7561 |
| 230 | RFQTLLALHRSYLTPGDSSS | BCE | 0.7821 |
| 231 | FQTLLALHRSYLTPGDSSSG | BCE | 0.7582 |
| 232 | QTLLALHRSYLTPGDSSSGW | BCE | 0.7711 |
| 233 | TLLALHRSYLTPGDSSSGWT | BCE | 0.7517 |
| 234 | LLALHRSYLTPGDSSSGWTA | BCE | 0.7367 |
| 235 | LALHRSYLTPGDSSSGWTAG | BCE | 0.7459 |
| 236 | ALHRSYLTPGDSSSGWTAGA | BCE | 0.7606 |
| 237 | LHRSYLTPGDSSSGWTAGAA | BCE | 0.7643 |
| 238 | HRSYLTPGDSSSGWTAGAAA | BCE | 0.8056 |
| 239 | RSYLTPGDSSSGWTAGAAAY | BCE | 0.7728 |
| 240 | SYLTPGDSSSGWTAGAAAYY | BCE | 0.7753 |
| 241 | YLTPGDSSSGWTAGAAAYYV | BCE | 0.7028 |
| 242 | LTPGDSSSGWTAGAAAYYVG | BCE | 0.7467 |
| 243 | TPGDSSSGWTAGAAAYYVGY | BCE | 0.7835 |
| 244 | PGDSSSGWTAGAAAYYVGYL | BCE | 0.761 |
| 245 | GDSSSGWTAGAAAYYVGYLQ | BCE | 0.7534 |
| 246 | DSSSGWTAGAAAYYVGYLQP | BCE | 0.7625 |
| 247 | SSSGWTAGAAAYYVGYLQPR | BCE | 0.7122 |
| 248 | SSGWTAGAAAYYVGYLQPRT | BCE | 0.7369 |
| 249 | SGWTAGAAAYYVGYLQPRTF | BCE | 0.7637 |
| 250 | GWTAGAAAYYVGYLQPRTFL | BCE | 0.7487 |
| 251 | WTAGAAAYYVGYLQPRTFLL | BCE | 0.7073 |
| 252 | TAGAAAYYVGYLQPRTFLLK | BCE | 0.7846 |
| 253 | AGAAAYYVGYLQPRTFLLKY | BCE | 0.7734 |
| 254 | GAAAYYVGYLQPRTFLLKYN | BCE | 0.7689 |
| 255 | AAAYYVGYLQPRTFLLKYNE | BCE | 0.801 |
| 256 | AAYYVGYLQPRTFLLKYNEN | BCE | 0.8269 |
| 257 | AYYVGYLQPRTFLLKYNENG | BCE | 0.7869 |
| 258 | YYVGYLQPRTFLLKYNENGT | BCE | 0.8179 |
| 259 | YVGYLQPRTFLLKYNENGTI | BCE | 0.8247 |
| 260 | VGYLQPRTFLLKYNENGTIT | BCE | 0.7513 |
| 261 | GYLQPRTFLLKYNENGTITD | BCE | 0.7304 |
| 262 | YLQPRTFLLKYNENGTITDA | BCE | 0.7462 |
| 263 | LQPRTFLLKYNENGTITDAV | BCE | 0.6531 |
| 264 | QPRTFLLKYNENGTITDAVD | BCE | 0.7407 |
| 265 | PRTFLLKYNENGTITDAVDC | BCE | 0.6836 |
| 266 | RTFLLKYNENGTITDAVDCA | BCE | 0.737 |
| 267 | TFLLKYNENGTITDAVDCAL | BCE | 0.7665 |
| 268 | FLLKYNENGTITDAVDCALD | BCE | 0.7156 |
| 269 | LLKYNENGTITDAVDCALDP | BCE | 0.7807 |
| 270 | LKYNENGTITDAVDCALDPL | BCE | 0.7876 |
| 271 | KYNENGTITDAVDCALDPLS | BCE | 0.7622 |
| 272 | YNENGTITDAVDCALDPLSE | BCE | 0.7712 |
| 273 | NENGTITDAVDCALDPLSET | BCE | 0.7693 |
| 274 | ENGTITDAVDCALDPLSETK | BCE | 0.7886 |
| 275 | NGTITDAVDCALDPLSETKC | BCE | 0.736 |
| 276 | GTITDAVDCALDPLSETKCT | BCE | 0.6411 |
| 277 | TITDAVDCALDPLSETKCTL | BCE | 0.6303 |
| 278 | ITDAVDCALDPLSETKCTLK | BCE | 0.7054 |
| 279 | TDAVDCALDPLSETKCTLKS | BCE | 0.6599 |
| 280 | DAVDCALDPLSETKCTLKSF | BCE | 0.7047 |
| 281 | AVDCALDPLSETKCTLKSFT | BCE | 0.76 |
| 282 | VDCALDPLSETKCTLKSFTV | BCE | 0.7089 |
| 283 | DCALDPLSETKCTLKSFTVE | BCE | 0.6266 |
| 284 | CALDPLSETKCTLKSFTVEK | BCE | 0.6788 |
| 285 | ALDPLSETKCTLKSFTVEKG | BCE | 0.6887 |
| 286 | LDPLSETKCTLKSFTVEKGI | BCE | 0.6274 |
| 287 | DPLSETKCTLKSFTVEKGIY | BCE | 0.7145 |
| 288 | PLSETKCTLKSFTVEKGIYQ | BCE | 0.7509 |
| 289 | LSETKCTLKSFTVEKGIYQT | BCE | 0.7827 |
| 290 | SETKCTLKSFTVEKGIYQTS | BCE | 0.7626 |
| 291 | ETKCTLKSFTVEKGIYQTSN | BCE | 0.6976 |
| 292 | TKCTLKSFTVEKGIYQTSNF | BCE | 0.7899 |
| 293 | KCTLKSFTVEKGIYQTSNFR | BCE | 0.7667 |
| 294 | CTLKSFTVEKGIYQTSNFRV | BCE | 0.7726 |
| 295 | TLKSFTVEKGIYQTSNFRVQ | BCE | 0.7937 |
| 296 | LKSFTVEKGIYQTSNFRVQP | BCE | 0.7866 |
| 297 | KSFTVEKGIYQTSNFRVQPT | BCE | 0.7804 |
| 298 | SFTVEKGIYQTSNFRVQPTE | BCE | 0.7878 |
| 299 | FTVEKGIYQTSNFRVQPTES | BCE | 0.7879 |
| 300 | TVEKGIYQTSNFRVQPTESI | BCE | 0.7667 |
| 301 | VEKGIYQTSNFRVQPTESIV | BCE | 0.7822 |
| 302 | EKGIYQTSNFRVQPTESIVR | BCE | 0.7651 |
| 303 | KGIYQTSNFRVQPTESIVRF | BCE | 0.7971 |
| 304 | GIYQTSNFRVQPTESIVRFP | BCE | 0.8034 |
| 305 | IYQTSNFRVQPTESIVRFPN | BCE | 0.8131 |
| 306 | YQTSNFRVQPTESIVRFPNI | BCE | 0.812 |
| 307 | QTSNFRVQPTESIVRFPNIT | BCE | 0.7809 |
| 308 | TSNFRVQPTESIVRFPNITN | BCE | 0.7808 |
| 309 | SNFRVQPTESIVRFPNITNL | BCE | 0.7864 |
| 310 | NFRVQPTESIVRFPNITNLC | BCE | 0.793 |
| 311 | FRVQPTESIVRFPNITNLCP | BCE | 0.7984 |
| 312 | RVQPTESIVRFPNITNLCPF | BCE | 0.8009 |
| 313 | VQPTESIVRFPNITNLCPFG | BCE | 0.6364 |
| 314 | QPTESIVRFPNITNLCPFGE | BCE | 0.7502 |
| 315 | PTESIVRFPNITNLCPFGEV | BCE | 0.7562 |
| 316 | TESIVRFPNITNLCPFGEVF | BCE | 0.6514 |
| 317 | ESIVRFPNITNLCPFGEVFN | BCE | 0.6332 |
| 318 | SIVRFPNITNLCPFGEVFNA | BCE | 0.5807 |
| 319 | VRFPNITNLCPFGEVFNATR | BCE | 0.6304 |
| 320 | FPNITNLCPFGEVFNATRFA | BCE | 0.5957 |
| 321 | NITNLCPFGEVFNATRFASV | BCE | 0.633 |
| 322 | ITNLCPFGEVFNATRFASVY | BCE | 0.6399 |
| 323 | TNLCPFGEVFNATRFASVYA | BCE | 0.66 |
| 324 | NLCPFGEVFNATRFASVYAW | BCE | 0.6505 |
| 325 | LCPFGEVFNATRFASVYAWN | BCE | 0.6105 |
| 326 | CPFGEVFNATRFASVYAWNR | BCE | 0.6384 |
| 327 | PFGEVFNATRFASVYAWNRK | BCE | 0.6762 |
| 328 | FGEVFNATRFASVYAWNRKR | BCE | 0.6095 |
| 329 | GEVFNATRFASVYAWNRKRI | BCE | 0.5736 |
| 330 | EVFNATRFASVYAWNRKRIS | BCE | 0.5727 |
| 331 | VFNATRFASVYAWNRKRISN | BCE | 0.5961 |
| 332 | FNATRFASVYAWNRKRISNC | BCE | 0.6484 |
| 333 | NATRFASVYAWNRKRISNCV | BCE | 0.6581 |
| 334 | ATRFASVYAWNRKRISNCVA | BCE | 0.6085 |
| 335 | TRFASVYAWNRKRISNCVAD | BCE | 0.7275 |
| 336 | RFASVYAWNRKRISNCVADY | BCE | 0.6072 |
| 337 | FASVYAWNRKRISNCVADYS | BCE | 0.7495 |
| 338 | ASVYAWNRKRISNCVADYSV | BCE | 0.6996 |
| 339 | SVYAWNRKRISNCVADYSVL | BCE | 0.6938 |
| 340 | VYAWNRKRISNCVADYSVLY | BCE | 0.7174 |
| 341 | YAWNRKRISNCVADYSVLYN | BCE | 0.7221 |
| 342 | AWNRKRISNCVADYSVLYNS | BCE | 0.7539 |
| 343 | WNRKRISNCVADYSVLYNSA | BCE | 0.7645 |
| 344 | NRKRISNCVADYSVLYNSAS | BCE | 0.8021 |
| 345 | RKRISNCVADYSVLYNSASF | BCE | 0.7997 |
| 346 | KRISNCVADYSVLYNSASFS | BCE | 0.6771 |
| 347 | RISNCVADYSVLYNSASFST | BCE | 0.6387 |
| 348 | ISNCVADYSVLYNSASFSTF | BCE | 0.7239 |
| 349 | SNCVADYSVLYNSASFSTFK | BCE | 0.7357 |
| 350 | NCVADYSVLYNSASFSTFKC | BCE | 0.7978 |
| 351 | CVADYSVLYNSASFSTFKCY | BCE | 0.7421 |
| 352 | VADYSVLYNSASFSTFKCYG | BCE | 0.7527 |
| 353 | ADYSVLYNSASFSTFKCYGV | BCE | 0.7474 |
| 354 | DYSVLYNSASFSTFKCYGVS | BCE | 0.7594 |
| 355 | YSVLYNSASFSTFKCYGVSP | BCE | 0.7615 |
| 356 | SVLYNSASFSTFKCYGVSPT | BCE | 0.7795 |
| 357 | VLYNSASFSTFKCYGVSPTK | BCE | 0.7492 |
| 358 | LYNSASFSTFKCYGVSPTKL | BCE | 0.7132 |
| 359 | YNSASFSTFKCYGVSPTKLN | BCE | 0.8022 |
| 360 | NSASFSTFKCYGVSPTKLND | BCE | 0.7885 |
| 361 | SASFSTFKCYGVSPTKLNDL | BCE | 0.7783 |
| 362 | ASFSTFKCYGVSPTKLNDLC | BCE | 0.8543 |
| 363 | SFSTFKCYGVSPTKLNDLCF | BCE | 0.8157 |
| 364 | FSTFKCYGVSPTKLNDLCFT | BCE | 0.7984 |
| 365 | STFKCYGVSPTKLNDLCFTN | BCE | 0.8443 |
| 366 | TFKCYGVSPTKLNDLCFTNV | BCE | 0.8594 |
| 367 | FKCYGVSPTKLNDLCFTNVY | BCE | 0.8535 |
| 368 | KCYGVSPTKLNDLCFTNVYA | BCE | 0.8792 |
| 369 | CYGVSPTKLNDLCFTNVYAD | BCE | 0.8688 |
| 370 | YGVSPTKLNDLCFTNVYADS | BCE | 0.8312 |
| 371 | GVSPTKLNDLCFTNVYADSF | BCE | 0.8281 |
| 372 | VSPTKLNDLCFTNVYADSFV | BCE | 0.83 |
| 373 | SPTKLNDLCFTNVYADSFVI | BCE | 0.8387 |
| 374 | PTKLNDLCFTNVYADSFVIR | BCE | 0.804 |
| 375 | TKLNDLCFTNVYADSFVIRG | BCE | 0.7887 |
| 376 | KLNDLCFTNVYADSFVIRGD | BCE | 0.7891 |
| 377 | LNDLCFTNVYADSFVIRGDE | BCE | 0.7485 |
| 378 | NDLCFTNVYADSFVIRGDEV | BCE | 0.7248 |
| 379 | DLCFTNVYADSFVIRGDEVR | BCE | 0.6979 |
| 380 | LCFTNVYADSFVIRGDEVRQ | BCE | 0.6527 |
| 381 | CFTNVYADSFVIRGDEVRQI | BCE | 0.623 |
| 382 | FTNVYADSFVIRGDEVRQIA | BCE | 0.6656 |
| 383 | TNVYADSFVIRGDEVRQIAP | BCE | 0.7205 |
| 384 | NVYADSFVIRGDEVRQIAPG | BCE | 0.7077 |
| 385 | VYADSFVIRGDEVRQIAPGQ | BCE | 0.6657 |
| 386 | YADSFVIRGDEVRQIAPGQT | BCE | 0.6319 |
| 387 | ADSFVIRGDEVRQIAPGQTG | BCE | 0.6796 |
| 388 | DSFVIRGDEVRQIAPGQTGK | BCE | 0.5452 |
| 389 | FVIRGDEVRQIAPGQTGKIA | BCE | 0.5432 |
| 390 | VIRGDEVRQIAPGQTGKIAD | BCE | 0.5283 |
| 391 | RGDEVRQIAPGQTGKIADYN | BCE | 0.6664 |
| 392 | GDEVRQIAPGQTGKIADYNY | BCE | 0.5978 |
| 393 | DEVRQIAPGQTGKIADYNYK | BCE | 0.7273 |
| 394 | EVRQIAPGQTGKIADYNYKL | BCE | 0.7979 |
| 395 | VRQIAPGQTGKIADYNYKLP | BCE | 0.731 |
| 396 | RQIAPGQTGKIADYNYKLPD | BCE | 0.7817 |
| 397 | QIAPGQTGKIADYNYKLPDD | BCE | 0.7851 |
| 398 | IAPGQTGKIADYNYKLPDDF | BCE | 0.8056 |
| 399 | APGQTGKIADYNYKLPDDFT | BCE | 0.8458 |
| 400 | PGQTGKIADYNYKLPDDFTG | BCE | 0.8431 |
| 401 | GQTGKIADYNYKLPDDFTGC | BCE | 0.7936 |
| 402 | QTGKIADYNYKLPDDFTGCV | BCE | 0.8139 |
| 403 | TGKIADYNYKLPDDFTGCVI | BCE | 0.7915 |
| 404 | GKIADYNYKLPDDFTGCVIA | BCE | 0.8065 |
| 405 | KIADYNYKLPDDFTGCVIAW | BCE | 0.7867 |
| 406 | IADYNYKLPDDFTGCVIAWN | BCE | 0.7921 |
| 407 | ADYNYKLPDDFTGCVIAWNS | BCE | 0.7938 |
| 408 | DYNYKLPDDFTGCVIAWNSN | BCE | 0.8111 |
| 409 | YNYKLPDDFTGCVIAWNSNN | BCE | 0.762 |
| 410 | NYKLPDDFTGCVIAWNSNNL | BCE | 0.7645 |
| 411 | YKLPDDFTGCVIAWNSNNLD | BCE | 0.8031 |
| 412 | KLPDDFTGCVIAWNSNNLDS | BCE | 0.7884 |
| 413 | LPDDFTGCVIAWNSNNLDSK | BCE | 0.7868 |
| 414 | PDDFTGCVIAWNSNNLDSKV | BCE | 0.8079 |
| 415 | DDFTGCVIAWNSNNLDSKVG | BCE | 0.7716 |
| 416 | DFTGCVIAWNSNNLDSKVGG | BCE | 0.7417 |
| 417 | FTGCVIAWNSNNLDSKVGGN | BCE | 0.6054 |
| 418 | TGCVIAWNSNNLDSKVGGNY | BCE | 0.5882 |
| 419 | GCVIAWNSNNLDSKVGGNYN | BCE | 0.7215 |
| 420 | CVIAWNSNNLDSKVGGNYNY | BCE | 0.705 |
| 421 | VIAWNSNNLDSKVGGNYNYL | BCE | 0.7178 |
| 422 | IAWNSNNLDSKVGGNYNYLY | BCE | 0.7161 |
| 423 | AWNSNNLDSKVGGNYNYLYR | BCE | 0.7748 |
| 424 | WNSNNLDSKVGGNYNYLYRL | BCE | 0.7819 |
| 425 | NSNNLDSKVGGNYNYLYRLF | BCE | 0.7967 |
| 426 | SNNLDSKVGGNYNYLYRLFR | BCE | 0.7793 |
| 427 | NNLDSKVGGNYNYLYRLFRK | BCE | 0.8227 |
| 428 | NLDSKVGGNYNYLYRLFRKS | BCE | 0.8046 |
| 429 | LDSKVGGNYNYLYRLFRKSN | BCE | 0.8042 |
| 430 | DSKVGGNYNYLYRLFRKSNL | BCE | 0.7965 |
| 431 | SKVGGNYNYLYRLFRKSNLK | BCE | 0.8383 |
| 432 | KVGGNYNYLYRLFRKSNLKP | BCE | 0.8415 |
| 433 | VGGNYNYLYRLFRKSNLKPF | BCE | 0.8103 |
| 434 | GGNYNYLYRLFRKSNLKPFE | BCE | 0.8396 |
| 435 | GNYNYLYRLFRKSNLKPFER | BCE | 0.7971 |
| 436 | NYNYLYRLFRKSNLKPFERD | BCE | 0.8447 |
| 437 | YNYLYRLFRKSNLKPFERDI | BCE | 0.8087 |
| 438 | NYLYRLFRKSNLKPFERDIS | BCE | 0.8037 |
| 439 | YLYRLFRKSNLKPFERDIST | BCE | 0.7216 |
| 440 | LYRLFRKSNLKPFERDISTE | BCE | 0.7993 |
| 441 | YRLFRKSNLKPFERDISTEI | BCE | 0.7882 |
| 442 | RLFRKSNLKPFERDISTEIY | BCE | 0.7891 |
| 443 | LFRKSNLKPFERDISTEIYQ | BCE | 0.7869 |
| 444 | FRKSNLKPFERDISTEIYQA | BCE | 0.7937 |
| 445 | RKSNLKPFERDISTEIYQAG | BCE | 0.7857 |
| 446 | KSNLKPFERDISTEIYQAGS | BCE | 0.7418 |
| 447 | SNLKPFERDISTEIYQAGST | BCE | 0.7567 |
| 448 | NLKPFERDISTEIYQAGSTP | BCE | 0.7734 |
| 449 | LKPFERDISTEIYQAGSTPC | BCE | 0.7399 |
| 450 | KPFERDISTEIYQAGSTPCN | BCE | 0.7783 |
| 451 | PFERDISTEIYQAGSTPCNG | BCE | 0.7538 |
| 452 | FERDISTEIYQAGSTPCNGV | BCE | 0.7274 |
| 453 | ERDISTEIYQAGSTPCNGVE | BCE | 0.8176 |
| 454 | RDISTEIYQAGSTPCNGVEG | BCE | 0.7695 |
| 455 | DISTEIYQAGSTPCNGVEGF | BCE | 0.7353 |
| 456 | ISTEIYQAGSTPCNGVEGFN | BCE | 0.7307 |
| 457 | STEIYQAGSTPCNGVEGFNC | BCE | 0.7937 |
| 458 | TEIYQAGSTPCNGVEGFNCY | BCE | 0.792 |
| 459 | EIYQAGSTPCNGVEGFNCYF | BCE | 0.7778 |
| 460 | IYQAGSTPCNGVEGFNCYFP | BCE | 0.8004 |
| 461 | YQAGSTPCNGVEGFNCYFPL | BCE | 0.7929 |
| 462 | QAGSTPCNGVEGFNCYFPLQ | BCE | 0.7394 |
| 463 | AGSTPCNGVEGFNCYFPLQS | BCE | 0.758 |
| 464 | GSTPCNGVEGFNCYFPLQSY | BCE | 0.7978 |
| 465 | STPCNGVEGFNCYFPLQSYG | BCE | 0.796 |
| 466 | TPCNGVEGFNCYFPLQSYGF | BCE | 0.7761 |
| 467 | PCNGVEGFNCYFPLQSYGFQ | BCE | 0.7824 |
| 468 | CNGVEGFNCYFPLQSYGFQP | BCE | 0.7861 |
| 469 | NGVEGFNCYFPLQSYGFQPT | BCE | 0.734 |
| 470 | GVEGFNCYFPLQSYGFQPTN | BCE | 0.7381 |
| 471 | VEGFNCYFPLQSYGFQPTNG | BCE | 0.7337 |
| 472 | EGFNCYFPLQSYGFQPTNGV | BCE | 0.7297 |
| 473 | GFNCYFPLQSYGFQPTNGVG | BCE | 0.7408 |
| 474 | FNCYFPLQSYGFQPTNGVGY | BCE | 0.6873 |
| 475 | NCYFPLQSYGFQPTNGVGYQ | BCE | 0.7293 |
| 476 | CYFPLQSYGFQPTNGVGYQP | BCE | 0.7558 |
| 477 | YFPLQSYGFQPTNGVGYQPY | BCE | 0.7851 |
| 478 | FPLQSYGFQPTNGVGYQPYR | BCE | 0.7732 |
| 479 | PLQSYGFQPTNGVGYQPYRV | BCE | 0.7693 |
| 480 | LQSYGFQPTNGVGYQPYRVV | BCE | 0.7611 |
| 481 | QSYGFQPTNGVGYQPYRVVV | BCE | 0.7582 |
| 482 | SYGFQPTNGVGYQPYRVVVL | BCE | 0.7538 |
| 483 | YGFQPTNGVGYQPYRVVVLS | BCE | 0.7539 |
| 484 | GFQPTNGVGYQPYRVVVLSF | BCE | 0.7093 |
| 485 | FQPTNGVGYQPYRVVVLSFE | BCE | 0.7477 |
| 486 | QPTNGVGYQPYRVVVLSFEL | BCE | 0.7699 |
| 487 | PTNGVGYQPYRVVVLSFELL | BCE | 0.7285 |
| 488 | TNGVGYQPYRVVVLSFELLH | BCE | 0.5994 |
| 489 | NGVGYQPYRVVVLSFELLHA | BCE | 0.75 |
| 490 | GVGYQPYRVVVLSFELLHAP | BCE | 0.7279 |
| 491 | VGYQPYRVVVLSFELLHAPA | BCE | 0.7796 |
| 492 | GYQPYRVVVLSFELLHAPAT | BCE | 0.7054 |
| 493 | YQPYRVVVLSFELLHAPATV | BCE | 0.7375 |
| 494 | QPYRVVVLSFELLHAPATVC | BCE | 0.7167 |
| 495 | PYRVVVLSFELLHAPATVCG | BCE | 0.7328 |
| 496 | YRVVVLSFELLHAPATVCGP | BCE | 0.7266 |
| 497 | RVVVLSFELLHAPATVCGPK | BCE | 0.7608 |
| 498 | VVVLSFELLHAPATVCGPKK | BCE | 0.7473 |
| 499 | VVLSFELLHAPATVCGPKKS | BCE | 0.7175 |
| 500 | VLSFELLHAPATVCGPKKST | BCE | 0.7435 |
| 501 | LSFELLHAPATVCGPKKSTN | BCE | 0.754 |
| 502 | SFELLHAPATVCGPKKSTNL | BCE | 0.7494 |
| 503 | FELLHAPATVCGPKKSTNLV | BCE | 0.7595 |
| 504 | ELLHAPATVCGPKKSTNLVK | BCE | 0.7473 |
| 505 | LLHAPATVCGPKKSTNLVKN | BCE | 0.6624 |
| 506 | LHAPATVCGPKKSTNLVKNK | BCE | 0.7423 |
| 507 | HAPATVCGPKKSTNLVKNKC | BCE | 0.7365 |
| 508 | APATVCGPKKSTNLVKNKCV | BCE | 0.8046 |
| 509 | PATVCGPKKSTNLVKNKCVN | BCE | 0.7961 |
| 510 | ATVCGPKKSTNLVKNKCVNF | BCE | 0.7949 |
| 511 | TVCGPKKSTNLVKNKCVNFN | BCE | 0.7831 |
| 512 | VCGPKKSTNLVKNKCVNFNF | BCE | 0.7719 |
| 513 | CGPKKSTNLVKNKCVNFNFN | BCE | 0.7603 |
| 514 | GPKKSTNLVKNKCVNFNFNG | BCE | 0.7419 |
| 515 | PKKSTNLVKNKCVNFNFNGL | BCE | 0.737 |
| 516 | KKSTNLVKNKCVNFNFNGLT | BCE | 0.7597 |
| 517 | KSTNLVKNKCVNFNFNGLTG | BCE | 0.6915 |
| 518 | STNLVKNKCVNFNFNGLTGT | BCE | 0.6636 |
| 519 | TNLVKNKCVNFNFNGLTGTG | BCE | 0.6192 |
| 520 | NLVKNKCVNFNFNGLTGTGV | BCE | 0.564 |
| 521 | LVKNKCVNFNFNGLTGTGVL | BCE | 0.5145 |
| 522 | VKNKCVNFNFNGLTGTGVLT | BCE | 0.5276 |
| 523 | KNKCVNFNFNGLTGTGVLTE | BCE | 0.5245 |
| 524 | NKCVNFNFNGLTGTGVLTES | BCE | 0.6496 |
| 525 | KCVNFNFNGLTGTGVLTESN | BCE | 0.6438 |
| 526 | CVNFNFNGLTGTGVLTESNK | BCE | 0.6495 |
| 527 | VNFNFNGLTGTGVLTESNKK | BCE | 0.6832 |
| 528 | NFNFNGLTGTGVLTESNKKF | BCE | 0.6681 |
| 529 | FNFNGLTGTGVLTESNKKFL | BCE | 0.6536 |
| 530 | NFNGLTGTGVLTESNKKFLP | BCE | 0.6735 |
| 531 | FNGLTGTGVLTESNKKFLPF | BCE | 0.5485 |
| 532 | NGLTGTGVLTESNKKFLPFQ | BCE | 0.6821 |
| 533 | GLTGTGVLTESNKKFLPFQQ | BCE | 0.6577 |
| 534 | LTGTGVLTESNKKFLPFQQF | BCE | 0.7102 |
| 535 | TGTGVLTESNKKFLPFQQFG | BCE | 0.6885 |
| 536 | GTGVLTESNKKFLPFQQFGR | BCE | 0.7075 |
| 537 | TGVLTESNKKFLPFQQFGRD | BCE | 0.7244 |
| 538 | GVLTESNKKFLPFQQFGRDI | BCE | 0.7315 |
| 539 | VLTESNKKFLPFQQFGRDIA | BCE | 0.7675 |
| 540 | LTESNKKFLPFQQFGRDIAD | BCE | 0.7243 |
| 541 | TESNKKFLPFQQFGRDIADT | BCE | 0.7834 |
| 542 | ESNKKFLPFQQFGRDIADTT | BCE | 0.7749 |
| 543 | SNKKFLPFQQFGRDIADTTD | BCE | 0.7652 |
| 544 | NKKFLPFQQFGRDIADTTDA | BCE | 0.7909 |
| 545 | KKFLPFQQFGRDIADTTDAV | BCE | 0.7864 |
| 546 | KFLPFQQFGRDIADTTDAVR | BCE | 0.7269 |
| 547 | FLPFQQFGRDIADTTDAVRD | BCE | 0.7955 |
| 548 | LPFQQFGRDIADTTDAVRDP | BCE | 0.8247 |
| 549 | PFQQFGRDIADTTDAVRDPQ | BCE | 0.8488 |
| 550 | FQQFGRDIADTTDAVRDPQT | BCE | 0.8076 |
| 551 | QQFGRDIADTTDAVRDPQTL | BCE | 0.6989 |
| 552 | QFGRDIADTTDAVRDPQTLE | BCE | 0.7178 |
| 553 | FGRDIADTTDAVRDPQTLEI | BCE | 0.706 |
| 554 | GRDIADTTDAVRDPQTLEIL | BCE | 0.6191 |
| 555 | RDIADTTDAVRDPQTLEILD | BCE | 0.7448 |
| 556 | DIADTTDAVRDPQTLEILDI | BCE | 0.7843 |
| 557 | IADTTDAVRDPQTLEILDIT | BCE | 0.7876 |
| 558 | ADTTDAVRDPQTLEILDITP | BCE | 0.7469 |
| 559 | DTTDAVRDPQTLEILDITPC | BCE | 0.7568 |
| 560 | TTDAVRDPQTLEILDITPCS | BCE | 0.7327 |
| 561 | TDAVRDPQTLEILDITPCSF | BCE | 0.8058 |
| 562 | DAVRDPQTLEILDITPCSFG | BCE | 0.791 |
| 563 | AVRDPQTLEILDITPCSFGG | BCE | 0.7558 |
| 564 | VRDPQTLEILDITPCSFGGV | BCE | 0.6984 |
| 565 | RDPQTLEILDITPCSFGGVS | BCE | 0.7001 |
| 566 | DPQTLEILDITPCSFGGVSV | BCE | 0.711 |
| 567 | PQTLEILDITPCSFGGVSVI | BCE | 0.5747 |
| 568 | LEILDITPCSFGGVSVITPG | BCE | 0.5865 |
| 569 | DITPCSFGGVSVITPGTNTS | BCE | 0.6586 |
| 570 | ITPCSFGGVSVITPGTNTSN | BCE | 0.6667 |
| 571 | TPCSFGGVSVITPGTNTSNQ | BCE | 0.7638 |
| 572 | PCSFGGVSVITPGTNTSNQV | BCE | 0.7358 |
| 573 | CSFGGVSVITPGTNTSNQVA | BCE | 0.7168 |
| 574 | SFGGVSVITPGTNTSNQVAV | BCE | 0.7268 |
| 575 | FGGVSVITPGTNTSNQVAVL | BCE | 0.7154 |
| 576 | GGVSVITPGTNTSNQVAVLY | BCE | 0.7163 |
| 577 | GVSVITPGTNTSNQVAVLYQ | BCE | 0.7605 |
| 578 | VSVITPGTNTSNQVAVLYQD | BCE | 0.7138 |
| 579 | SVITPGTNTSNQVAVLYQDV | BCE | 0.7255 |
| 580 | VITPGTNTSNQVAVLYQDVN | BCE | 0.7607 |
| 581 | ITPGTNTSNQVAVLYQDVNC | BCE | 0.7641 |
| 582 | TPGTNTSNQVAVLYQDVNCT | BCE | 0.7686 |
| 583 | PGTNTSNQVAVLYQDVNCTE | BCE | 0.768 |
| 584 | GTNTSNQVAVLYQDVNCTEV | BCE | 0.7822 |
| 585 | TNTSNQVAVLYQDVNCTEVP | BCE | 0.7914 |
| 586 | NTSNQVAVLYQDVNCTEVPV | BCE | 0.7953 |
| 587 | TSNQVAVLYQDVNCTEVPVA | BCE | 0.763 |
| 588 | SNQVAVLYQDVNCTEVPVAI | BCE | 0.7186 |
| 589 | NQVAVLYQDVNCTEVPVAIH | BCE | 0.778 |
| 590 | QVAVLYQDVNCTEVPVAIHA | BCE | 0.7797 |
| 591 | VAVLYQDVNCTEVPVAIHAD | BCE | 0.8123 |
| 592 | AVLYQDVNCTEVPVAIHADQ | BCE | 0.827 |
| 593 | VLYQDVNCTEVPVAIHADQL | BCE | 0.8189 |
| 594 | LYQDVNCTEVPVAIHADQLT | BCE | 0.8136 |
| 595 | YQDVNCTEVPVAIHADQLTP | BCE | 0.7989 |
| 596 | QDVNCTEVPVAIHADQLTPT | BCE | 0.7859 |
| 597 | DVNCTEVPVAIHADQLTPTW | BCE | 0.7937 |
| 598 | VNCTEVPVAIHADQLTPTWR | BCE | 0.8061 |
| 599 | NCTEVPVAIHADQLTPTWRV | BCE | 0.7981 |
| 600 | CTEVPVAIHADQLTPTWRVY | BCE | 0.7621 |
| 601 | TEVPVAIHADQLTPTWRVYS | BCE | 0.7914 |
| 602 | EVPVAIHADQLTPTWRVYST | BCE | 0.7645 |
| 603 | VPVAIHADQLTPTWRVYSTG | BCE | 0.6757 |
| 604 | PVAIHADQLTPTWRVYSTGS | BCE | 0.6758 |
| 605 | VAIHADQLTPTWRVYSTGSN | BCE | 0.6922 |
| 606 | AIHADQLTPTWRVYSTGSNV | BCE | 0.7039 |
| 607 | IHADQLTPTWRVYSTGSNVF | BCE | 0.6684 |
| 608 | HADQLTPTWRVYSTGSNVFQ | BCE | 0.7864 |
| 609 | ADQLTPTWRVYSTGSNVFQT | BCE | 0.7698 |
| 610 | DQLTPTWRVYSTGSNVFQTR | BCE | 0.7175 |
| 611 | QLTPTWRVYSTGSNVFQTRA | BCE | 0.6313 |
| 612 | LTPTWRVYSTGSNVFQTRAG | BCE | 0.6611 |
| 613 | TPTWRVYSTGSNVFQTRAGC | BCE | 0.5644 |
| 614 | PTWRVYSTGSNVFQTRAGCL | BCE | 0.6334 |
| 615 | TWRVYSTGSNVFQTRAGCLI | BCE | 0.6665 |
| 616 | WRVYSTGSNVFQTRAGCLIG | BCE | 0.6818 |
| 617 | RVYSTGSNVFQTRAGCLIGA | BCE | 0.6615 |
| 618 | VYSTGSNVFQTRAGCLIGAE | BCE | 0.6456 |
| 619 | YSTGSNVFQTRAGCLIGAEH | BCE | 0.6786 |
| 620 | STGSNVFQTRAGCLIGAEHV | BCE | 0.6341 |
| 621 | TGSNVFQTRAGCLIGAEHVN | BCE | 0.6417 |
| 622 | GSNVFQTRAGCLIGAEHVNN | BCE | 0.6367 |
| 623 | SNVFQTRAGCLIGAEHVNNS | BCE | 0.6658 |
| 624 | NVFQTRAGCLIGAEHVNNSY | BCE | 0.6643 |
| 625 | VFQTRAGCLIGAEHVNNSYE | BCE | 0.7372 |
| 626 | FQTRAGCLIGAEHVNNSYEC | BCE | 0.7522 |
| 627 | QTRAGCLIGAEHVNNSYECD | BCE | 0.7925 |
| 628 | TRAGCLIGAEHVNNSYECDI | BCE | 0.7824 |
| 629 | RAGCLIGAEHVNNSYECDIP | BCE | 0.7966 |
| 630 | AGCLIGAEHVNNSYECDIPI | BCE | 0.7888 |
| 631 | GCLIGAEHVNNSYECDIPIG | BCE | 0.7602 |
| 632 | CLIGAEHVNNSYECDIPIGA | BCE | 0.781 |
| 633 | LIGAEHVNNSYECDIPIGAG | BCE | 0.7158 |
| 634 | IGAEHVNNSYECDIPIGAGI | BCE | 0.7033 |
| 635 | GAEHVNNSYECDIPIGAGIC | BCE | 0.7338 |
| 636 | AEHVNNSYECDIPIGAGICA | BCE | 0.7845 |
| 637 | EHVNNSYECDIPIGAGICAS | BCE | 0.7545 |
| 638 | HVNNSYECDIPIGAGICASY | BCE | 0.7725 |
| 639 | VNNSYECDIPIGAGICASYQ | BCE | 0.783 |
| 640 | NNSYECDIPIGAGICASYQT | BCE | 0.7637 |
| 641 | NSYECDIPIGAGICASYQTQ | BCE | 0.7211 |
| 642 | SYECDIPIGAGICASYQTQT | BCE | 0.6934 |
| 643 | YECDIPIGAGICASYQTQTN | BCE | 0.7765 |
| 644 | ECDIPIGAGICASYQTQTNS | BCE | 0.7547 |
| 645 | CDIPIGAGICASYQTQTNSP | BCE | 0.7589 |
| 646 | DIPIGAGICASYQTQTNSPR | BCE | 0.7102 |
| 647 | IPIGAGICASYQTQTNSPRR | BCE | 0.7421 |
| 648 | PIGAGICASYQTQTNSPRRA | BCE | 0.7705 |
| 649 | IGAGICASYQTQTNSPRRAR | BCE | 0.7562 |
| 650 | GAGICASYQTQTNSPRRARS | BCE | 0.8173 |
| 651 | AGICASYQTQTNSPRRARSV | BCE | 0.7638 |
| 652 | GICASYQTQTNSPRRARSVA | BCE | 0.7675 |
| 653 | ICASYQTQTNSPRRARSVAS | BCE | 0.7409 |
| 654 | CASYQTQTNSPRRARSVASQ | BCE | 0.7742 |
| 655 | ASYQTQTNSPRRARSVASQS | BCE | 0.8202 |
| 656 | SYQTQTNSPRRARSVASQSI | BCE | 0.8121 |
| 657 | YQTQTNSPRRARSVASQSII | BCE | 0.7783 |
| 658 | QTQTNSPRRARSVASQSIIA | BCE | 0.7976 |
| 659 | TQTNSPRRARSVASQSIIAY | BCE | 0.7567 |
| 660 | QTNSPRRARSVASQSIIAYT | BCE | 0.7571 |
| 661 | TNSPRRARSVASQSIIAYTM | BCE | 0.7677 |
| 662 | NSPRRARSVASQSIIAYTMS | BCE | 0.7721 |
| 663 | SPRRARSVASQSIIAYTMSL | BCE | 0.7989 |
| 664 | PRRARSVASQSIIAYTMSLG | BCE | 0.8022 |
| 665 | RRARSVASQSIIAYTMSLGA | BCE | 0.8068 |
| 666 | RARSVASQSIIAYTMSLGAE | BCE | 0.8062 |
| 667 | ARSVASQSIIAYTMSLGAEN | BCE | 0.7291 |
| 668 | RSVASQSIIAYTMSLGAENS | BCE | 0.7679 |
| 669 | SVASQSIIAYTMSLGAENSV | BCE | 0.5734 |
| 670 | ASQSIIAYTMSLGAENSVAY | BCE | 0.6583 |
| 671 | SQSIIAYTMSLGAENSVAYS | BCE | 0.6829 |
| 672 | QSIIAYTMSLGAENSVAYSN | BCE | 0.755 |
| 673 | SIIAYTMSLGAENSVAYSNN | BCE | 0.7602 |
| 674 | IIAYTMSLGAENSVAYSNNS | BCE | 0.6842 |
| 675 | IAYTMSLGAENSVAYSNNSI | BCE | 0.7069 |
| 676 | AYTMSLGAENSVAYSNNSIA | BCE | 0.6813 |
| 677 | YTMSLGAENSVAYSNNSIAI | BCE | 0.7371 |
| 678 | TMSLGAENSVAYSNNSIAIP | BCE | 0.6045 |
| 679 | MSLGAENSVAYSNNSIAIPT | BCE | 0.703 |
| 680 | SLGAENSVAYSNNSIAIPTN | BCE | 0.7134 |
| 681 | LGAENSVAYSNNSIAIPTNF | BCE | 0.7018 |
| 682 | GAENSVAYSNNSIAIPTNFT | BCE | 0.6801 |
| 683 | AENSVAYSNNSIAIPTNFTI | BCE | 0.7221 |
| 684 | ENSVAYSNNSIAIPTNFTIS | BCE | 0.6373 |
| 685 | NSVAYSNNSIAIPTNFTISV | BCE | 0.6272 |
| 686 | SVAYSNNSIAIPTNFTISVT | BCE | 0.6783 |
| 687 | VAYSNNSIAIPTNFTISVTT | BCE | 0.7398 |
| 688 | AYSNNSIAIPTNFTISVTTE | BCE | 0.7548 |
| 689 | YSNNSIAIPTNFTISVTTEI | BCE | 0.7747 |
| 690 | SNNSIAIPTNFTISVTTEIL | BCE | 0.7585 |
| 691 | NNSIAIPTNFTISVTTEILP | BCE | 0.7909 |
| 692 | NSIAIPTNFTISVTTEILPV | BCE | 0.7546 |
| 693 | SIAIPTNFTISVTTEILPVS | BCE | 0.7384 |
| 694 | IAIPTNFTISVTTEILPVSM | BCE | 0.7373 |
| 695 | AIPTNFTISVTTEILPVSMT | BCE | 0.7329 |
| 696 | IPTNFTISVTTEILPVSMTK | BCE | 0.7051 |
| 697 | PTNFTISVTTEILPVSMTKT | BCE | 0.7798 |
| 698 | TNFTISVTTEILPVSMTKTS | BCE | 0.7891 |
| 699 | NFTISVTTEILPVSMTKTSV | BCE | 0.6174 |
| 700 | FTISVTTEILPVSMTKTSVD | BCE | 0.726 |
| 701 | TISVTTEILPVSMTKTSVDC | BCE | 0.7175 |
| 702 | ISVTTEILPVSMTKTSVDCT | BCE | 0.7421 |
| 703 | SVTTEILPVSMTKTSVDCTM | BCE | 0.7409 |
| 704 | VTTEILPVSMTKTSVDCTMY | BCE | 0.7648 |
| 705 | TTEILPVSMTKTSVDCTMYI | BCE | 0.7366 |
| 706 | TEILPVSMTKTSVDCTMYIC | BCE | 0.7531 |
| 707 | EILPVSMTKTSVDCTMYICG | BCE | 0.7267 |
| 708 | ILPVSMTKTSVDCTMYICGD | BCE | 0.6751 |
| 709 | LPVSMTKTSVDCTMYICGDS | BCE | 0.7434 |
| 710 | PVSMTKTSVDCTMYICGDST | BCE | 0.7253 |
| 711 | VSMTKTSVDCTMYICGDSTE | BCE | 0.6442 |
| 712 | SMTKTSVDCTMYICGDSTEC | BCE | 0.7214 |
| 713 | MTKTSVDCTMYICGDSTECS | BCE | 0.713 |
| 714 | TKTSVDCTMYICGDSTECSN | BCE | 0.729 |
| 715 | KTSVDCTMYICGDSTECSNL | BCE | 0.772 |
| 716 | TSVDCTMYICGDSTECSNLL | BCE | 0.7757 |
| 717 | SVDCTMYICGDSTECSNLLL | BCE | 0.7014 |
| 718 | VDCTMYICGDSTECSNLLLQ | BCE | 0.6768 |
| 719 | DCTMYICGDSTECSNLLLQY | BCE | 0.6681 |
| 720 | CTMYICGDSTECSNLLLQYG | BCE | 0.6386 |
| 721 | TMYICGDSTECSNLLLQYGS | BCE | 0.6211 |
| 722 | MYICGDSTECSNLLLQYGSF | BCE | 0.6553 |
| 723 | YICGDSTECSNLLLQYGSFC | BCE | 0.6926 |
| 724 | ICGDSTECSNLLLQYGSFCT | BCE | 0.6915 |
| 725 | CGDSTECSNLLLQYGSFCTQ | BCE | 0.6817 |
| 726 | GDSTECSNLLLQYGSFCTQL | BCE | 0.6552 |
| 727 | DSTECSNLLLQYGSFCTQLN | BCE | 0.7334 |
| 728 | STECSNLLLQYGSFCTQLNR | BCE | 0.7553 |
| 729 | TECSNLLLQYGSFCTQLNRA | BCE | 0.7669 |
| 730 | ECSNLLLQYGSFCTQLNRAL | BCE | 0.7818 |
| 731 | CSNLLLQYGSFCTQLNRALT | BCE | 0.7608 |
| 732 | SNLLLQYGSFCTQLNRALTG | BCE | 0.6942 |
| 733 | NLLLQYGSFCTQLNRALTGI | BCE | 0.6907 |
| 734 | LLLQYGSFCTQLNRALTGIA | BCE | 0.7479 |
| 735 | LLQYGSFCTQLNRALTGIAV | BCE | 0.7214 |
| 736 | LQYGSFCTQLNRALTGIAVE | BCE | 0.7214 |
| 737 | QYGSFCTQLNRALTGIAVEQ | BCE | 0.7651 |
| 738 | YGSFCTQLNRALTGIAVEQD | BCE | 0.6858 |
| 739 | GSFCTQLNRALTGIAVEQDK | BCE | 0.668 |
| 740 | SFCTQLNRALTGIAVEQDKN | BCE | 0.6515 |
| 741 | FCTQLNRALTGIAVEQDKNT | BCE | 0.7189 |
| 742 | CTQLNRALTGIAVEQDKNTQ | BCE | 0.7281 |
| 743 | TQLNRALTGIAVEQDKNTQE | BCE | 0.806 |
| 744 | QLNRALTGIAVEQDKNTQEV | BCE | 0.8028 |
| 745 | LNRALTGIAVEQDKNTQEVF | BCE | 0.8103 |
| 746 | NRALTGIAVEQDKNTQEVFA | BCE | 0.6247 |
| 747 | RALTGIAVEQDKNTQEVFAQ | BCE | 0.5874 |
| 748 | ALTGIAVEQDKNTQEVFAQV | BCE | 0.7379 |
| 749 | LTGIAVEQDKNTQEVFAQVK | BCE | 0.7128 |
| 750 | TGIAVEQDKNTQEVFAQVKQ | BCE | 0.7864 |
| 751 | GIAVEQDKNTQEVFAQVKQI | BCE | 0.838 |
| 752 | IAVEQDKNTQEVFAQVKQIY | BCE | 0.8142 |
| 753 | AVEQDKNTQEVFAQVKQIYK | BCE | 0.7641 |
| 754 | VEQDKNTQEVFAQVKQIYKT | BCE | 0.7301 |
| 755 | EQDKNTQEVFAQVKQIYKTP | BCE | 0.7344 |
| 756 | QDKNTQEVFAQVKQIYKTPP | BCE | 0.7343 |
| 757 | DKNTQEVFAQVKQIYKTPPI | BCE | 0.6407 |
| 758 | KNTQEVFAQVKQIYKTPPIK | BCE | 0.7039 |
| 759 | NTQEVFAQVKQIYKTPPIKD | BCE | 0.7579 |
| 760 | TQEVFAQVKQIYKTPPIKDF | BCE | 0.6992 |
| 761 | QEVFAQVKQIYKTPPIKDFG | BCE | 0.7639 |
| 762 | EVFAQVKQIYKTPPIKDFGG | BCE | 0.7247 |
| 763 | VFAQVKQIYKTPPIKDFGGF | BCE | 0.6863 |
| 764 | FAQVKQIYKTPPIKDFGGFN | BCE | 0.7356 |
| 765 | AQVKQIYKTPPIKDFGGFNF | BCE | 0.7386 |
| 766 | QVKQIYKTPPIKDFGGFNFS | BCE | 0.7169 |
| 767 | VKQIYKTPPIKDFGGFNFSQ | BCE | 0.7037 |
| 768 | KQIYKTPPIKDFGGFNFSQI | BCE | 0.7027 |
| 769 | QIYKTPPIKDFGGFNFSQIL | BCE | 0.6867 |
| 770 | IYKTPPIKDFGGFNFSQILP | BCE | 0.7157 |
| 771 | YKTPPIKDFGGFNFSQILPD | BCE | 0.7679 |
| 772 | KTPPIKDFGGFNFSQILPDP | BCE | 0.8069 |
| 773 | TPPIKDFGGFNFSQILPDPS | BCE | 0.8258 |
| 774 | PPIKDFGGFNFSQILPDPSK | BCE | 0.8236 |
| 775 | PIKDFGGFNFSQILPDPSKP | BCE | 0.8236 |
| 776 | IKDFGGFNFSQILPDPSKPS | BCE | 0.8143 |
| 777 | KDFGGFNFSQILPDPSKPSK | BCE | 0.8444 |
| 778 | DFGGFNFSQILPDPSKPSKR | BCE | 0.8159 |
| 779 | FGGFNFSQILPDPSKPSKRS | BCE | 0.7972 |
| 780 | GGFNFSQILPDPSKPSKRSF | BCE | 0.808 |
| 781 | GFNFSQILPDPSKPSKRSFI | BCE | 0.7585 |
| 782 | FNFSQILPDPSKPSKRSFIE | BCE | 0.7921 |
| 783 | NFSQILPDPSKPSKRSFIED | BCE | 0.7989 |
| 784 | FSQILPDPSKPSKRSFIEDL | BCE | 0.7625 |
| 785 | SQILPDPSKPSKRSFIEDLL | BCE | 0.7835 |
| 786 | QILPDPSKPSKRSFIEDLLF | BCE | 0.7539 |
| 787 | ILPDPSKPSKRSFIEDLLFN | BCE | 0.7545 |
| 788 | LPDPSKPSKRSFIEDLLFNK | BCE | 0.7615 |
| 789 | PDPSKPSKRSFIEDLLFNKV | BCE | 0.738 |
| 790 | DPSKPSKRSFIEDLLFNKVT | BCE | 0.7287 |
| 791 | PSKPSKRSFIEDLLFNKVTL | BCE | 0.7973 |
| 792 | SKPSKRSFIEDLLFNKVTLA | BCE | 0.7216 |
| 793 | KPSKRSFIEDLLFNKVTLAD | BCE | 0.7657 |
| 794 | PSKRSFIEDLLFNKVTLADA | BCE | 0.8008 |
| 795 | SKRSFIEDLLFNKVTLADAG | BCE | 0.7741 |
| 796 | KRSFIEDLLFNKVTLADAGF | BCE | 0.7895 |
| 797 | RSFIEDLLFNKVTLADAGFI | BCE | 0.7939 |
| 798 | SFIEDLLFNKVTLADAGFIK | BCE | 0.7924 |
| 799 | FIEDLLFNKVTLADAGFIKQ | BCE | 0.801 |
| 800 | IEDLLFNKVTLADAGFIKQY | BCE | 0.7834 |
| 801 | EDLLFNKVTLADAGFIKQYG | BCE | 0.8019 |
| 802 | DLLFNKVTLADAGFIKQYGD | BCE | 0.8342 |
| 803 | LLFNKVTLADAGFIKQYGDC | BCE | 0.8035 |
| 804 | LFNKVTLADAGFIKQYGDCL | BCE | 0.7769 |
| 805 | FNKVTLADAGFIKQYGDCLG | BCE | 0.7697 |
| 806 | NKVTLADAGFIKQYGDCLGD | BCE | 0.8092 |
| 807 | KVTLADAGFIKQYGDCLGDI | BCE | 0.7833 |
| 808 | VTLADAGFIKQYGDCLGDIA | BCE | 0.7847 |
| 809 | TLADAGFIKQYGDCLGDIAA | BCE | 0.7862 |
| 810 | LADAGFIKQYGDCLGDIAAR | BCE | 0.7908 |
| 811 | ADAGFIKQYGDCLGDIAARD | BCE | 0.7868 |
| 812 | DAGFIKQYGDCLGDIAARDL | BCE | 0.7715 |
| 813 | AGFIKQYGDCLGDIAARDLI | BCE | 0.7707 |
| 814 | GFIKQYGDCLGDIAARDLIC | BCE | 0.8076 |
| 815 | FIKQYGDCLGDIAARDLICA | BCE | 0.8056 |
| 816 | IKQYGDCLGDIAARDLICAQ | BCE | 0.8067 |
| 817 | KQYGDCLGDIAARDLICAQK | BCE | 0.8153 |
| 818 | QYGDCLGDIAARDLICAQKF | BCE | 0.8087 |
| 819 | YGDCLGDIAARDLICAQKFN | BCE | 0.8124 |
| 820 | GDCLGDIAARDLICAQKFNG | BCE | 0.7945 |
| 821 | DCLGDIAARDLICAQKFNGL | BCE | 0.7891 |
| 822 | CLGDIAARDLICAQKFNGLT | BCE | 0.7928 |
| 823 | LGDIAARDLICAQKFNGLTV | BCE | 0.7758 |
| 824 | GDIAARDLICAQKFNGLTVL | BCE | 0.7789 |
| 825 | DIAARDLICAQKFNGLTVLP | BCE | 0.7906 |
| 826 | IAARDLICAQKFNGLTVLPP | BCE | 0.7769 |
| 827 | AARDLICAQKFNGLTVLPPL | BCE | 0.7823 |
| 828 | ARDLICAQKFNGLTVLPPLL | BCE | 0.7535 |
| 829 | RDLICAQKFNGLTVLPPLLT | BCE | 0.6259 |
| 830 | DLICAQKFNGLTVLPPLLTD | BCE | 0.7788 |
| 831 | LICAQKFNGLTVLPPLLTDE | BCE | 0.7168 |
| 832 | ICAQKFNGLTVLPPLLTDEM | BCE | 0.683 |
| 833 | CAQKFNGLTVLPPLLTDEMI | BCE | 0.6881 |
| 834 | AQKFNGLTVLPPLLTDEMIA | BCE | 0.6665 |
| 835 | QKFNGLTVLPPLLTDEMIAQ | BCE | 0.6917 |
| 836 | KFNGLTVLPPLLTDEMIAQY | BCE | 0.6079 |
| 837 | FNGLTVLPPLLTDEMIAQYT | BCE | 0.5484 |
| 838 | NGLTVLPPLLTDEMIAQYTS | BCE | 0.7026 |
| 839 | GLTVLPPLLTDEMIAQYTSA | BCE | 0.6748 |
| 840 | LTVLPPLLTDEMIAQYTSAL | BCE | 0.6649 |
| 841 | VLPPLLTDEMIAQYTSALLA | BCE | 0.5491 |
| 842 | LTDEMIAQYTSALLAGTITS | BCE | 0.5046 |
| 843 | TDEMIAQYTSALLAGTITSG | BCE | 0.6365 |
| 844 | DEMIAQYTSALLAGTITSGW | BCE | 0.5951 |
| 845 | EMIAQYTSALLAGTITSGWT | BCE | 0.5024 |
| 846 | MIAQYTSALLAGTITSGWTF | BCE | 0.5631 |
| 847 | IAQYTSALLAGTITSGWTFG | BCE | 0.6599 |
| 848 | AQYTSALLAGTITSGWTFGA | BCE | 0.631 |
| 849 | QYTSALLAGTITSGWTFGAG | BCE | 0.7389 |
| 850 | YTSALLAGTITSGWTFGAGA | BCE | 0.782 |
| 851 | TSALLAGTITSGWTFGAGAA | BCE | 0.8202 |
| 852 | SALLAGTITSGWTFGAGAAL | BCE | 0.7698 |
| 853 | ALLAGTITSGWTFGAGAALQ | BCE | 0.8034 |
| 854 | LLAGTITSGWTFGAGAALQI | BCE | 0.7784 |
| 855 | LAGTITSGWTFGAGAALQIP | BCE | 0.811 |
| 856 | AGTITSGWTFGAGAALQIPF | BCE | 0.8241 |
| 857 | GTITSGWTFGAGAALQIPFA | BCE | 0.8219 |
| 858 | TITSGWTFGAGAALQIPFAM | BCE | 0.7916 |
| 859 | ITSGWTFGAGAALQIPFAMQ | BCE | 0.8 |
| 860 | TSGWTFGAGAALQIPFAMQM | BCE | 0.7598 |
| 861 | SGWTFGAGAALQIPFAMQMA | BCE | 0.7678 |
| 862 | GWTFGAGAALQIPFAMQMAY | BCE | 0.7179 |
| 863 | WTFGAGAALQIPFAMQMAYR | BCE | 0.7024 |
| 864 | TFGAGAALQIPFAMQMAYRF | BCE | 0.6835 |
| 865 | FGAGAALQIPFAMQMAYRFN | BCE | 0.6943 |
| 866 | GAGAALQIPFAMQMAYRFNG | BCE | 0.7338 |
| 867 | AGAALQIPFAMQMAYRFNGI | BCE | 0.5864 |
| 868 | AALQIPFAMQMAYRFNGIGV | BCE | 0.6127 |
| 869 | ALQIPFAMQMAYRFNGIGVT | BCE | 0.5824 |
| 870 | LQIPFAMQMAYRFNGIGVTQ | BCE | 0.5336 |
| 871 | AMQMAYRFNGIGVTQNVLYE | BCE | 0.5372 |
| 872 | AYRFNGIGVTQNVLYENQKL | BCE | 0.6044 |
| 873 | RFNGIGVTQNVLYENQKLIA | BCE | 0.5801 |
| 874 | FNGIGVTQNVLYENQKLIAN | BCE | 0.6064 |
| 875 | NGIGVTQNVLYENQKLIANQ | BCE | 0.6061 |
| 876 | GIGVTQNVLYENQKLIANQF | BCE | 0.6958 |
| 877 | IGVTQNVLYENQKLIANQFN | BCE | 0.7543 |
| 878 | GVTQNVLYENQKLIANQFNS | BCE | 0.773 |
| 879 | VTQNVLYENQKLIANQFNSA | BCE | 0.747 |
| 880 | TQNVLYENQKLIANQFNSAI | BCE | 0.7562 |
| 881 | QNVLYENQKLIANQFNSAIG | BCE | 0.7047 |
| 882 | NVLYENQKLIANQFNSAIGK | BCE | 0.6027 |
| 883 | VLYENQKLIANQFNSAIGKI | BCE | 0.6263 |
| 884 | LYENQKLIANQFNSAIGKIQ | BCE | 0.6956 |
| 885 | YENQKLIANQFNSAIGKIQD | BCE | 0.6944 |
| 886 | ENQKLIANQFNSAIGKIQDS | BCE | 0.6278 |
| 887 | NQKLIANQFNSAIGKIQDSL | BCE | 0.5995 |
| 888 | QKLIANQFNSAIGKIQDSLS | BCE | 0.6415 |
| 889 | KLIANQFNSAIGKIQDSLSS | BCE | 0.5496 |
| 890 | LIANQFNSAIGKIQDSLSST | BCE | 0.5557 |
| 891 | AIGKIQDSLSSTASALGKLQ | BCE | 0.64 |
| 892 | IGKIQDSLSSTASALGKLQD | BCE | 0.6976 |
| 893 | GKIQDSLSSTASALGKLQDV | BCE | 0.7421 |
| 894 | KIQDSLSSTASALGKLQDVV | BCE | 0.7994 |
| 895 | IQDSLSSTASALGKLQDVVN | BCE | 0.8061 |
| 896 | QDSLSSTASALGKLQDVVNQ | BCE | 0.8033 |
| 897 | DSLSSTASALGKLQDVVNQN | BCE | 0.7827 |
| 898 | SLSSTASALGKLQDVVNQNA | BCE | 0.5355 |
| 899 | LSSTASALGKLQDVVNQNAQ | BCE | 0.6109 |
| 900 | SSTASALGKLQDVVNQNAQA | BCE | 0.6195 |
| 901 | STASALGKLQDVVNQNAQAL | BCE | 0.7677 |
| 902 | TASALGKLQDVVNQNAQALN | BCE | 0.7511 |
| 903 | ASALGKLQDVVNQNAQALNT | BCE | 0.7266 |
| 904 | SALGKLQDVVNQNAQALNTL | BCE | 0.7926 |
| 905 | ALGKLQDVVNQNAQALNTLV | BCE | 0.82 |
| 906 | LGKLQDVVNQNAQALNTLVK | BCE | 0.8255 |
| 907 | GKLQDVVNQNAQALNTLVKQ | BCE | 0.803 |
| 908 | KLQDVVNQNAQALNTLVKQL | BCE | 0.7917 |
| 909 | LQDVVNQNAQALNTLVKQLS | BCE | 0.8271 |
| 910 | QDVVNQNAQALNTLVKQLSS | BCE | 0.7226 |
| 911 | DVVNQNAQALNTLVKQLSSN | BCE | 0.7862 |
| 912 | VVNQNAQALNTLVKQLSSNF | BCE | 0.7861 |
| 913 | VNQNAQALNTLVKQLSSNFG | BCE | 0.7368 |
| 914 | NQNAQALNTLVKQLSSNFGA | BCE | 0.7316 |
| 915 | QNAQALNTLVKQLSSNFGAI | BCE | 0.7622 |
| 916 | NAQALNTLVKQLSSNFGAIS | BCE | 0.7363 |
| 917 | AQALNTLVKQLSSNFGAISS | BCE | 0.7414 |
| 918 | QALNTLVKQLSSNFGAISSV | BCE | 0.7568 |
| 919 | ALNTLVKQLSSNFGAISSVL | BCE | 0.7668 |
| 920 | LNTLVKQLSSNFGAISSVLN | BCE | 0.7502 |
| 921 | NTLVKQLSSNFGAISSVLND | BCE | 0.7312 |
| 922 | TLVKQLSSNFGAISSVLNDI | BCE | 0.717 |
| 923 | LVKQLSSNFGAISSVLNDIL | BCE | 0.7155 |
| 924 | VKQLSSNFGAISSVLNDILS | BCE | 0.5963 |
| 925 | KQLSSNFGAISSVLNDILSR | BCE | 0.5014 |
| 926 | QLSSNFGAISSVLNDILSRL | BCE | 0.6025 |
| 927 | LSSNFGAISSVLNDILSRLD | BCE | 0.6652 |
| 928 | SSNFGAISSVLNDILSRLDK | BCE | 0.7038 |
| 929 | SNFGAISSVLNDILSRLDKV | BCE | 0.7666 |
| 930 | NFGAISSVLNDILSRLDKVE | BCE | 0.7926 |
| 931 | FGAISSVLNDILSRLDKVEA | BCE | 0.8267 |
| 932 | GAISSVLNDILSRLDKVEAE | BCE | 0.8332 |
| 933 | AISSVLNDILSRLDKVEAEV | BCE | 0.8672 |
| 934 | ISSVLNDILSRLDKVEAEVQ | BCE | 0.8528 |
| 935 | SSVLNDILSRLDKVEAEVQI | BCE | 0.8456 |
| 936 | SVLNDILSRLDKVEAEVQID | BCE | 0.8538 |
| 937 | VLNDILSRLDKVEAEVQIDR | BCE | 0.8724 |
| 938 | LNDILSRLDKVEAEVQIDRL | BCE | 0.8579 |
| 939 | NDILSRLDKVEAEVQIDRLI | BCE | 0.8557 |
| 940 | DILSRLDKVEAEVQIDRLIT | BCE | 0.8424 |
| 941 | ILSRLDKVEAEVQIDRLITG | BCE | 0.8081 |
| 942 | LSRLDKVEAEVQIDRLITGR | BCE | 0.7983 |
| 943 | SRLDKVEAEVQIDRLITGRL | BCE | 0.784 |
| 944 | RLDKVEAEVQIDRLITGRLQ | BCE | 0.7958 |
| 945 | LDKVEAEVQIDRLITGRLQS | BCE | 0.8179 |
| 946 | DKVEAEVQIDRLITGRLQSL | BCE | 0.8214 |
| 947 | KVEAEVQIDRLITGRLQSLQ | BCE | 0.835 |
| 948 | VEAEVQIDRLITGRLQSLQT | BCE | 0.8357 |
| 949 | EAEVQIDRLITGRLQSLQTY | BCE | 0.8187 |
| 950 | AEVQIDRLITGRLQSLQTYV | BCE | 0.7698 |
| 951 | EVQIDRLITGRLQSLQTYVT | BCE | 0.7752 |
| 952 | VQIDRLITGRLQSLQTYVTQ | BCE | 0.8093 |
| 953 | QIDRLITGRLQSLQTYVTQQ | BCE | 0.8437 |
| 954 | IDRLITGRLQSLQTYVTQQL | BCE | 0.8195 |
| 955 | DRLITGRLQSLQTYVTQQLI | BCE | 0.8 |
| 956 | RLITGRLQSLQTYVTQQLIR | BCE | 0.7806 |
| 957 | LITGRLQSLQTYVTQQLIRA | BCE | 0.7508 |
| 958 | ITGRLQSLQTYVTQQLIRAA | BCE | 0.8045 |
| 959 | TGRLQSLQTYVTQQLIRAAE | BCE | 0.812 |
| 960 | GRLQSLQTYVTQQLIRAAEI | BCE | 0.8372 |
| 961 | RLQSLQTYVTQQLIRAAEIR | BCE | 0.7994 |
| 962 | LQSLQTYVTQQLIRAAEIRA | BCE | 0.8104 |
| 963 | QSLQTYVTQQLIRAAEIRAS | BCE | 0.8189 |
| 964 | SLQTYVTQQLIRAAEIRASA | BCE | 0.8146 |
| 965 | LQTYVTQQLIRAAEIRASAN | BCE | 0.8198 |
| 966 | QTYVTQQLIRAAEIRASANL | BCE | 0.8186 |
| 967 | TYVTQQLIRAAEIRASANLA | BCE | 0.8215 |
| 968 | YVTQQLIRAAEIRASANLAA | BCE | 0.7759 |
| 969 | VTQQLIRAAEIRASANLAAT | BCE | 0.8352 |
| 970 | TQQLIRAAEIRASANLAATK | BCE | 0.8526 |
| 971 | QQLIRAAEIRASANLAATKM | BCE | 0.8389 |
| 972 | QLIRAAEIRASANLAATKMS | BCE | 0.8221 |
| 973 | LIRAAEIRASANLAATKMSE | BCE | 0.8603 |
| 974 | IRAAEIRASANLAATKMSEC | BCE | 0.8162 |
| 975 | RAAEIRASANLAATKMSECV | BCE | 0.831 |
| 976 | AAEIRASANLAATKMSECVL | BCE | 0.8184 |
| 977 | AEIRASANLAATKMSECVLG | BCE | 0.8125 |
| 978 | EIRASANLAATKMSECVLGQ | BCE | 0.796 |
| 979 | IRASANLAATKMSECVLGQS | BCE | 0.7649 |
| 980 | RASANLAATKMSECVLGQSK | BCE | 0.7556 |
| 981 | ASANLAATKMSECVLGQSKR | BCE | 0.753 |
| 982 | SANLAATKMSECVLGQSKRV | BCE | 0.7598 |
| 983 | ANLAATKMSECVLGQSKRVD | BCE | 0.7752 |
| 984 | NLAATKMSECVLGQSKRVDF | BCE | 0.7639 |
| 985 | LAATKMSECVLGQSKRVDFC | BCE | 0.8004 |
| 986 | AATKMSECVLGQSKRVDFCG | BCE | 0.7824 |
| 987 | ATKMSECVLGQSKRVDFCGK | BCE | 0.7815 |
| 988 | TKMSECVLGQSKRVDFCGKG | BCE | 0.7764 |
| 989 | KMSECVLGQSKRVDFCGKGY | BCE | 0.8149 |
| 990 | MSECVLGQSKRVDFCGKGYH | BCE | 0.8 |
| 991 | SECVLGQSKRVDFCGKGYHL | BCE | 0.8087 |
| 992 | ECVLGQSKRVDFCGKGYHLM | BCE | 0.782 |
| 993 | CVLGQSKRVDFCGKGYHLMS | BCE | 0.7368 |
| 994 | VLGQSKRVDFCGKGYHLMSF | BCE | 0.7153 |
| 995 | LGQSKRVDFCGKGYHLMSFP | BCE | 0.7297 |
| 996 | GQSKRVDFCGKGYHLMSFPQ | BCE | 0.7035 |
| 997 | QSKRVDFCGKGYHLMSFPQS | BCE | 0.701 |
| 998 | SKRVDFCGKGYHLMSFPQSA | BCE | 0.6782 |
| 999 | KRVDFCGKGYHLMSFPQSAP | BCE | 0.7199 |
| 1000 | RVDFCGKGYHLMSFPQSAPH | BCE | 0.6606 |
| 1001 | VDFCGKGYHLMSFPQSAPHG | BCE | 0.5773 |
| 1002 | DFCGKGYHLMSFPQSAPHGV | BCE | 0.5878 |
| 1003 | FCGKGYHLMSFPQSAPHGVV | BCE | 0.5903 |
| 1004 | LMSFPQSAPHGVVFLHVTYV | BCE | 0.5769 |
| 1005 | MSFPQSAPHGVVFLHVTYVP | BCE | 0.5359 |
| 1006 | FPQSAPHGVVFLHVTYVPAQ | BCE | 0.5544 |
| 1007 | PQSAPHGVVFLHVTYVPAQE | BCE | 0.5717 |
| 1008 | QSAPHGVVFLHVTYVPAQEK | BCE | 0.7313 |
| 1009 | SAPHGVVFLHVTYVPAQEKN | BCE | 0.7165 |
| 1010 | APHGVVFLHVTYVPAQEKNF | BCE | 0.7283 |
| 1011 | PHGVVFLHVTYVPAQEKNFT | BCE | 0.7343 |
| 1012 | HGVVFLHVTYVPAQEKNFTT | BCE | 0.6825 |
| 1013 | GVVFLHVTYVPAQEKNFTTA | BCE | 0.7409 |
| 1014 | VVFLHVTYVPAQEKNFTTAP | BCE | 0.7947 |
| 1015 | VFLHVTYVPAQEKNFTTAPA | BCE | 0.7589 |
| 1016 | FLHVTYVPAQEKNFTTAPAI | BCE | 0.8291 |
| 1017 | LHVTYVPAQEKNFTTAPAIC | BCE | 0.8244 |
| 1018 | HVTYVPAQEKNFTTAPAICH | BCE | 0.8362 |
| 1019 | VTYVPAQEKNFTTAPAICHD | BCE | 0.7031 |
| 1020 | TYVPAQEKNFTTAPAICHDG | BCE | 0.6809 |
| 1021 | YVPAQEKNFTTAPAICHDGK | BCE | 0.7388 |
| 1022 | VPAQEKNFTTAPAICHDGKA | BCE | 0.6846 |
| 1023 | PAQEKNFTTAPAICHDGKAH | BCE | 0.7953 |
| 1024 | AQEKNFTTAPAICHDGKAHF | BCE | 0.8323 |
| 1025 | QEKNFTTAPAICHDGKAHFP | BCE | 0.7477 |
| 1026 | EKNFTTAPAICHDGKAHFPR | BCE | 0.7883 |
| 1027 | KNFTTAPAICHDGKAHFPRE | BCE | 0.7662 |
| 1028 | NFTTAPAICHDGKAHFPREG | BCE | 0.7313 |
| 1029 | FTTAPAICHDGKAHFPREGV | BCE | 0.6731 |
| 1030 | TTAPAICHDGKAHFPREGVF | BCE | 0.6198 |
| 1031 | TAPAICHDGKAHFPREGVFV | BCE | 0.5169 |
| 1032 | CHDGKAHFPREGVFVSNGTH | BCE | 0.643 |
| 1033 | HDGKAHFPREGVFVSNGTHW | BCE | 0.7038 |
| 1034 | DGKAHFPREGVFVSNGTHWF | BCE | 0.6658 |
| 1035 | GKAHFPREGVFVSNGTHWFV | BCE | 0.5352 |
| 1036 | KAHFPREGVFVSNGTHWFVT | BCE | 0.5428 |
| 1037 | FPREGVFVSNGTHWFVTQRN | BCE | 0.7292 |
| 1038 | PREGVFVSNGTHWFVTQRNF | BCE | 0.7455 |
| 1039 | REGVFVSNGTHWFVTQRNFY | BCE | 0.7004 |
| 1040 | EGVFVSNGTHWFVTQRNFYE | BCE | 0.595 |
| 1041 | GVFVSNGTHWFVTQRNFYEP | BCE | 0.7007 |
| 1042 | VFVSNGTHWFVTQRNFYEPQ | BCE | 0.7238 |
| 1043 | FVSNGTHWFVTQRNFYEPQI | BCE | 0.7813 |
| 1044 | VSNGTHWFVTQRNFYEPQII | BCE | 0.7759 |
| 1045 | SNGTHWFVTQRNFYEPQIIT | BCE | 0.7465 |
| 1046 | NGTHWFVTQRNFYEPQIITT | BCE | 0.7615 |
| 1047 | GTHWFVTQRNFYEPQIITTD | BCE | 0.7776 |
| 1048 | THWFVTQRNFYEPQIITTDN | BCE | 0.7982 |
| 1049 | HWFVTQRNFYEPQIITTDNT | BCE | 0.8012 |
| 1050 | WFVTQRNFYEPQIITTDNTF | BCE | 0.7872 |
| 1051 | FVTQRNFYEPQIITTDNTFV | BCE | 0.7789 |
| 1052 | VTQRNFYEPQIITTDNTFVS | BCE | 0.775 |
| 1053 | TQRNFYEPQIITTDNTFVSG | BCE | 0.7662 |
| 1054 | QRNFYEPQIITTDNTFVSGN | BCE | 0.7579 |
| 1055 | RNFYEPQIITTDNTFVSGNC | BCE | 0.7705 |
| 1056 | NFYEPQIITTDNTFVSGNCD | BCE | 0.7498 |
| 1057 | FYEPQIITTDNTFVSGNCDV | BCE | 0.6872 |
| 1058 | YEPQIITTDNTFVSGNCDVV | BCE | 0.6925 |
| 1059 | EPQIITTDNTFVSGNCDVVI | BCE | 0.7583 |
| 1060 | PQIITTDNTFVSGNCDVVIG | BCE | 0.611 |
| 1061 | QIITTDNTFVSGNCDVVIGI | BCE | 0.6874 |
| 1062 | IITTDNTFVSGNCDVVIGIV | BCE | 0.712 |
| 1063 | ITTDNTFVSGNCDVVIGIVN | BCE | 0.7061 |
| 1064 | TTDNTFVSGNCDVVIGIVNN | BCE | 0.7915 |
| 1065 | TDNTFVSGNCDVVIGIVNNT | BCE | 0.773 |
| 1066 | DNTFVSGNCDVVIGIVNNTV | BCE | 0.7317 |
| 1067 | NTFVSGNCDVVIGIVNNTVY | BCE | 0.66 |
| 1068 | TFVSGNCDVVIGIVNNTVYD | BCE | 0.7202 |
| 1069 | FVSGNCDVVIGIVNNTVYDP | BCE | 0.7447 |
| 1070 | VSGNCDVVIGIVNNTVYDPL | BCE | 0.7743 |
| 1071 | SGNCDVVIGIVNNTVYDPLQ | BCE | 0.7627 |
| 1072 | GNCDVVIGIVNNTVYDPLQP | BCE | 0.7837 |
| 1073 | NCDVVIGIVNNTVYDPLQPE | BCE | 0.8474 |
| 1074 | CDVVIGIVNNTVYDPLQPEL | BCE | 0.8493 |
| 1075 | DVVIGIVNNTVYDPLQPELD | BCE | 0.8634 |
| 1076 | VVIGIVNNTVYDPLQPELDS | BCE | 0.906 |
| 1077 | VIGIVNNTVYDPLQPELDSF | BCE | 0.9356 |
| 1078 | IGIVNNTVYDPLQPELDSFK | BCE | 0.8886 |
| 1079 | GIVNNTVYDPLQPELDSFKE | BCE | 0.8784 |
| 1080 | IVNNTVYDPLQPELDSFKEE | BCE | 0.9073 |
| 1081 | VNNTVYDPLQPELDSFKEEL | BCE | 0.9115 |
| 1082 | NNTVYDPLQPELDSFKEELD | BCE | 0.9022 |
| 1083 | NTVYDPLQPELDSFKEELDK | BCE | 0.873 |
| 1084 | TVYDPLQPELDSFKEELDKY | BCE | 0.8613 |
| 1085 | VYDPLQPELDSFKEELDKYF | BCE | 0.8335 |
| 1086 | YDPLQPELDSFKEELDKYFK | BCE | 0.825 |
| 1087 | DPLQPELDSFKEELDKYFKN | BCE | 0.8393 |
| 1088 | PLQPELDSFKEELDKYFKNH | BCE | 0.8382 |
| 1089 | LQPELDSFKEELDKYFKNHT | BCE | 0.8428 |
| 1090 | QPELDSFKEELDKYFKNHTS | BCE | 0.8611 |
| 1091 | PELDSFKEELDKYFKNHTSP | BCE | 0.8406 |
| 1092 | ELDSFKEELDKYFKNHTSPD | BCE | 0.7868 |
| 1093 | LDSFKEELDKYFKNHTSPDV | BCE | 0.7562 |
| 1094 | DSFKEELDKYFKNHTSPDVD | BCE | 0.7185 |
| 1095 | SFKEELDKYFKNHTSPDVDL | BCE | 0.7701 |
| 1096 | FKEELDKYFKNHTSPDVDLG | BCE | 0.7827 |
| 1097 | KEELDKYFKNHTSPDVDLGD | BCE | 0.8084 |
| 1098 | EELDKYFKNHTSPDVDLGDI | BCE | 0.8128 |
| 1099 | ELDKYFKNHTSPDVDLGDIS | BCE | 0.8202 |
| 1100 | LDKYFKNHTSPDVDLGDISG | BCE | 0.8093 |
| 1101 | DKYFKNHTSPDVDLGDISGI | BCE | 0.7744 |
| 1102 | KYFKNHTSPDVDLGDISGIN | BCE | 0.7666 |
| 1103 | YFKNHTSPDVDLGDISGINA | BCE | 0.7489 |
| 1104 | FKNHTSPDVDLGDISGINAS | BCE | 0.7671 |
| 1105 | KNHTSPDVDLGDISGINASV | BCE | 0.792 |
| 1106 | NHTSPDVDLGDISGINASVV | BCE | 0.77 |
| 1107 | HTSPDVDLGDISGINASVVN | BCE | 0.7613 |
| 1108 | TSPDVDLGDISGINASVVNI | BCE | 0.8 |
| 1109 | SPDVDLGDISGINASVVNIQ | BCE | 0.8207 |
| 1110 | PDVDLGDISGINASVVNIQK | BCE | 0.837 |
| 1111 | DVDLGDISGINASVVNIQKE | BCE | 0.8147 |
| 1112 | VDLGDISGINASVVNIQKEI | BCE | 0.8071 |
| 1113 | DLGDISGINASVVNIQKEID | BCE | 0.8142 |
| 1114 | LGDISGINASVVNIQKEIDR | BCE | 0.742 |
| 1115 | GDISGINASVVNIQKEIDRL | BCE | 0.7588 |
