# Supplementary material for: Multiepitope Subunit Vaccine Design against COVID-19 Based on the Spike Protein of SARS-CoV-2: An In Silico Analysis
Source: J Immunol Res. 2020 Nov 19;2020:8893483. doi: 10.1155/2020/8893483 (PMC7678744; doi:10.1155/2020/8893483)
Supplement: Supplementary Materials — Table S1: epitope filtration steps to finalize HLA I epitopes in the SARS-CoV-2 spike glycoprotein sequence. Table S2: epitope filtration steps to finalize HLA II epitopes in the SARS-CoV-2 spike glycoprotein sequence after PREDIVAC and other tools. Table S3: epitope filtration steps to finalize HLA II epitopes in the SARS-CoV-2 spike glycoprotein sequence after NetMHCIIpan and other tools. Table S4: predicted B-cell linear epitopes in the SARS-CoV-2 glycoprotein with probability values. Table S5: predicted discontinuous B-cell epitopes in the multiepitope vaccine according to the DiscoTope server. Figure S1: graphs obtained after molecular docking between vaccine and TLR3 structures. Figure S2: graphs obtained after applying refinements on the top vaccine-TLR3 docked structure. [file 8893483.f1.zip › Supplementary Table 5.docx]

Supplementary Table 5

| **aa position** | **Residue** | **Score** |
| --- | --- | --- |
| 21 | LEU | -2.795 |
| 24 | LYS | -1.163 |
| 25 | ILE | -1.55 |
| 26 | PHE | -2.166 |
| 27 | SER | 0.003 |
| 28 | TYR | -0.22 |
| 29 | THR | -1.828 |
| 30 | GLU | -0.007 |
| 31 | SER | -1.891 |
| 32 | LEU | -3.406 |
| 33 | ALA | -2.432 |
| 34 | GLY | -1.301 |
| 35 | LYS | -1.761 |
| 36 | ARG | -0.737 |
| 37 | GLU | -2.707 |
| 44 | LYS | -2.97 |
| 45 | ASN | 0.18 |
| 46 | GLY | -0.228 |
| 47 | ALA | -0.477 |
| 48 | THR | -1.429 |
| 49 | PHE | -2.201 |
| 50 | GLN | -2.028 |
| 51 | VAL | -2.883 |
| 52 | GLU | -0.898 |
| 53 | VAL | -3.303 |
| 54 | PRO | -0.521 |
| 55 | GLY | -1.917 |
| 56 | SER | 0.629 |
| 57 | GLN | 1.541 |
| 58 | HIS | -0.672 |
| 59 | ILE | -1.154 |
| 60 | ASP | 1.268 |
| 61 | SER | -0.162 |
| 62 | GLN | -2.114 |
| 63 | LYS | -1.134 |
| 64 | LYS | -0.8 |
| 65 | ALA | -2.891 |
| 67 | GLU | -1.918 |
| 68 | ARG | -3.052 |
| 161 | GLY | -2.987 |
| 162 | PRO | -0.878 |
| 163 | GLY | -0.33 |
| 164 | PRO | 2.25 |
| 165 | GLY | -0.522 |
| 166 | TYR | -0.492 |
| 167 | GLN | 0.378 |
| 169 | TYR | -3.089 |
| 178 | PRO | -3.03 |
| 180 | GLN | -1.553 |
| 181 | ILE | -2.424 |
| 182 | ILE | -1.845 |
| 183 | THR | 1.093 |
| 184 | THR | -1.474 |
| 185 | ASP | 1.016 |
| 186 | ASN | -0.413 |
| 187 | THR | 0.566 |
| 188 | PHE | 0.867 |
| 189 | GLY | 0.692 |
| 190 | PRO | -0.301 |
| 191 | GLY | 0.766 |
| 192 | PRO | -0.757 |
| 193 | GLY | -1.22 |
| 194 | TYR | -1.923 |
| 195 | PHE | -2.952 |
| 196 | LYS | -3.35 |
| 205 | GLY | -3.32 |
| 206 | PRO | -0.218 |
| 207 | GLY | -2.279 |
| 208 | GLY | -1.901 |
| 209 | ILE | -1.058 |
| 210 | ASN | -0.3 |
| 212 | THR | -3.477 |
| 213 | ARG | -1.838 |
| 214 | PHE | -2.364 |
| 251 | GLY | -2.985 |
| 252 | PRO | -2.234 |
| 274 | PRO | -3.466 |
